# Synthesis and Inhibitory Assessment of ACE2 Inhibitors for SARS-CoV-2: An *In Silico* and *In Vitro* Study

**DOI:** 10.1021/acs.joc.5c00918

**Published:** 2025-07-21

**Authors:** Xiaoyun Wang, Jieyu He, Layla Hosseini-Gerami, Morgan Thomas, Stephen Thompson, Joseph Ford, Sebastiano Ortalli, Zijun Chen, Gianluca Destro, Andreas Bender, Franklin Aigbirhio, Véronique Gouverneur

**Affiliations:** † Chemistry Research Laboratory, 6396 University of Oxford, 12 Mansfield Road, Oxford OX1 3TA, U.K.; ‡ Molecular Imaging Chemistry Laboratory, Wolfson Brain Imaging Centre, Department of Clinical Neurosciences, 2152 University of Cambridge, Cambridge CB2 0QQ, U.K.; § Centre for Molecular Informatics, Department of Chemistry, 2152 University of Cambridge, Cambridge CB2 1EW, U.K.; ⊥ College of Medicine and Health Sciences, Khalifa University of Science and Technology, Abu Dhabi 127788, UAE; ¶ STAR-UBB Institute, Babeş-Bolyai University, , Cluj-Napoca 400084, Romania

## Abstract

The angiotensin-converting enzyme 2 (ACE2) is pivotal as the cellular receptor for SARS-CoV-2 (severe acute respiratory syndrome coronavirus 2), the virus responsible for COVID-19. This study presents a novel synthetic route for four analogues of MLN-4760, a known inhibitor of ACE2, guided by *in silico* docking predictions. These synthetic advances enabled in vitro pIC_50_ assays confirming the inhibitory potency of the synthesized analogues. Lastly, this route was applied to the synthesis of novel ^18^F-labeled ACE2 inhibitors for PET imaging applications.

## Graphical abstract

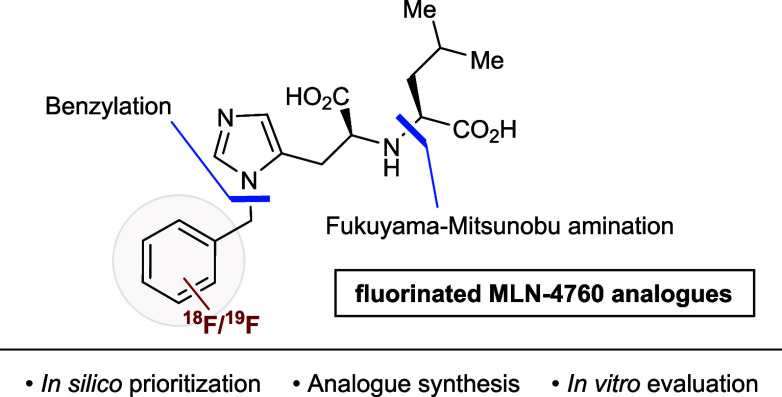

In March 2020, the World Health Organization declared COVID-19 a pandemic due to the rapid global spread of severe acute respiratory syndrome coronavirus 2 (SARS-CoV-2), resulting in a significant global health and economic crisis.^1^ SARS-CoV-2 infects host cells by binding of the spike protein to angiotensin-converting enzyme 2 (ACE2), a zinc–metalloprotease expressed in various organs, including the lungs, heart, kidneys, and intestines.^2^ ACE2 is considered a potential biomarker for disease progression and severity in COVID-19, but the extent to which its expression level relates to infection remains unclear. Clinical studies on hospitalized patients have suggested a positive correlation between plasma ACE2 levels and the severity of COVID-19 infection,^3^ while ACE2 deficiency may also worsen long COVID-19 symptoms, especially in patients with underlying conditions such as diabetes, hypertension and heart disease.^4^

Current ACE2 detection methods include immunohistochemistry and serological testing, but these do not allow for dynamic visualization and assessment of ACE2 biodistribution.^5^ Molecular imaging, particularly noninvasive positron emission tomography (PET) imaging, offers a high-sensitivity alternative to quantitatively monitor ACE2 expression.^6^ Various studies have illustrated the value of PET imaging with ACE2 radiotracers prepared by derivatization of the ACE2-targeting peptide DX600 with radiometal chelators, including [^67^Ga]­HBED-CC-DX600, [^68^Ga]­NOTA-PEP4, [^68^Ga]­HZ20, [^64^Cu]­HZ20, and [^18^F]­AlF-DX600-BCH (Figure 1A).^7^ These tracers display significant accumulation in ACE2-expressing tissues, and attenuation of signal in organs affected by COVID-19.^7b^ High uptake in tissues such as the nasal mucosa and kidneys were observed, contrasting to lower accumulation of radiotracer in the lungs and heart, in line with known ACE2 expression patterns.^7b,7c^

**Figure 1:**
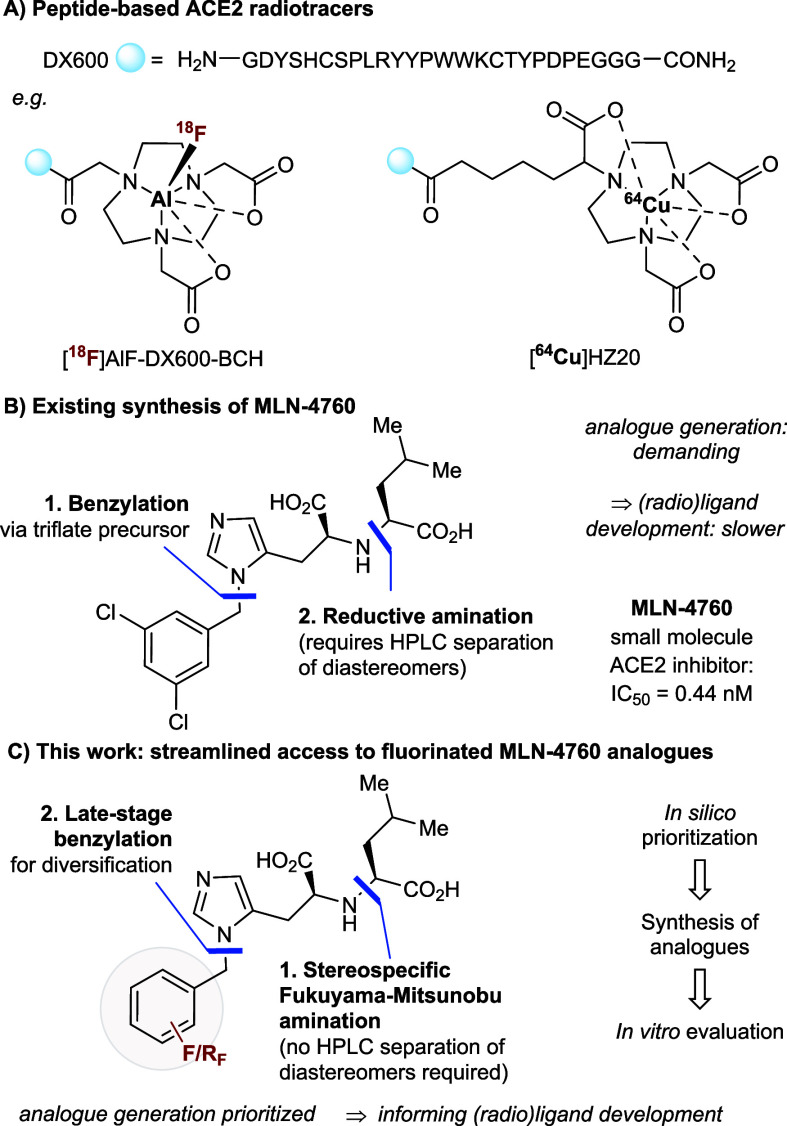
(A) Peptide-derived ACE2 PET radiotracers. (B) Established synthesis of MLN-4760. (C) Synthesis of diversified fluorinated MLN-4760 analogues (this work).

Despite these advances, the development of new-generation radiotracers with improved properties, such as higher affinity for the ACE2 receptor (IC_50_(DX600) = 113.6 nM) as well as reduced bone uptake, remains highly desirable.^5d^ With the aim to develop a novel ACE2 PET radiotracer, analogues of the potent small molecule ACE2 inhibitor MLN-4760 ((*S*,*S*)*-*2-(1-carboxy-2-(3-(3,5-dichlorobenzyl)-3H-imidazol-4-yl)-ethylamino)-4-methylpentanoic acid) (IC_50_ = 0.44 nM) were selected as possible candidates for investigation (Figure 1B).^8^ The original synthesis of MLN-4760 reported by Dales et al. proved suboptimal for radiotracer design. First, the early stage installation of a benzyl group, which is an ideal site for radiolabeling with the PET radioisotopes of fluorine-18 or carbon-11, would demand a multistep synthesis of precursors and reference standards for every candidate radiotracer under consideration.^9^ This imposes a significant bottleneck in the tracer discovery process, where multiple labeling strategies are envisaged, requiring the synthesis of multiple classes of labeling precursors. Furthermore, an unselective reductive amination step requires preparative HPLC separation and purification of diastereomers.^8^ With these challenges in mind, our aim was to develop a novel divergent route to analogues of MLN-4760 to inform future ACE2 radiotracer development (Figure 1C).

To begin our studies, we formulated a set of 31 potential analogues of MLN-4760 for initial *in silico* evaluation (Figure S1.3). Such screening would guide our selection of the identity and position of the radiolabel (fluorine-18 or carbon-11), and allow us to prioritize the synthesis of a subset of analogues for in vitro studies. Computational docking and Relative Binding Free Energy (RBFE) methods were employed sequentially to predict binding affinity changes and pIC_50_ values for each candidate. After validation of the docking model using literature pIC_50_ data for MLN-4760 analogues with an ACE2 crystal structure (PDB 1R4L),^10^ 14 fluorinated analogues were carried forward for RBFE analysis, which better accounts for interactions and dynamic processes (Table S1.2). Applying this protocol to these 14 compounds yielded predicted pIC_50_ values, which were corrected using linear regression between the known and experimentally determined pIC_50_ for MLN-4760 analogues. This enabled us to shortlist four fluorinated candidates for synthesis (Figure 2). With this information in hand, we next focused our attention on the synthesis of these four MLN-4760 analogues (*S*,*S*)-**1–**(*S*,*S*)-**4**, selecting the 4-fluorobenzyl analogue (*S*,*S*)-**1** as the representative model compound.

**Figure 2:**
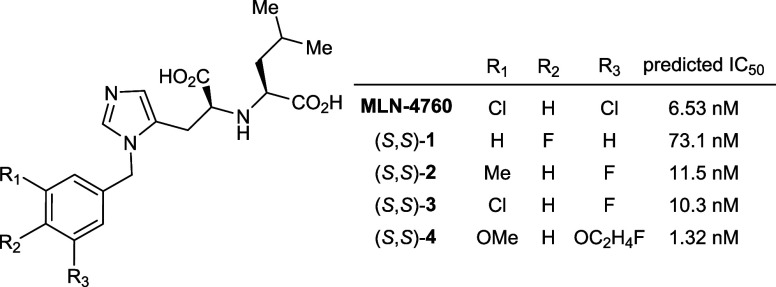
Structural analogues of MLN-4760 ((*S*,*S*)-**1**–(*S*,*S*)-**4**) selected as potential ACE2 inhibitors and their predicted IC_50_ values.

Early attempts applied the original synthetic method for MLN-4760 disclosed by Dales et al.; in our hands, the key reductive amination step delivered a low yield of the desired product (31%) (Table S2.1).^8^ Additionally, the process resulted in a mixture of diastereomers (57:43 *d*.*r*., determined by ^1^H NMR), which required separation by silica gel column chromatography and preparative HPLC to isolate (*S*,*S*)-**1** in sufficient purity (Scheme S2.1). These difficulties prompted us to modify the synthesis. With the aim to improve the yield and stereoselectivity of the key C–N bond forming step, we opted for the Fukuyama-Mitsunobu reaction based on its well-documented stereospecific S_N_2 mechanism.^11^ Furthermore, we envisaged that resequencing the amination and benzylation steps would facilitate analogue generation from a single common intermediate via late-stage benzylation, thus reducing synthetic burden and expediting access to analogues (*S*,*S*)-**1**–(*S*,*S*)-**4**.

A synthesis of (*S*,*S*)-**1** with a route featuring a key Fukuyama-Mitsunobu amination was therefore initiated (Scheme 1). Starting with the histidine derivative (*S*)-**5**, 2-nitrobenzenesulfonyl (Ns) groups were first installed at both the N1-position and primary amine with 2-nitrobenzenesulfonyl chloride (NsCl) in the presence of triethylamine in dichloromethane, affording doubly Ns-protected intermediate (*S*)-**6** in 76% yield. The Ns group served as a protecting and activating group for the Fukuyama-Mitsunobu amination.^12^ This reaction, involving the di-Ns-protected histidine derivative (*S*)-**6** and benzyl (*R*)-2-hydroxy-4-methylpentanoate ((*R*)-**7**), was carefully optimized with key reaction parameters investigated (Table 1). Preliminary investigation demonstrated that diisopropyl azodicarboxylate (DIAD) as an activator in THF at room temperature could be effective (Scheme S2.2). Optimization of both the number of equivalents of (*R*)-**7** and the time at which each reagent is added revealed that sequential addition of *(S)*-**6** and *(R)*-**7** to a stirred mixture of DIAD and PPh_3_ in THF after a delay of 1 and 2 h, respectively, led to the highest yields of (*S,S*)-**8** (Table 1, entry 6). The Fukuyama-Mitsunobu amination was thus performed under these optimized conditions, resulting in the formation of the histidine-leucine derivative (*S,S*)-**8** with a 55% yield and >20:1 *d.r.* (determined by ^1^H NMR) for the desired diastereomer (Scheme 1b). Subsequent benzylation of (*S,S*)-di-Ns-protected **8** did not yield the desired product, likely due to the presence of the electron-withdrawing Ns group (Scheme S2.3). The Ns groups were therefore cleaved using thiophenol (PhSH), successfully producing the histidine-leucine derivative (*S,S*)-**9** in 70% yield (Scheme 1c).^12^ In advance of N3-benzylation and to avoid formation of the undesired N1-regioisomer, Boc groups were installed at the N1-position of the imidazole and at the Leu-derived secondary amine (Scheme 1d).^13^ This crude material was carried forward for benzylation with 4-fluorobenzyl alcohol (**10**) in the presence of trifluoromethanesulfonic anhydride. Subsequent in situ one-pot deprotection of the Boc groups with HCl yielded (*S,S*)-**11** in 43% yield and >20:1 *d.r.* (determined by ^1^H NMR) (Scheme 1e). Basic hydrolysis with NaOH finally yielded (*S,S*)-**1** in 48% yield (Scheme 1f).

**Scheme 1:**
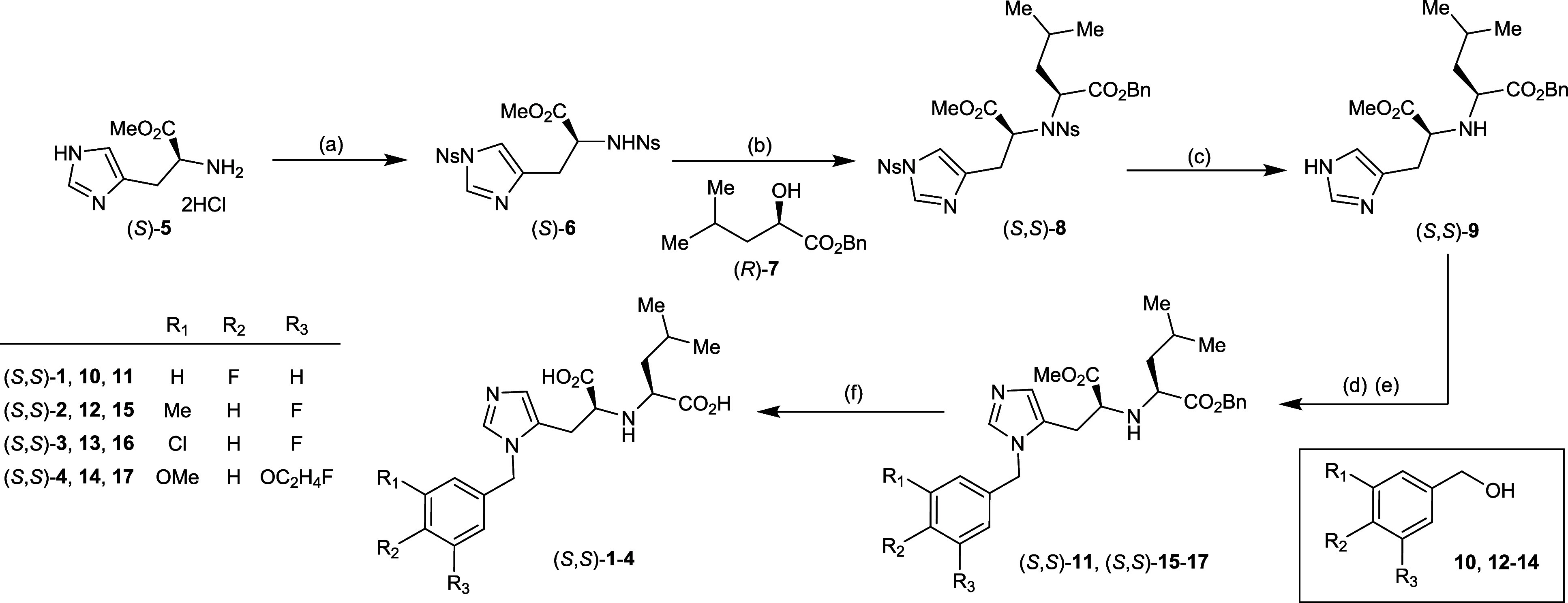
Synthesis of Fluorinated MLN-4760 Analogues (*S*,*S*)-**1**–(*S*,*S*)-**4** ^a^

a Reagents and conditions: (a) Et_3_N (3.0 equiv), CH_2_Cl_2_, rt, 1 h, then NsCl (2.2 equiv), rt, 18 h, 76%; (b) PPh_3_ (1.0 equiv), DIAD (1.0 equiv), THF, −10 °C, 1 h, then (*S*)-**6** (1.5 equiv), THF, rt, 2 h, then (*R*)-**7** (1.0 equiv), THF, rt, 16 h, 55%, >20:1 d.r.; (c) PhSH (2.2 equiv), K_2_CO_3_ (3.0 equiv), DMF, rt, 1 h, 70%, >20:1 d.r.; (d) (*S,S*)-**9** (1.1 equiv), Boc_2_O (2.2 equiv), DIPEA (2.0 equiv), CH_2_Cl_2_, rt, 24 h, isolated as crude, then (e) (CF_3_SO_2_)_2_O (1.0 equiv), **10**, **12–14** (1.0 equiv), DIPEA (1.5 equiv), −78 °C to rt, 24 h, then HCl (4.0 M in dioxane, 1.1 equiv), rt, 1 h, (*S,S*)-**11** (43%, >20:1 *d*.*r*.), (*S,S*)-**15** (47%, >20:1 *d*.*r*.), (*S,S*)-**16** (46%, >20:1 *d*.*r*.), (*S,S*)-**17** (41%, >20:1 *d*.*r*.); (f) NaOH (3.0 equiv), MeOH/H_2_O, rt, 1 h, work up with HCl (1 M), rt, 30 min, then preparative HPLC purification, (*S,S*)-**1** (48%), (*S,S*)-**2** (44%), (*S,S*)-**3** (44%), (*S,S*)-**4** (49%).

**Table 1:**
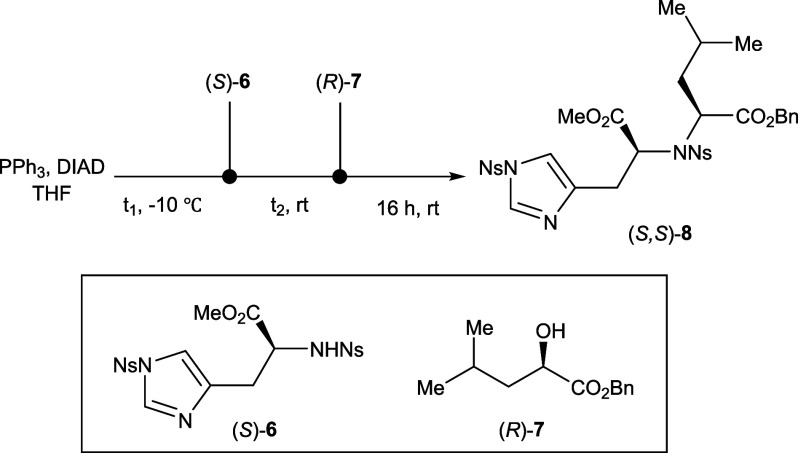
Optimization of the Fukuyama–Mitsunobu Amination^a^

**entry**	**(** *S* **)-6 loading**	***t***_**1**_ **/ h**	***t***_**2**_ **/ h**	**yield of (** *S* **,** *S* **)-8^b^**
1	1.0 equiv	0	0	traces
2	1.0 equiv	0	1	12%
3	1.0 equiv	0	2	21%
4	1.0 equiv	1	2	35%
5	1.5 equiv	0	2	42%
6	1.5 equiv	1	2	55%

a PPh_3_ = 1.0 equiv, DIAD = 1.0 equiv, (*R*)-**7** = 1.0 equiv.

b Yields determined by quantitative ^19^F NMR spectroscopy with 4-fluoroanisole as internal standard.

To confirm the configuration of the product obtained via this new synthetic route, we compared NMR spectroscopic data for ^1^H, ^19^F and ^13^C nuclei and HPLC retention time for (*S,S*)-**1** with previously synthesized (*S,S*)-**1** (*vide supra*).^8^ These data were contrasted to that of diastereomer (*S*,*R*)-**1**, which was prepared applying the same route as (*S,S*)-**1**, using (*S*)-**7** in place of (*R*)-**7** in the Fukuyama-Mitsunobu amination. This allowed for verification of the structure and stereochemistry of (*S,S*)-**1** (Figures S2.1–S2.5). The synthesis of MLN-4760 analogues (*S,S*)-**2–**(*S,S*)-**4** was achieved using this new synthetic route (Scheme 1). From common intermediate (*S,S*)-**9**, the tandem di-Boc-protection-benzylation step was carried out with fluorinated benzyl alcohols **12–14**, providing intermediates (*S,S*)-**15–**(*S,S*)-**17** in moderate yields (41–47%, >20:1 *d.r*, determined by ^1^H NMR). The final basic hydrolysis step (*vide supra*) yielded the desired analogues (*S,S*)-**2–**(*S,S*)-**4** as pure diastereomers with yields ranging from 44–49%.

With PET imaging in mind, this synthetic route was also applied to the preparation of model pinacol boronic ester (*S*,*S*)-**18** that we surmised could be a suitable precursor to (*S*,*S*)-[^18^F]**1** applying copper-mediated radiofluorination.^14^ Starting from intermediate (*S*,*S*)-**9**, N3-selective benzylation with 4-bromobenzyl alcohol and subsequent Miyaura borylation provided (*S*,*S*)-**18** (13% over two steps, >20:1 *d.r*.) (Scheme S2.4). Applying the protocol established in our laboratory for the copper-mediated ^18^F-fluorination of aryl pinacol boronic esters,^14d,14^ (*S*,*S*)-[^18^F]**11** was successfully obtained in radiochemical yield (RCY) of 24% ± 7% (*n* = 2), as determined by radio-HPLC analysis of the crude reaction (Scheme 2A (i)). Subsequent basic hydrolysis of the ester protecting groups resulted in full conversion of (*S*,*S*)-[^18^F]**11**, and formation of (*S*,*S*)-[^18^F]**1** (average RCY = 18%, over two steps) (Scheme 2A (ii)). The pinacol boronic ester precursor to (*S*,*S*)-[^18^F]**3** was prepared analogously ((*S*,*S*)-**19**, Scheme S2.5). Subjecting this material to copper-mediated ^18^F-fluorination followed by in situ deprotection furnished (*S*,*S*)-[^18^F]**3** in 8% RCY over two steps (Scheme 2B).

**Scheme 2:**
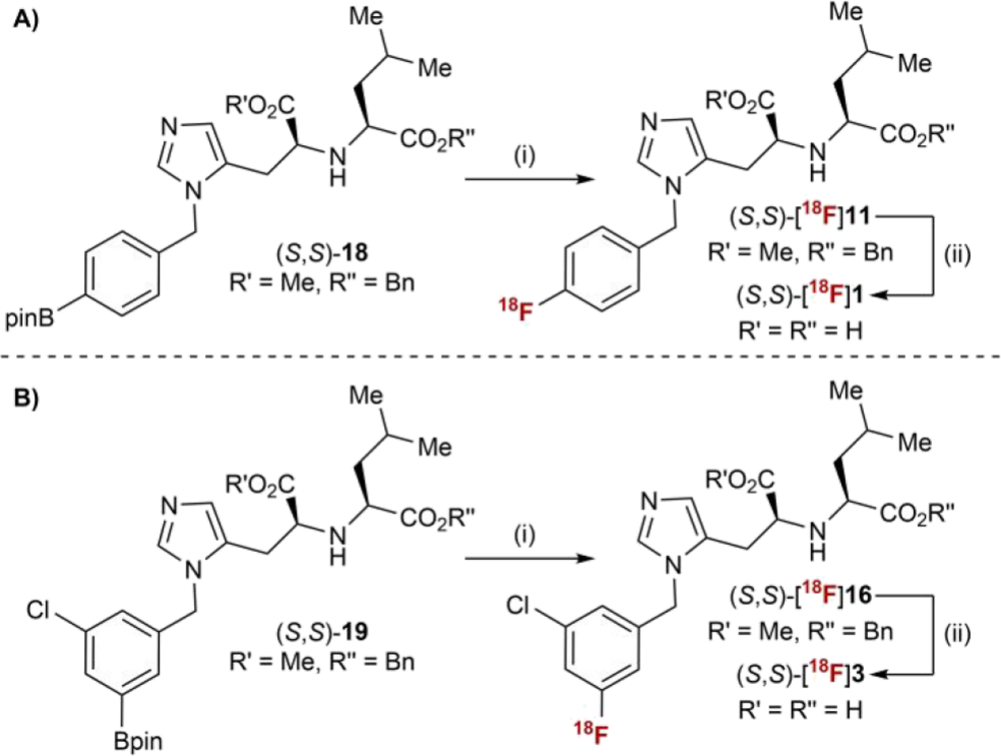
Radiolabeling of MLN-4760 Analogues (A) Radiosynthesis of (*S*,*S*)-[^**18**^F]**1**; (B) Radiosynthesis of (*S*,*S*)-[^**18**^F]**3** ^a^

a Radiochemical yields (RCY) determined by radio-HPLC analysis of crude reaction mixtures. Reagents and conditions: (i) (*S*,*S*)-**18** (5 μmol) or (*S*,*S*)-**19** (10 μmol), [^18^F]­KF.K_222_ (5–20 MBq), Cu­(OTf)_2_py_4_ (2 equiv), DMI (0.3 mL), purged with air (20 mL), then 120 °C, 20 min; RCY [(*S*,*S*)- [^18^F]**11**] = 24% ± 7% (*n* = 2), RCY [(*S*,*S*)- [^18^F]**16**] = 9% ± 3% (*n* = 2) (ii) NaOH (aq., 1 M, 0.1 mL), 65 °C, 20 min, then HCl (aq., 1 M, 0.1 mL); RCY [(*S*,*S*)- [^18^F]**1**] (over two steps) = 18% ± 8% (*n* = 2). RCY [(*S*,*S*)-[^18^F]**3**] (over two steps) = 8% ± 3% (*n* = 2).

Having validated our radiosynthetic approach to (*S*,*S*)-[^18^F]**1** and with various fluorinated MLN-4760 analogues in hand, we next looked to evaluate the inhibitory activity of the analogues using a commercially available ACE2 inhibition assay kit (Table 2) (Figure 3). Due to differences in assay conditions compared to those used in the literature, the pIC_50_ of MLN-4760 was first remeasured, yielding a lower experimental pIC_50_ (pIC^exp^_50_) value of 8.19 compared to the reported literature value of 9.36.^8^ Consequently, the pIC_50_ values of the tested compounds were evaluated relative to our experimentally measured value for MLN-4760 in order to identify lead candidates for radiolabeling. In line with observations made for MLN-4760,^8^ the stereochemistry of the compounds significantly affected their inhibitory effects; compound (*S,S*)-**1** (pIC^exp^_50_ = 6.69 ± 0.05) was indeed markedly more potent than (*S,R*)-**1** (pIC^exp^_50_ = 4.39 ± 0.04) with a difference of 2 orders of magnitude, suggesting configuration-dependent binding between ACE2 and MLN-4760 analogues.^8^ 3,5-Disubstituted benzyl compounds (*S,S*)-**2**, (*S,S*)-**3**, and (*S,S*)-**4** had pIC_50_ values similar to MLN-4760 (pIC^exp^_50_ = 7.54 ± 0.06) when subjected to the assay, with (*S,S*)-**3** (pIC^exp^_50_ = 7.61 ± 0.09) being the most effective ACE2 inhibitor compared to *para*-substituted (*S,S*)-**1**. The difference between the in vitro pIC^exp^_50_ for MLN-4760 and the pIC^exp^_50_ values of (*S,S*)-**1–**(*S,S*)-**4** aligned with the difference in the *in silico* predicted pIC_50_ (pIC^pr^_50_) of MLN-4760 and pIC^pr^_50_ values of (*S,S*)-**1–**(*S,S*)-**4**. Specifically, these pIC_50_ differences for all 3,5-disubstituted benzyl analogues (*S,S*)-**2**, (*S,S*)-**3**, and (*S,S*)-**4** were smaller than for the *para*-substituted benzyl analogue (*S,S*)-**1**, providing support for the pIC^pr^_50_ values (Table 2). Consequently, compounds (*S,S*)-**2**, (*S,S*)-**3** and (*S,S*)-**4**, with pIC_50_ values in the same range as MLN-4760, emerge as priority targets for future ACE2 PET radioligand development.^15^

**Table 2:** In Vitro ACE2 Inhibition Study for MLN-4760 Analogues ((*S*,*S*)-**1**–(*S*,*S*)-**4**) and Comparison to *In Silico* Predictions^a^

compound	pIC^pr^_50_	pIC^pr‑MLN^_50_ – pIC^pr^_50_	pIC^exp^_50_	pIC^exp‑MLN^_50_ – pIC^exp^_50_
MLN-4760	8.19	0.00	7.54 ± 0.06	0.00
(*S*,*S*)-**1**	7.14	1.05	6.69 ± 0.05	0.85
(*S*,*R*)-**1**	n.d.i	n.d.	4.39 ± 0.04	3.15
(*S*,*S*)-**2**	7.94	0.25	7.18 ± 0.04	0.36
(*S*,*S*)-**3**	7.99	0.20	7.61 ± 0.09	–0.07
(*S*,*S*)-**4**	8.88	–0.69	7.27 ± 0.05	0.27

a pIC_50_ determined using Abcam ACE2 inhibitor screening kit. n.d., not determined. pIC^pr^_50_, predicted pIC_50_, pIC^pr‑MLN^_50_, predicted MLN-4760 pIC_50_, pIC^exp^_50_, experimental pIC_50_, pIC^exp‑MLN^_50_, experimental MLN-4760 pIC_50_.

**Figure 3:**
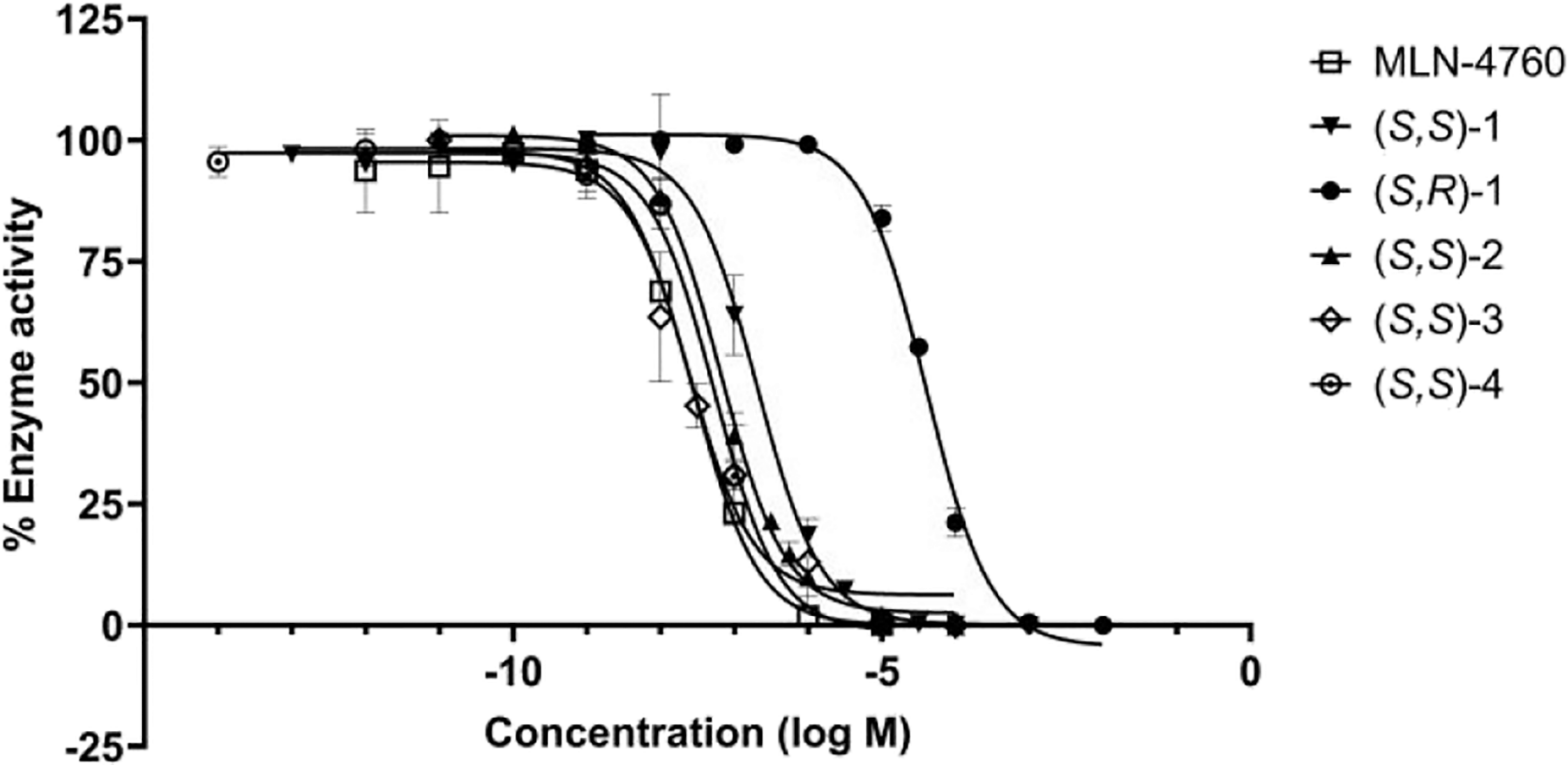
Inhibitory activity of MLN-4760 and analogues (*S*,*S*)-**1**–(*S*,*S*)-**4**, (*S*,*R*)-**1**.

This work aimed toward the discovery of highly potent and selective candidates for ACE2 PET imaging - a biomarker implicated in various diseases, including COVID-19. First, *in silico* screening of a panel of analogues of lead compound MLN-4760 identified promising radiotracer candidates. This was followed by the development of a novel synthetic approach to these MLN-4760 analogues using *para*-fluorobenzyl containing model target (*S,S*)-**1** and leveraging the Fukuyama-Mitsunobu reaction and late-stage benzylation as key steps. This approach offered improved yield and control over diastereoselectivity compared to existing synthetic routes, accelerating the synthesis of four fluorinated MLN-4760 analogues. The same route was applied to the preparation of model pinacol boronic ester precursor (*S*,*S*)-**18**, which was converted into (*S*,*S*)-[^18^F]**1** upon copper-mediated radiofluorination. (*S*,*S*)-[^18^F]**3** was prepared in the same way. Finally, evaluation of the potency of our collection of fluorinated MLN-4760 analogues via in vitro IC_50_ assays confirmed their ACE2 inhibitory activity. Compounds (*S*,*S*)-**2**, (*S,S*)-**3** and (*S*,*S*)-**4** stand out as prime candidates for future PET imaging studies.^15^ Further investigation into their radiosynthesis and evaluation as ACE2 PET radiotracers is ongoing in our laboratories.

## Supplementary Material



## Data Availability

The data underlying this study are available in the published article and its Supporting Information.

## Notes

Computational data and procedures; preparation and characterization of products and intermediates; radiochemistry data and protocols; in vitro study data and protocols; and NMR spectra for novel compounds (PDF)
