# Supplementary material for: Synthesis and Inhibitory Assessment of ACE2 Inhibitors for SARS-CoV-2: An In Silico and In Vitro Study
Source: J Org Chem. 2025 Jul 21;90(30):10941–7. doi: 10.1021/acs.joc.5c00918 (PMC12322948; doi:10.1021/acs.joc.5c00918)

Supplementary Information

Synthesis and Inhibitory Assessment of ACE2 Inhibitors for SARS-CoV-2: An *In Silico* and *In Vitro* Study

Xiaoyun Wang[†], Jieyu He[‡], Layla Hosseini-Gerami[¶], Morgan Thomas[¶], Stephen Thompson[‡], Joseph Ford[†], Sebastiano Ortalli[†], Zijun Chen[†], Gianluca Destro[†], Andreas Bender[¶], Franklin Aigbirhio[‡,*], Véronique Gouverneur[†,*]

[†]Chemistry Research Laboratory, University of Oxford, 12 Mansfield Road, Oxford, OX1 3TA, United Kingdom.

[‡]Molecular Imaging Chemistry Laboratory, Wolfson Brain Imaging Centre, Department of Clinical Neurosciences, University of Cambridge, Cambridge CB2 0QQ, United Kingdom.

[¶]Centre for Molecular Informatics, Department of Chemistry, University of Cambridge, Cambridge, CB2 1EW, United Kingdom.

Contents

1. In silico prioritization of MLN-4760 analogues S3
1.1 Protein and ligand preparation S3
1.2 Validation of computational docking for ACE2 ..... S3
1.3 Estimation of analogue binding affinity by docking S5
1.4 Validation of molecular dynamics for ACE2 ..... S7
1.5 Relative binding free energy protocol S7
1.6 Validation of relative binding free energy for ACE2 ..... S9
1.7 Estimation of radiolabelled analogue binding affinity by RBFE S10
2. Synthesis S12
2.1 General Information S12
2.2 Preliminary synthetic work S13
2.2.1 Initial synthetic route S13
2.2.2. Optimization of the reductive amination step S14
2.2 Fukuyama-Mitsunobu amination S15
2.3 Comparison of two diastereomers S17
2.3.1 NMR spectroscopy analysis S17
2.3.2 HPLC analysis S19
2.4 Synthetic protocols and characterization S21
3. Radiochemistry S53
3.1 General Information S53
3.2 Radiochemistry protocols S54
4. ACE2 Inhibition Assay S61
5. References S63
6. NMR Spectra for Novel Compounds S65

1. In silico prioritization of MLN-4760 analogues

1.1 Protein and ligand preparation

An X-ray structure of MLN-4760 co-crystallised with ACE2 (PDB: 1R4L) was utilized as the model system in this work.[1] The complex was prepared with Maestro's (Schrödinger, LLC, New York, NY) Protein Preparation Wizard. Four missing side chain residues were rebuilt with Prime, H-bonds were assigned and their orientations optimized using PROPKA,[2] finally the protein-ligand complex was minimized with the default heavy atom constraint of 0.3 Å as per default in Protein Preparation Wizard. This complex was used to build the receptor grid for docking that was centred on the ligand using default parameters. All molecules docked against the system were prepared and embedded into 3D from SMILES using LigPrep (Schrödinger, LLC, New York, NY), including assignment of charges at pH 7 ± 2.

1.2 Validation of computational docking for ACE2

First, MLN-4760 was re-docked into its original X-ray co-crystal structure with ACE2 to provide a sanity check that computational docking correctly predicted the bioactive conformation of MLN-4760.[1] MLN-4760 was prepared from SMILES and then re-docked using the prepared grid, using GlideXP without nitrogen inversion or ring conformation sampling, which resulted in the most accurate pose. The top-ranked docked pose identified the correct bioactive conformation with an RMSD of 1.88 Å to the co-crystallised MLN-4760 compound.

Following the validation of accurate bioactive conformation prediction of MLN-4760, known analogues published in the original report were docked using the same protocol (Figure S1.1).[3] The GlideXP docking score, and other calculated properties were compared to their published ACE2 binding affinity. The strongest correlation identified was between the $Glide_{evdw}$, or Van der Waals energy, which showed a Pearson correlation of -0.76 and Spearman rank correlation of -0.83 with compound pIC_{50} values (Figure S1.2). A simple linear model was fit between experimental pIC_{50} and $Glide_{evdw}$ (Equation S1) with $\beta = -2.12$ and $\alpha = -14.77$. An interpolation of the fitted data using this equation is shown in Table S1.1.

$$pIC_{50} = \beta \times Glide_{evdw} + \alpha \qquad (Equation\ S1)$$

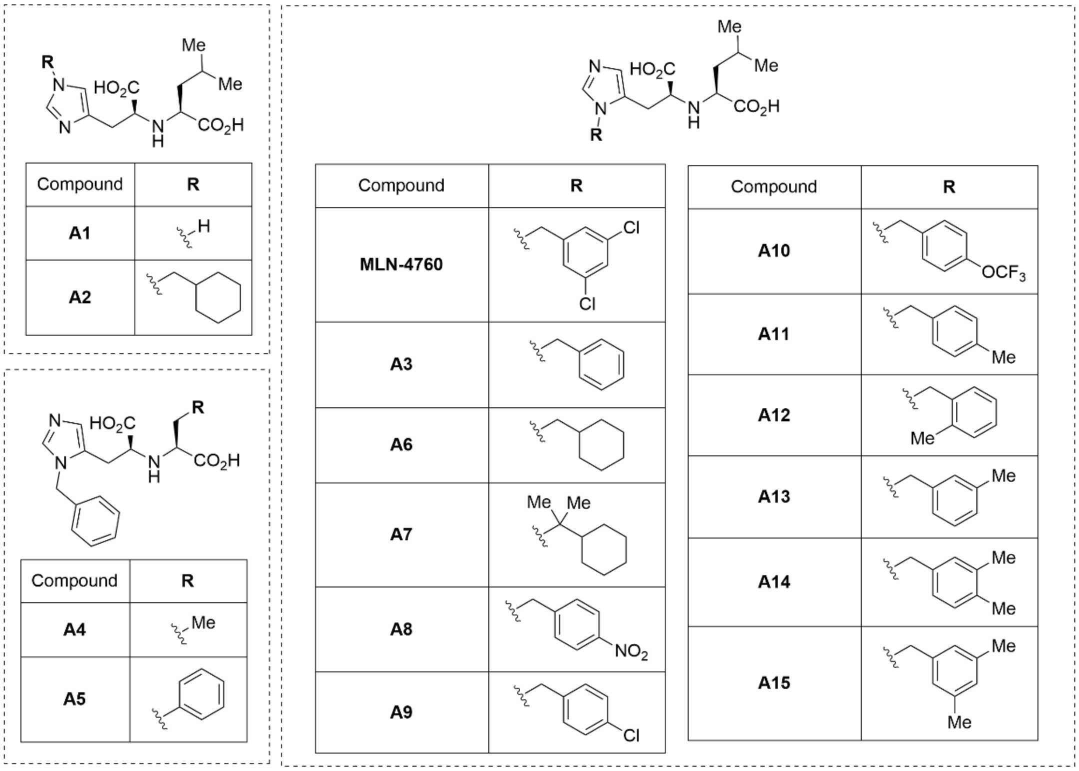

Figure S1.1. Structures of MLN-4760 and analogues thereof reported by Dales *et al*.

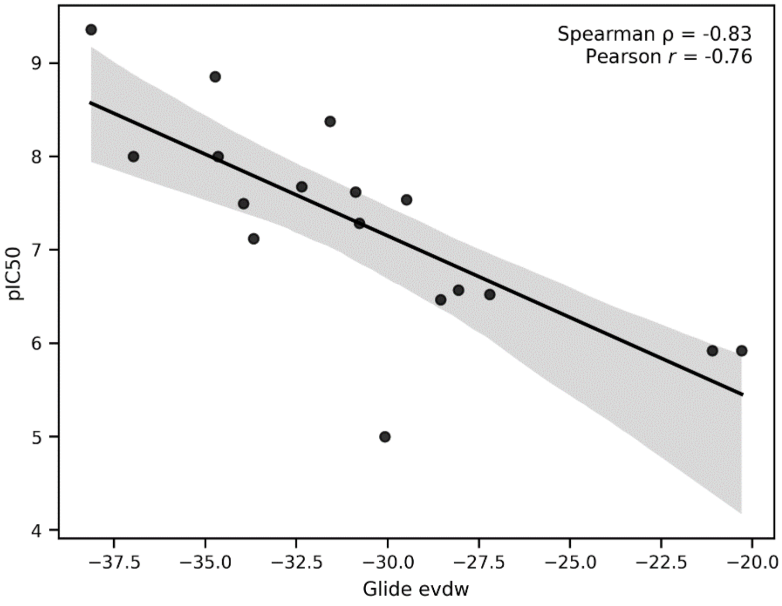

Figure S2.2. Scatterplot showing experimental pIC_{50} vs calculated evdw of MLN-4760 and known analogues.

Table S1.1 Retrospectively modelled analogues of MLN-4760 including reported pIC_{50} and interpolated estimated pIC_{50} using the linear model of $Glide_{evdw}$ energy.

Compound	Reported pIC_{50}	Estimated pIC_{50}	Error
MLN-4760	9.36	8.19	1.17
A4	6.52	7.40	0.87
A8	7.12	7.46	0.34
A9	7.68	7.92	0.24
A10	7.28	6.84	0.44
A11	7.49	8.00	0.51
A12	7.54	7.48	0.06
A13	8.38	7.94	0.44
A14	8.00	8.73	0.73
A15	8.85	8.26	0.59

1.3 Estimation of analogue binding affinity by docking

A selection of 31 possible analogues of MLN-4760 (Figure S1.3) were docked using the same protocol as previously described. The $Glide_{evdw}$ of the top-ranked pose and the linear model fit on known analogue pIC_{50} values was used to estimate the pIC_{50} of the analogues. As the radiolabel is assumed to have no effect of protein binding, the effect of this is not modelled.

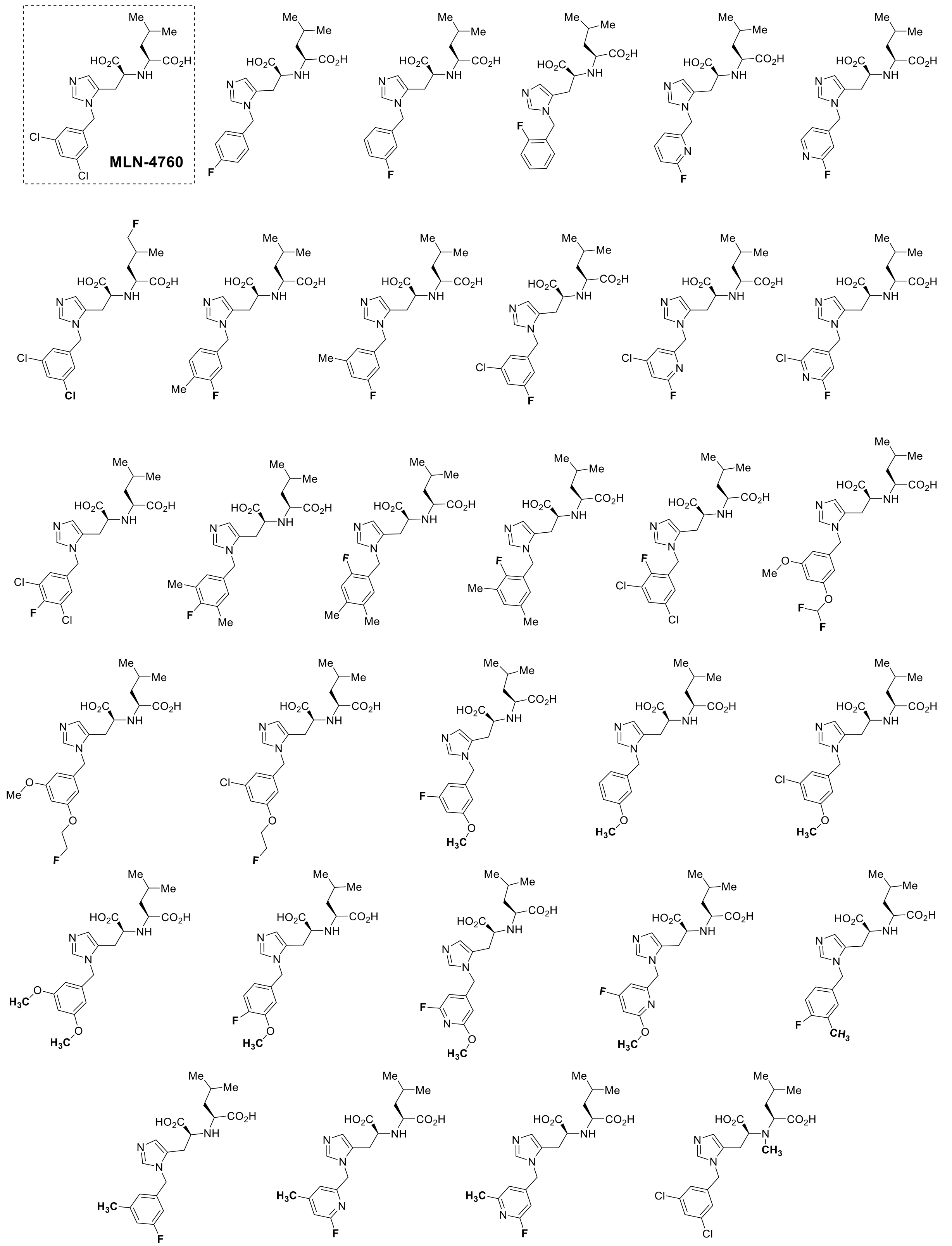

Figure S1.3. Possible radiolabelled analogues of MLN-4760 considered for *in silico* prioritisation.

1.4 Validation of molecular dynamics for ACE2

ACE2 is a challenging system to run relative binding free energy (RBFE) calculations on, due to the binding of the ligand to a coordinated Zn^{2+} ion. This should require bespoke parameterization of the forcefield that was beyond the resources available in this work. However, as the Zn^{2+} site (Figure S1.4, S1) is relatively distant from the proposed analogue changes to the ligand (Figure S1.4, S1'), hence we here assumed that poor force-field parameterisation of the Zn ion should have marginal effects in the calculation. To ensure the simulation was still stable a control simulation was conducted. GROMACS 2020.2 was used as the molecular dynamics (MD) engine.[4] The prepared X-ray crystal structure of ACE2 bound to MLN-4760 (PDB: 1R4L) was taken from the docking protocol.[1] The receptor complex was parameterized using the amber99sb force field with a TIP3P forcefield model for water.[5,6] The ligand complex was parameterized using the GAFF force field *via* antechamber[7,8] and parameter files generated using tleap before using parmed[9] and ProtoCaller[10] to convert the files back into GROMACS. BioSimSpace was then used to merge the receptor and ligand into a complex and then solvate a box of 90$Å^3$.[11] The complex was energy minimized for 50,000 steps before undergoing equilibration in NVT and then NPT conditions for 100 ps each. Then with the system equilibrated, production simulation was run for 50 ns with a timestep of 2 fs. Over the course of the simulation the zinc ion did not move more than 2 Å relative to the protein backbone. Concluding that the force field would be sufficient for the complex to remain stable over the timescale of the RBFE simulations used in this work of 1 ns (1/50th of the control simulation).

1.5 Relative binding free energy protocol

RBFE simulations were run following the ProtoCaller default protocol with GROMACS 2020.2 as the MD engine.[10] The same receptor parameterization and files were used as for the MD control. Ligand molecules were edited directly from the MLN-4760 reference ligand in PyMol, any sub-optimal geometries are minimized during energy minimizing before MD simulation. Similarly, the ligand molecules were parameterized as in the molecular dynamics control. Then, provided the ligand structures, ProtoCaller was used to generate morph structure and topology files. Following the suggested protocol on GitHub,[12] 40 λ windows were used to scale Lennard-Jones, bonded and Coulomb values. Each λ window consisted of energy minimization for 25,000 steps, NVT and

NPT equilibration for 50 ps respectively, followed by a final production simulation for 1 ns. For further information, the reader is referred to Suruzhon *et al.*[10] Note that non-symmetric aryl analogues with ambiguous orientation in the S1' site were run in both the 'up' (towards Asp368) and 'down' orientation, and the result averaged (Figure S1.4).

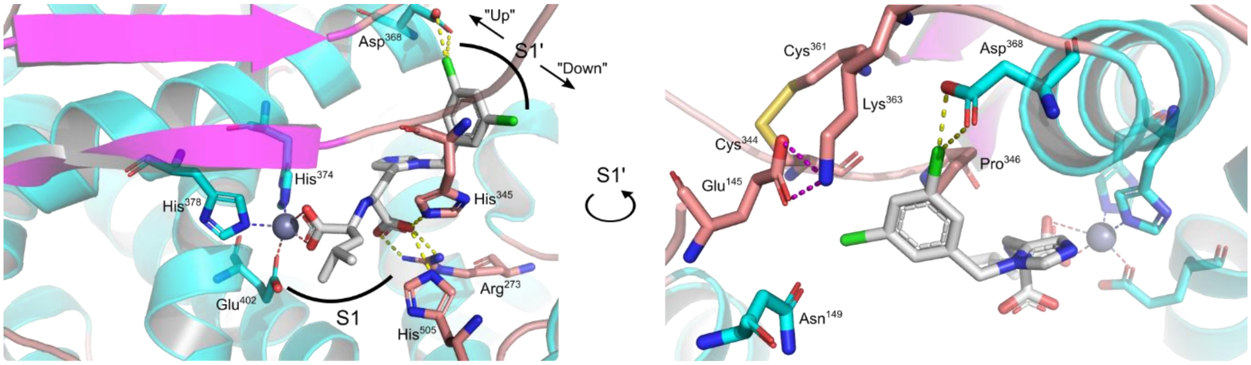

Figure S1.4. X-ray crystal structure of ACE2 bound to inhibitor MLN-4760 (PDB: 1R4L). Sub pockets S_1 and S_1' are annotated alongside orientation annotation used in this work for rotamers of asymmetric substructures in the S_1' sub-pocket. Left: view through the S1 subsite at zinc coordination of MLN-4760. Right: view through the S1' subsite and nearby residues, notably Asp^{368} interacting with the 3,5-dichlorobenzyl moiety.

To calculate relative change in free energy, the alchemical-analysis package[13] was utilised to run MBAR estimations of the energy difference states.[14] Convergence of the free energy was visually assessed in both the forward and reverse directions. This was corrected using a simple linear model between real pIC_{50} and predicted pIC_{50} for analogue compounds. The final absolute predicted pIC_{50} has then been taken as the difference between the reference compound and the predicted, corrected change in free energy ($\Delta\Delta G^*$) (Equation S2). This is approximately equal to pIC_{50} because we are neglecting the small difference between binding free energy and binding constants which are typically calculated in binding assays, not withholding expected error associated with the assay.

$$\Delta\Delta G = \Delta G_{bound} - \Delta G_{unbound}$$
$$\Delta\Delta G^* = \beta \times \Delta\Delta G + \alpha \qquad (Equation\ S2)$$
$$pIC_{50} \cong pIC_{50\,ref} - \Delta\Delta G^*$$

An error value is calculated for the prediction. First the error calculated by MBAR for the bound and unbound legs is summed ($\Delta\Delta G_{error}$). This is then propagated with the errors in the linear model (Equation S3).

$$pIC_{50error} = pIC_{50} \times \sqrt[2]{\left(\frac{\beta_{error}}{\beta}\right)^2 + \left(\frac{\Delta\Delta G_{error}}{\Delta\Delta G}\right)^2} + \sqrt[2]{\left(\frac{\alpha_{error}}{\alpha}\right)^2} \qquad (Equation\ S3)$$

1.6 Validation of relative binding free energy for ACE2

RBFE calculations were first validated on a subset of known analogues (Figure S1.5) published alongside MLN-4760, similar to validation of the docking protocol. Due to the increased computational expense of the simulations, a subset of the analogues most similar to MLN-4760 were chosen. Single perturbations between matched molecular pairs of the analogues were run. Most perturbations were predicted within 1 kcal/mol of the real difference in free energy, however, two were notably inaccurate (A8→A3, A10→A3) to the extent of incorrect relative classification i.e., stronger, or weaker binder relative to the reference. Upon further inspection, the two incorrectly classified perturbations and MLN-4760→A9 contained at least one artefact during the simulation of either the first or last λ window, such as, free solvent ion coordination to the binding site or a significant change in binding mode. Due to the limited resources available, simulations with such artefacts were omitted. A simple linear model similar to Equation S1 was fit on the remaining perturbations, resulting in a linear regression fit of α=-0.0552, β=0.4926 with Spearman rank correlation of 0.87 and Pearson correlation of 0.96, albeit from a small dataset. This model was subsequently used as a correction term between the RBFE predicted delta free energy of binding (Pred. ΔΔG) and the experimental (Exp. ΔΔG) assumed from the difference in pIC_{50}.

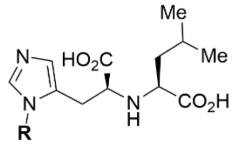

Compound	R	ACE2 IC50 (μM)	ACE IC50 (μM)
**MLN-4760**	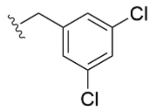	0.00044	>100
**A3**	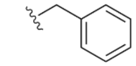	0.024	>100
**A8**	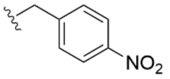	0.076	
**A9**	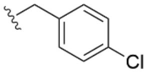	0.021	
**A10**	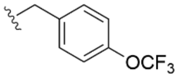	0.052	
**A11**	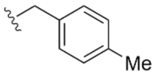	0.032	>10
**A13**	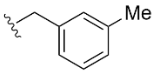	0.0042	>10
**A14**	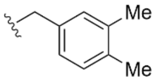	0.010	>10
**A15**	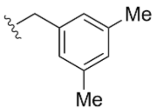	0.0014	>10

Figure S1.5. Analogues to MLN-4760 with reported binding affinity to ACE2 used for RBFE validation studies.

1.7 Estimation of radiolabelled analogue binding affinity by RBFE

A smaller subset of more promising radiolabelled analogues based on the results of docking simulations and estimated ease of synthesis were subject to RBFE simulations. In total, 14 out of the initial 31 compounds were carried forward for more computationally expensive RBFE calculations. Table S1.2 shows the predicted binding affinity of the proposed analogues according to single perturbation simulations from MLN-4760 or its analogues based on minimal alchemical changes. Most corresponded to structural changes in the S1’ sub-pocket of ACE2. In cases where a structural modification resulted in an asymmetric sub-structure, both ‘up’ and ‘down’ orientations in the S1’ sub-pocket was run and averaged if possible.

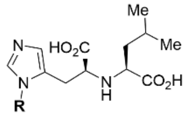

Reference compound	Reference structure (R =)	Proposed structure (R =)	Predicted ΔΔG	Reference pIC_{50}	Predicted pIC_{50}
**A11**	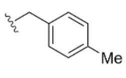	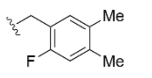	1.484 (± 0.04)	7.49	6.81 (± 1.39)
**A15**	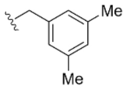	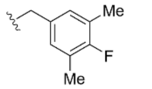	-0.393 (± 0.02)	8.85	9.10 (± 1.30)
**MLN-4760**	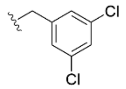	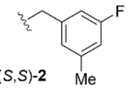 (*S*,*S*)-**2**	1.958 (± 0.04)	9.36	8.45 (± 1.41)
**MLN-4760**	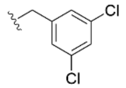	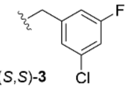 (*S*,*S*)-**3**	1.497 (± 0.03)	9.36	8.68 (± 1.39)
**MLN-4760**	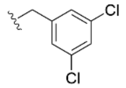	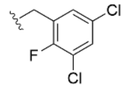	2.387 (± 0.02)	9.36	8.83 (± 1.37)
**MLN-4760**	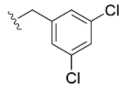	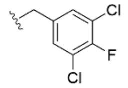	0.268 (± 0.02)	9.36	9.28 (± 1.33)
**MLN-4760**	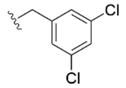	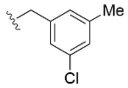	1.285 (± 0.02)	9.36	8.78 (± 1.38)
**MLN-4760**	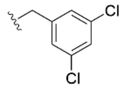	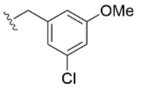	1.203 (± 0.04)	9.36	8.71 (± 1.26)
**MLN-4760**	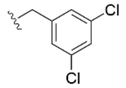	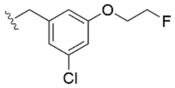	-0.550 (± 0.06)	9.36	9.58 (± 1.35)
**MLN-4760**	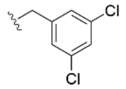	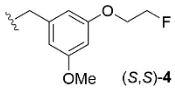 (*S*,*S*)-**4**	-0.566 (± 0.04)	9.36	9.58 (± 1.36)
**A11**	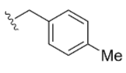	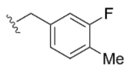	0.001 (± 0.02)	7.49	7.54 (± 1.32)
**A15**	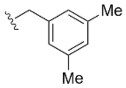	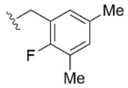	1.698 (± 0.02)	8.85	8.49 (± 1.36)
**A9**	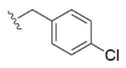	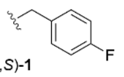 (*S*,*S*)-**1**	0.364 (± 0.03)	7.68	7.56 (± 1.34)

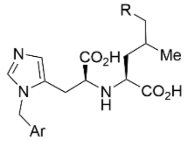

Reference compound	Reference structure (R =)	Proposed structure (R =)	Predicted ΔΔG	Reference pIC_{50}	Predicted pIC_{50}
**MLN-4760**	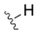	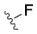	0.631 (± 0.02)	9.36	9.10 (± 1.35)

Table S1.2. Predicted differences in binding energy ($\Delta\Delta G$) and pIC50 values for radiotracer candidates determined by RBFE simulations.

2. Synthesis

2.1 General Information

Starting materials ((*S)*-histidine methyl ester dihydrochloride ((*S*)-**5**), (4-fluorophenyl)methanol, (*R*)-2-hydroxy-4-methylpentanoic acid, *(S)*-2-hydroxy-4-methylpentanoic acid, (3-fluoro-5-methylphenyl)methanol, (3-chloro-5-fluorophenyl)methanol and methyl 3,5-dihydroxybenzoate) and reagents were obtained from commercial suppliers (Sigma-Aldrich, Alfa Aesar, Fluorochem, Apollo Scientific) and were used as received without further purification. For reactions to be carried out under inert conditions, reaction flasks and magnetic stirrers were flame-dried under vacuum and refilled with nitrogen. Dry solvents were obtained from commercial suppliers and dried using an MBRAUN-SPS solvent purification system. Reaction progress was tracked via thin-layer chromatography (TLC) using silica gel-coated aluminum sheets (Merck Kieselgel 60 F_{254} plates). TLC plates were visualized using ultraviolet light at 254 nm, and using ninhydrin as a stain for amino acid derivatives. Flash column chromatography was conducted with Merck silica gel (60, particle size 0.040-0.063 mm). Nuclear magnetic resonance (NMR) analyses were performed at 298 K, unless otherwise specifie, using Bruker AVIIIHD 400, AVIIIHD 500, AVII 500 and NEO 600 spectrometers, and the data were processed using MestreNova 14.0 software or higher. Chemical shifts (δ) for ^{1}H, ^{19}F and ^{13}C NMR spectral data are reported in parts per million (ppm), referenced to the solvent peak using Bruker's internal referencing procedure (edlock), with ^{19}F NMR chemical shifts externally referenced to $CFCl_3$. Coupling constants (*J*) are reported in Hertz (Hz) to the nearest 0.1 Hz. ^{13}C spectra are recorded with proton decoupling, and the reported coupling constants correspond to ^{19}F–^{13}C heteronuclear coupling, unless stated otherwise. Peak multiplicities are described as s (singlet), d (doublet), t (triplet), q (quartet), pent (pentet), sept (septet), br (broad), or m (multiplet). High-resolution mass spectra (HRMS, m/z) were obtained using a Thermo Exactive mass spectrometer combined with a Waters Acquity liquid chromatography system, utilising either the heated electrospray (HESI-II) probe for positive electrospray ionization (ESI+) or an atmospheric pressure chemical ionization (APCI) probe. Infrared spectra were taken either of neat samples or an evaporated solution (thin layer film) using a Bruker Tensor 27 FT-IR spectrometer, with absorptions reported in wavenumbers (cm^{-1}). Melting points for solid compounds were obtained using a Griffin apparatus and are uncorrected. Optical rotations were measured with a Schmidt-Haensch Autopol L 2000 polarimeter at 25 °C,

with measurements at λ_{max} = 589 nm recorded as $[\alpha]_D^{25\,°C}$, with the concentration (in units of g/100 ml) and solvent specified.

2.2 Preliminary synthetic work

2.2.1 Initial synthetic route

The initial synthesis of the 4-fluorobenzyl analogue 1 was based on the synthetic method originally disclosed by Dales et al. for MLN-4760 (Scheme S2.1).[3] This approach was selected to validate the synthesis route for the analogues.

Scheme S2.1. Initial synthesis of MLN-4760 analogues illustrated with analogue (*S*,*S*)-**1**.

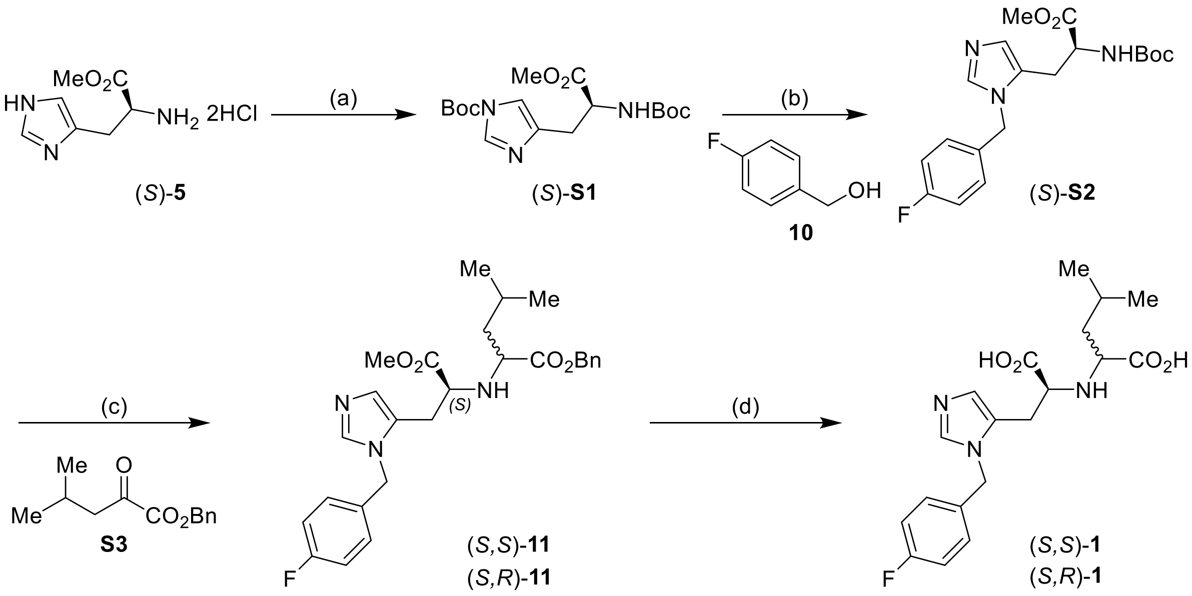

Reagents and conditions: (a) (*S*)-**5** (1.0 equiv.), Boc_2O (2.0 equiv.), Et_3N (2.0 equiv.), MeOH, rt, 16 h, 87%; (b) $(CF_3SO_2)_2O$ (1.0 equiv.), DIPEA (1.2 equiv.), **10** (1.0 equiv.), CH_2Cl_2, -78 °C, 20 min, then (*S*)-**S1** (1.1 equiv.), CH_2Cl_2, rt, 24 h, 78%; (c) (*S*)-**S2** (1.0 equiv.), HCl (4 M in dioxane, excess), 2 h, rt, then Et_3N (2.0 equiv.), CH_2Cl_2, rt, 1 h, then **S3** (1.5 equiv.), AcOH (1.0 equiv.), 4Å MS, rt, 2 h, then $NaB(OAc)_3H$ (3.0 equiv.), CH_2Cl_2, 24 h, 31% over 2 steps, 57:43 d.r.; (d) NaOH (1 M, 3.0 equiv.), MeOH, rt, 1 h, work up with HCl (1 M), rt, 30 min, 35%, 57:43 d.r., after semi-preparative HPLC, 15%.

2.2.2. Optimization of the reductive amination step

In our hands, a low yield was obtained for the key reductive amination step in this original route.[3] This was further optimized to improve the yield of **11**.

Table S2.1. Reaction optimisation of the reductive amination step.

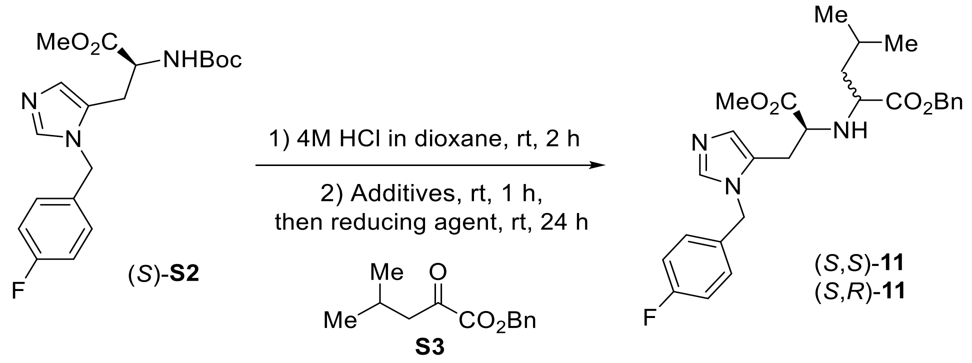

Entry	Solvent	Reducing Agent	Additives	(*S*,*S*)-**11** + (*S*,*R*)-**11** Yield[a]	***d.r.***[b]
**1**[c]	DCE	$NaB(OAc)_3H$	none	10%	55:45
**2**	DCE	$NaBH_4$	none	(n.d.)	N/A
**3**	DCE	$NaBH_3CN$	none	(n.d.)	N/A
**4**	THF	$NaB(OAc)_3H$	none	6%	56:44
**5**	CH_2Cl_2	$NaB(OAc)_3H$	none	12%	56:44
**6**	CH_2Cl_2	$NaB(OAc)_3H$	AcOH + 4Å MS	15%	51:49
**7**	CH_2Cl_2	$NaB(OAc)_3H$	Et_3N[d], then AcOH + 4Å MS	31%	57:43

(*S*)-**S2** (1.0 equiv.), HCl (4 M in dioxane, excess), 2 h, rt, then **S3** (1.5 equiv.), additives (1.0 equiv.), 4Å MS (if used), rt, 2 h, then reducing agent (3.0 equiv.), solvent (0.1 M), 24 h.

[a]Isolated, combined yields of two non-separable diastereomers. [b]Diastereomeric ratio (*d.r.*) determined by ^1H NMR of the mixture. [c]Literature conditions.[3] [d]2.0 equiv. were used. (n.d.) = not determined.

2.2 Fukuyama-Mitsunobu amination

Scheme S2.2 Preliminary attempts for the Fukuyama-Mitsunobu step using different starting materials. Ns = 2-nitrobenzenesulfonyl, DIAD = diisopropyl azodicarboxylate.

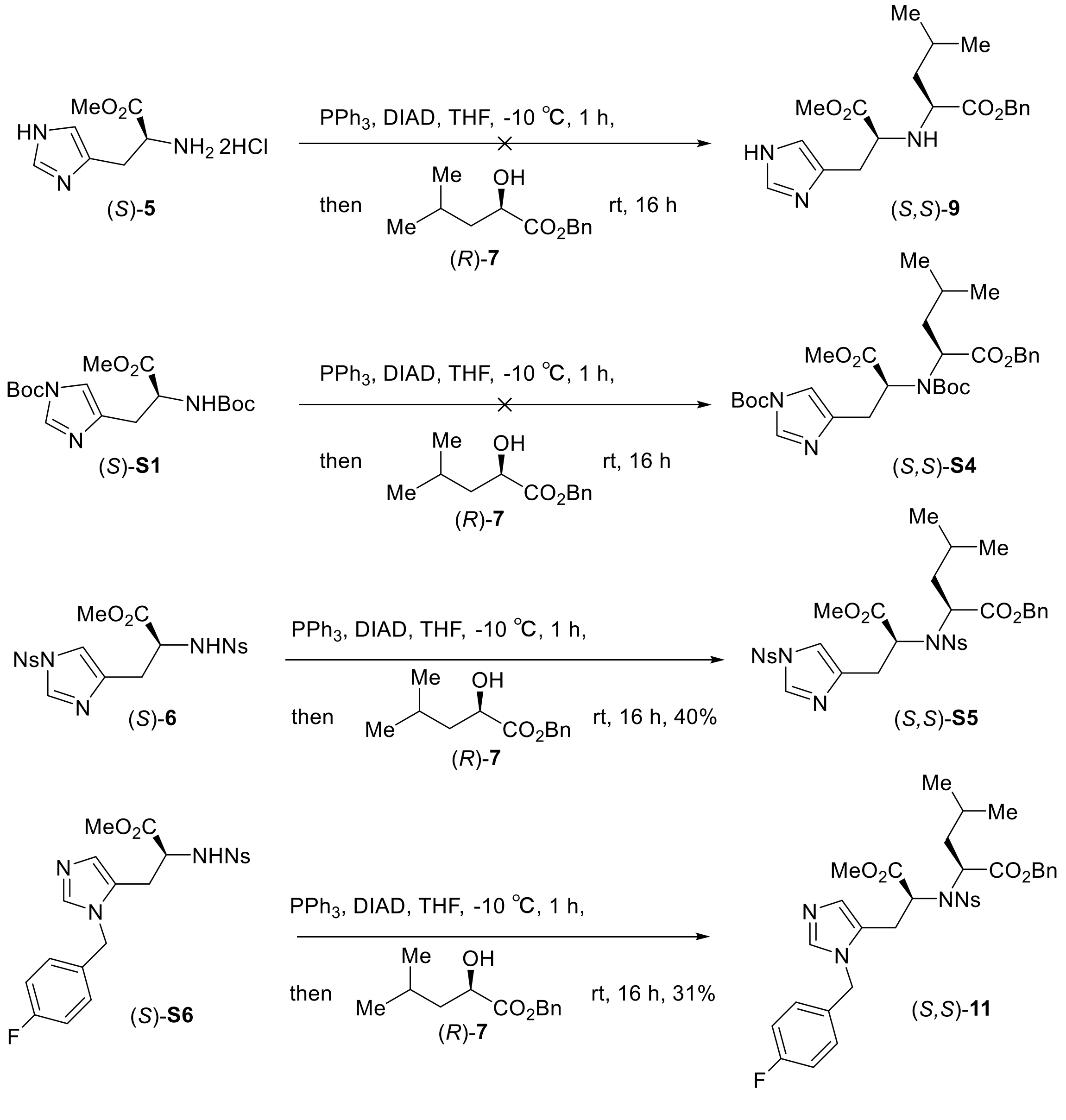

Direct benzylation of the di-Ns protected compound (*S*,*S*)-**8** was unsuccessful; successful benzylation was achieved after protecting group exchange to Boc, yielding the benzylated product (*S*,*S*)-**11**.

Scheme S2.3. Subsequent benzylation of (*S*,*S*)-**8** via a three-step Ns group cleavage-Boc protection-benzylation sequence.

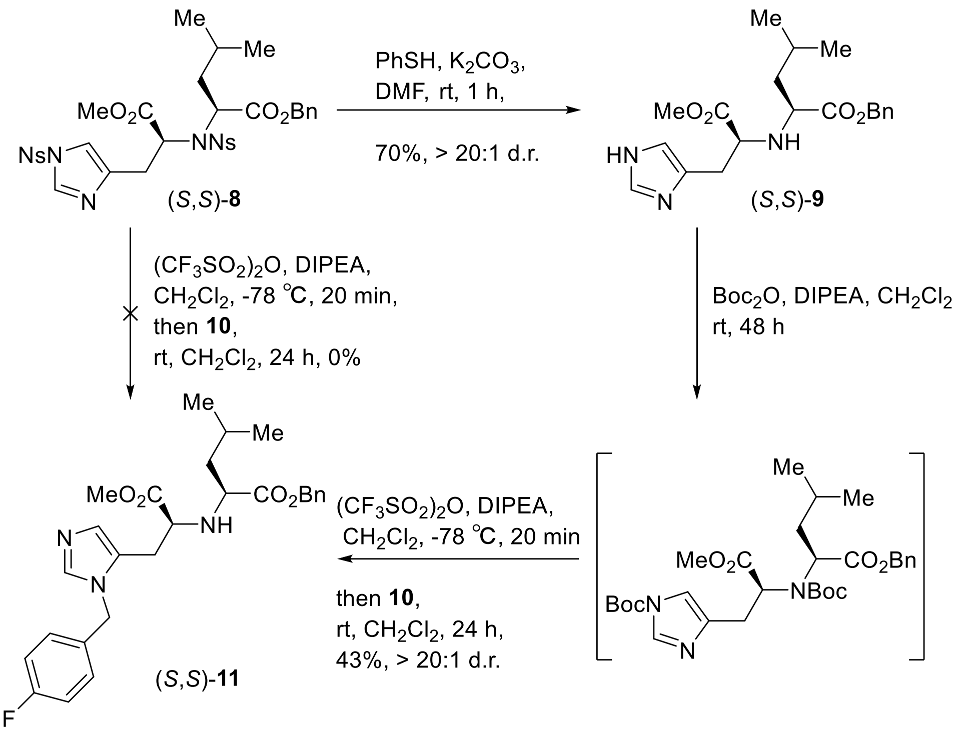

2.3 Comparison of two diastereomers

Confirming the identity of the (*S*,*S*)-**1** and (*S*,*R*)-**1** was achieved employing high-performance liquid chromatography (HPLC) and nuclear magnetic resonance (NMR) spectroscopy.

2.3.1 NMR spectroscopy analysis

The ^{1}H, ^{19}F and ^{13}C NMR spectra of (*S*,*R*)-**1** and (*S*,*S*)-**1** in D_2O indicated the presence of distinct chemical environments between the two compounds, on the basis of differing chemical shifts for key resonances between the two compounds (Figures S2.1-S2.4, highlighted signals).

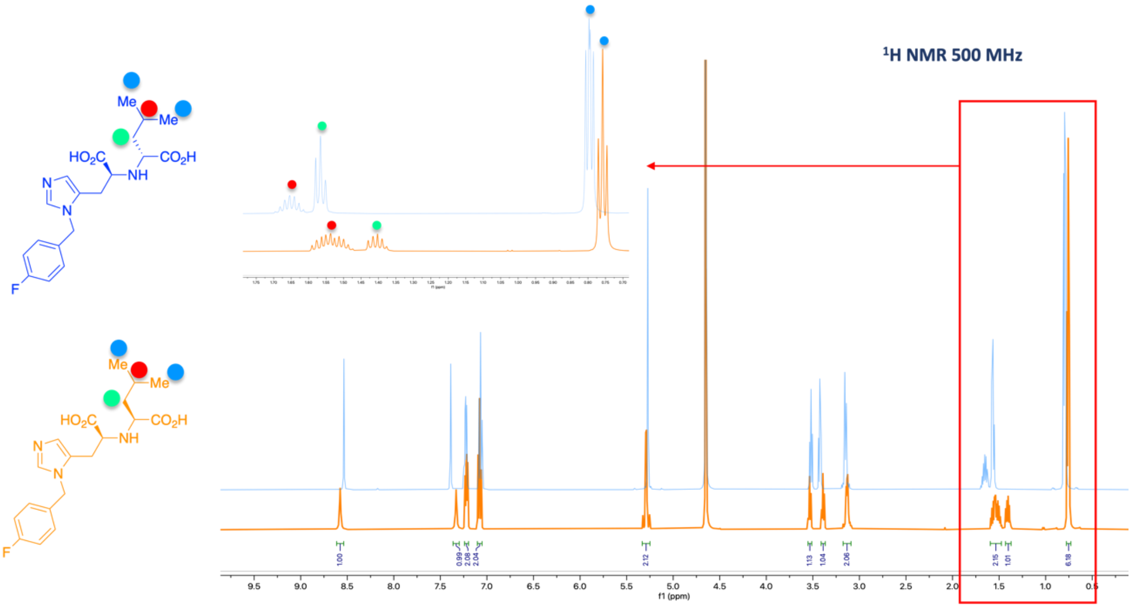

Figure S2.1. Overlapped ^{1}H NMR spectra of (*S*,*R*)-**1** (blue) and (*S*,*S*)-**1** (orange), expanded in the region of 0.5 to 1.8 ppm.

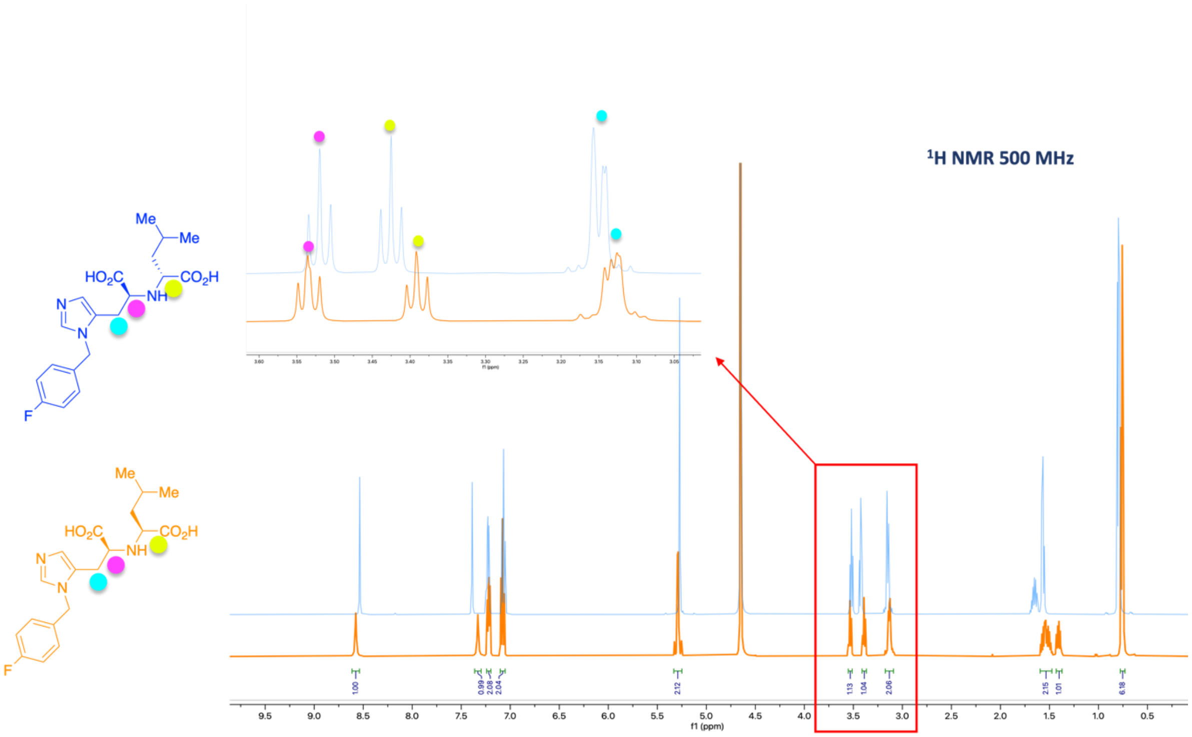

Figure S2.2. Overlapped ^{1}H NMR spectra of (*S*,*R*)-**1** (blue) and (*S*,*S*)-**1** (orange peaks), expanded in the region of 3.0 to 3.6 ppm.

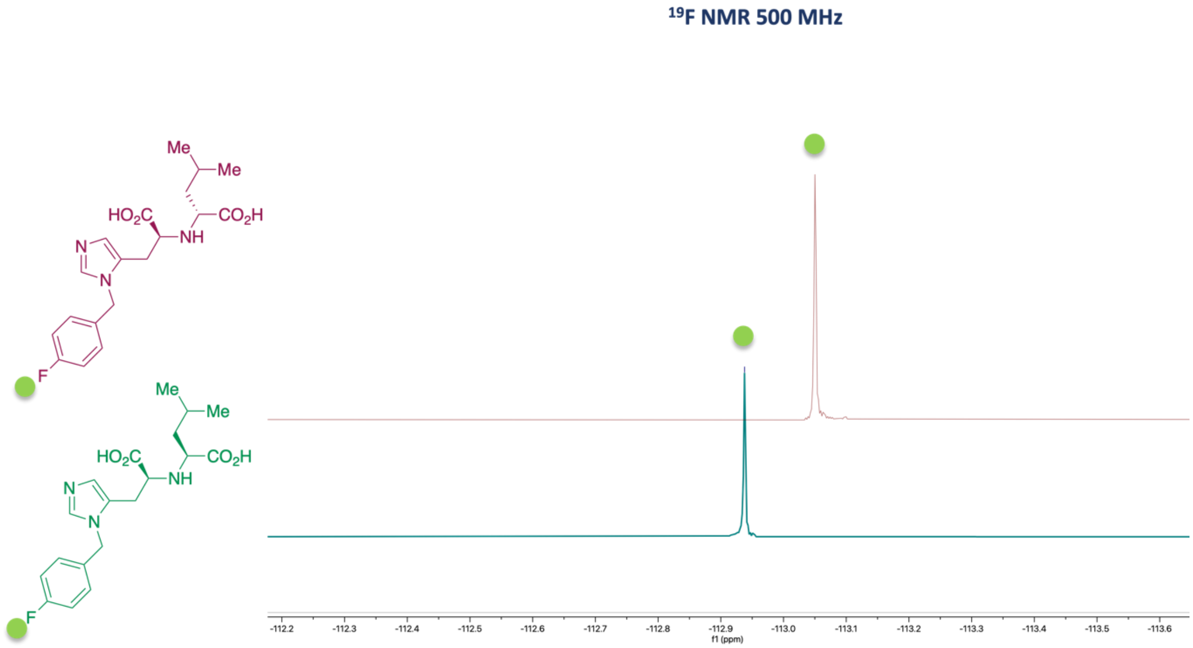

Figure S2.3. Overlapped ^{19}F NMR spectra of (*S*,*R*)-**1** (maroon) and (*S*,*S*)-**1** (green).

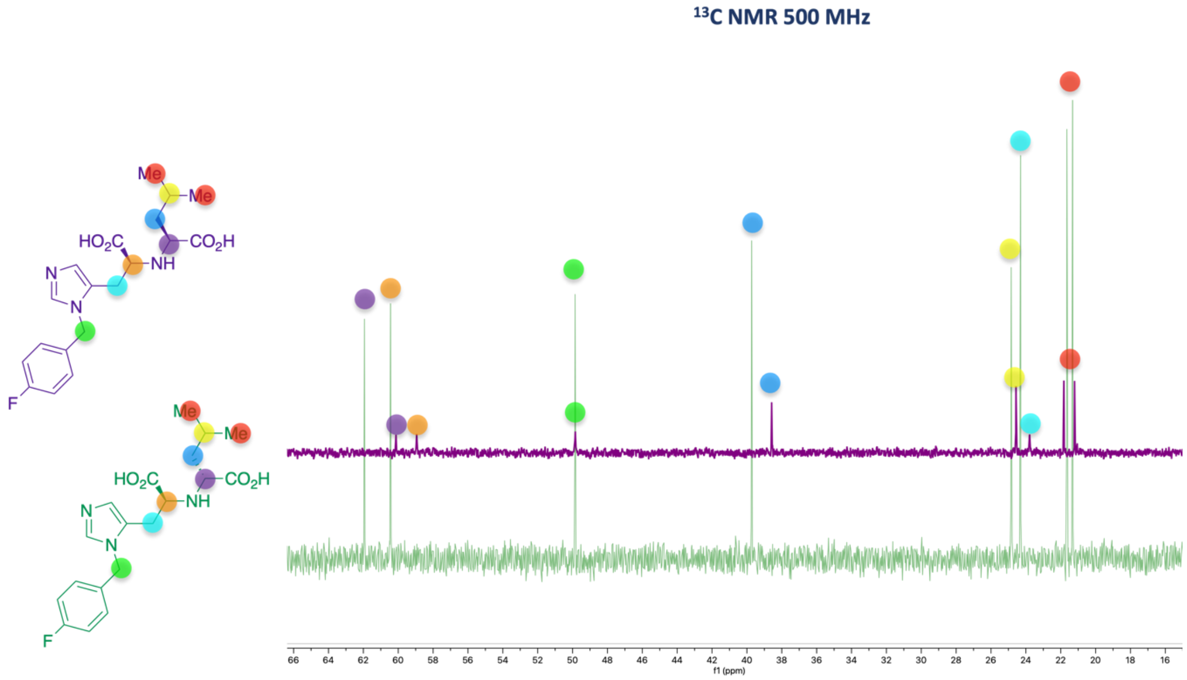

Figure S2.4. Overlapped ^{13}C NMR spectra of (*S*,*R*)-**1** (green) and (*S*,*S*)-**1** (purple).

2.3.2 HPLC analysis

Under identical conditions, different HPLC retention times were observed for the two diastereomers (t_R[(*S*,*S*)-**1**]=11.6 min, t_R[(*S*,*R*)-**1**]=13.9 min) (Figure S2.5). Reverse phase HPLC details: Phenomenex Kinetex® 5 µm C18 100 Å, 250 x 4.6 mm, flow rate = 7 mL/min, column temperature = 25 °C, eluent = 10% MeCN (0.1% TFA) and 90% H_2O (0.1% TFA) for 25 min.

a
b
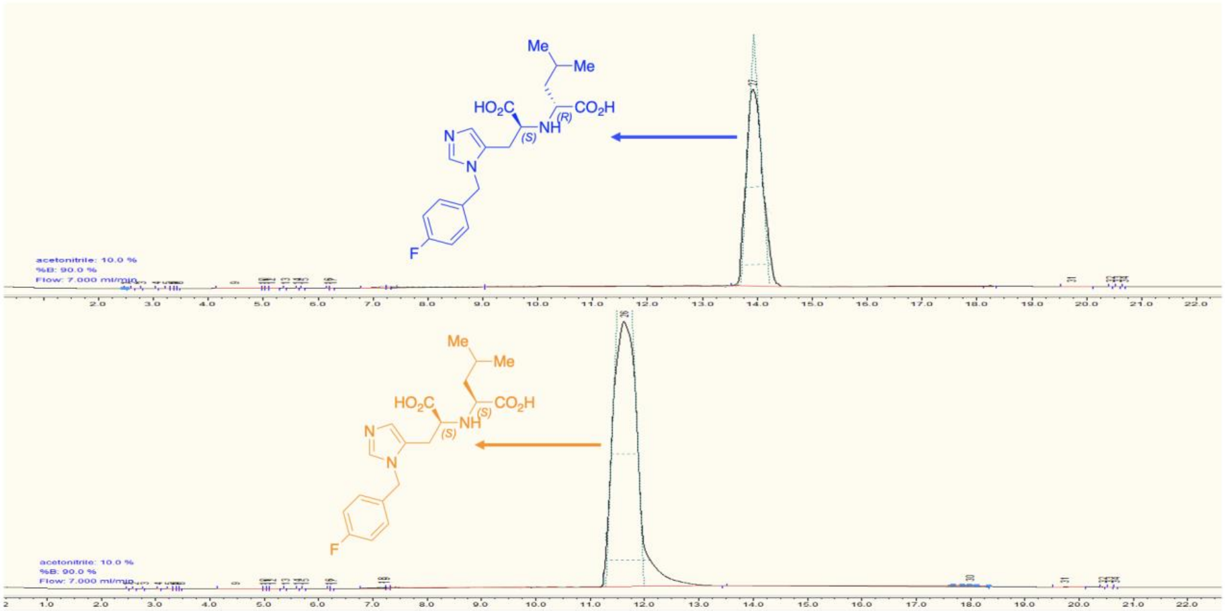

Figure S2.5. HPLC traces for a) (*S*,*R*)-**1**; b) (*S*,*S*)-**1**.

2.4 Synthetic protocols and characterization

***Tert*-butyl (*S*)-4-(2-((*tert*-butoxycarbonyl)amino)-3-methoxy-3-oxopropyl)-1*H*-imidazole-1-carboxylate ((*S*)-S1)**

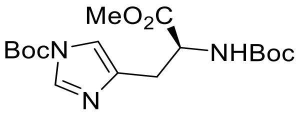

(*S*)-**S1**

Di-*tert*-butyl dicarbonate (Boc_2O, 18.0 g, 82.6 mmol, 2.0 equiv.) in MeOH (20.0 mL) was added slowly via an addition funnel to a solution of *(S)*-histidine methyl ester dihydrochloride ((*S*)-**5**) (10.0 g, 41.3 mmol, 1.0 equiv.) and triethylamine (14.4 mL, 103 mmol, 2.5 equiv.) in MeOH (82.6 mL, 0.5 M). After 16 hours, the reaction mixture was concentrated *in vacuo*, and the residue was redissolved in dichloromethane (CH_2Cl_2) and H_2O (1:1, 40 mL). After separation, the organic phase was washed with brine (25 mL), dried over Na_2SO_4, filtered and concentrated to yield a colorless oil, which was then triturated with hexane, and further purified using flash column chromatography (35% EtOAc in pentane) to obtain the di-Boc protected imidazole (*S*)-**S1** as white powder (13.3 g, 36.0 mmol, 87%).

R_f 0.33 (35% EtOAc/pentane).

^{1}H NMR (500 MHz, $CDCl_3$) δ 7.96 (d, *J* = 1.3 Hz, 1H), 7.12 (d, *J* = 1.3 Hz, 1H), 5.70 (d, *J* = 8.4 Hz, 1H), 4.59 – 4.52 (m, 1H), 3.71 (s, 3H), 3.08 – 2.97 (m, 2H), 1.58 (s, 9H), 1.42 (s, 9H).

^{13}C{^{1}H} NMR (126 MHz, $CDCl_3$) δ 172.4, 155.6, 147.0, 138.8, 137.0, 114.7, 85.7, 79.8, 53.3, 52.4, 30.4, 28.4, 28.0.

Characterization data were in accordance with those found in the literature.[15]

Methyl N^{α}-(*tert*-butoxycarbonyl)-N^{π}-(4-fluorobenzyl)-*L*-histidinate ((*S*)-S2)

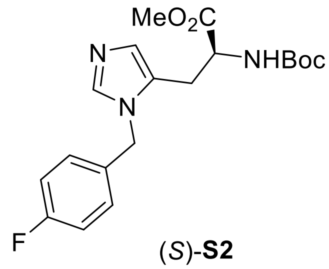

(*S*)-**S2**

The synthesis of (*S*)-**S2** was performed using a modified literature procedure.[16] Under anhydrous conditions, a solution of (4-fluorophenyl)methanol (3.11 g, 24.7 mmol, 1.0 equiv.) and diisopropylethylamine (DIPEA, 4.59 mL, 29.6 mmol, 1.2 equiv.) in CH_2Cl_2 (10.6 mL) was added to a cooled (-78 °C) solution of trifluoromethanesulfonic anhydride (Tf_2O, 4.15 mL, 24.7 mmol, 1.0 equiv.) in CH_2Cl_2 (80.0 mL, 0.25 M). After stirring for 20 minutes at -78 °C, a solution of (*S*)-**S1** (10.0 g, 27.1 mmol, 1.1 equiv.) in CH_2Cl_2 (8.0 mL) was then added to the reaction mixture and was left to stir at room temperature for 24 h. The reaction mixture was then concentrated under reduced pressure and was transferred to a separatory funnel containing EtOAc (25 mL) and H_2O (25 mL). This was extracted with EtOAc (2 × 25 mL) and the combined organics were washed with brine (25 mL), dried over Na_2SO_4, and concentrated under reduced pressure to yield the crude product. The crude product was further purified using flash column chromatography (gradient 0–5% MeOH in CH_2Cl_2) to obtain (*S*)-**S2** as a viscous yellow oil (7.28 g, 19.3 mmol, 78%).

R_f 0.37 (5% MeOH/CH_2Cl_2).

^{1}H NMR (500 MHz, MeOD) δ 7.70 – 7.66 (m, 1H), 7.22 – 7.15 (m, 2H), 7.12 – 7.06 (m, 2H), 6.85 – 6.82 (m, 1H), 5.28 – 5.17 (m, 2H), 4.32 – 4.26 (m, 1H), 3.68 (s, 3H), 2.99 (dd, *J* = 15.5, 5.3 Hz, 1H), 2.85 (dd, *J* = 15.5, 9.1 Hz, 1H), 1.40 (s, 9H).

^{13}C{^{1}H} NMR (126 MHz, MeOD) δ 173.3, 163.8 (d, *J* = 245.3 Hz), 157.6, 139.3, 133.9 (d, *J* = 3.2 Hz), 130.1 (d, *J* = 8.2 Hz), 129.1, 128.5, 116.8 (d, *J* = 21.9 Hz), 80.7, 54.2, 52.8, 30.7, 28.7, 27.2.

^{19}F NMR (471 MHz, MeOD) δ -117.68 – -118.00 (m).

HRMS (ESI$^+$) m/z: $[M+H]^+$ Calcd for $C_{19}H_{25}FN_3O_4$ 378.1824; Found 378.1821.

IR (thin layer film) ν (cm^{-1}) 3119, 2981, 2955, 1745, 1709, 1607, 1513, 1438, 1366, 1276, 1255, 1225, 1161, 1101, 1056, 1031, 831, 771, 639.

$[\alpha]_D^{25\,°C}$ +13.1 (c 0.150, $CHCl_3$).

Methyl N^{π}-(4-fluorobenzyl)-N^{α}-((2-nitrophenyl)sulfonyl)-*L*-histidinate ((*S*)-S6)

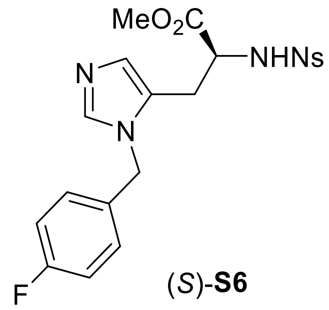

(*S*)-**S6**

Excess HCl (3.98 mL, 4.0 M in dioxane, 5.0 equiv.) was added to (*S*)-**S2** (1.20 g, 3.18 mmol, 1.0 equiv.) and the resulting mixture was stirred for 2 hours. The reaction mixture was then concentrated under reduced pressure and the obtained di-HCl salt was triturated with EtOAc (10 mL) to yield a white solid. This solid was suspended in CH_2Cl_2 (15.9 mL, 0.2 M) and Et_3N (1.33 mL, 9.54 mmol, 3.0 equiv.) was added and stirred for 1 hour. Subsequently, 2-nitrobenzenesulfonyl chloride (1.06 g, 4.77 mmol, 1.5 equiv.) was added to the reaction mixture and stirred overnight at room temperature for 18 hours. The reaction was then quenched with NH_4Cl, and extracted with EtOAc (3 × 20 mL), washed with brine (25 mL), dried over Na_2SO_4, and concentrated under reduced pressure. The crude product was purified via flash column chromatography (2% MeOH in EtOAc) to obtain the product as a viscous yellow oil (1.10 g, 2.38 mmol, 75%).

R_f 0.25 (2% MeOH/EtOAc).

^{1}H NMR (500 MHz, $CDCl_3$) δ 8.01 – 7.97 (m, 1H), 7.91 – 7.87 (m, 1H), 7.76 – 7.68 (m, 2H), 7.48 – 7.44 (m, 1H), 7.10 – 7.03 (m, 4H), 6.81 – 6.78 (m, 1H), 5.21 (d, *J* = 16.0 Hz, 1H), 5.14 (d, *J* = 16.0 Hz, 1H), 4.31 – 4.25 (m, 1H), 3.56 (s, 3H), 3.07 – 3.04 (m, 2H).

^{13}C{^{1}H} NMR (126 MHz, $CDCl_3$) δ 170.2, 162.6 (d, *J* = 247.4 Hz), 147.8, 138.7, 134.0, 133.7, 133.0, 131.8 (d, *J* = 3.2 Hz), 130.6, 129.3, 128.7 (d, *J* = 8.3 Hz), 125.8, 125.7, 116.2 (d, *J* = 21.6 Hz), 56.6, 53.0, 48.3, 28.2.

^{19}F NMR (471 MHz, $CDCl_3$) δ -113.61 – -113.72 (m).

HRMS (ESI$^+$) m/z: $[M+H]^+$ Calcd for $C_{20}H_{20}FN_4O_6S$ 463.1082; Found 463.1084.

IR (thin layer film) ν (cm^{-1}) 3327, 2981, 2950, 2833, 1451, 1393, 1252, 1150, 1119, 1026, 956, 689.

$[\alpha]_D^{25\ °C}$ -31.4 (c 0.015, $CHCl_3$).

Benzyl (*R*)-2-hydroxy-4-methylpentanoate ((*R*)-7)

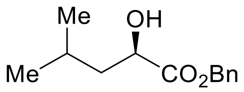

(*R*)-**7**

To a solution of (*R*)-2-hydroxy-4-methylpentanoic acid (4.00 g, 30.3 mmol, 1.0 equiv.) in H_2O (10.0 mL) and MeOH (110 mL), Cs_2CO_3 (9.86 g, 30.3 mmol, 1.0 equiv.) was added and stirred for 30 minutes. The reaction mixture was then concentrated *in vacuo* and redissolved in *N*,*N*-dimethylformamide (DMF) (120 mL, 0.25 M) and cooled to 0 °C. Benzyl bromide (5.18 g, 30.3 mmol, 1.0 equiv.) was added to the reaction mixture and stirred at room temperature for 12 hours. The reaction was quenched with NH_4Cl, extracted with EtOAc (3 × 20 mL), washed with brine (25 mL), dried over Na_2SO_4, and concentrated under reduced pressure. The crude product was purified by flash column chromatography (5% EtOAc in pentane) to yield the product as a colourless liquid (5.50 g, 24.7 mmol, 82%).

R_f 0.45 (5% EtOAc/pentane).

^{1}H NMR (400 MHz, $CDCl_3$) δ 7.41 – 7.32 (m, 5H), 5.23 – 5.19 (m, 2H), 4.25 (ddd, *J* = 7.9, 6.7, 5.4 Hz, 1H), 2.85 – 2.78 (m, 1H), 1.95 – 1.84 (m, 1H), 1.61 – 1.56 (m, 2H), 1.00 – 0.89 (m, 6H).

^{13}C{^{1}H} NMR (101 MHz, $CDCl_3$) δ 175.8, 135.4, 128.7, 128.6, 128.4, 69.3, 67.3, 43.5, 24.5, 23.3, 21.7.

HRMS (ESI^+) m/z: $[M+Na]^+$ Calcd for $C_{13}H_{18}O_3Na$ 245.1148; Found 245.1154.

IR (thin layer film) ν (cm^{-1}) 3492, 3035, 2958, 2871, 1737, 1499, 1469, 1457, 1386, 1369, 1269, 1214, 1141, 1088, 1005, 962, 850, 804, 749, 698.

$[\alpha]_D^{25\ °C}$ +11.4 (c 0.010, $CHCl_3$).

Benzyl ((*S*)-3-(1-(4-fluorobenzyl)-1*H*-imidazol-5-yl)-1-methoxy-1-oxopropan-2-yl)-*L*-leucinate ((*S,S*)-11)

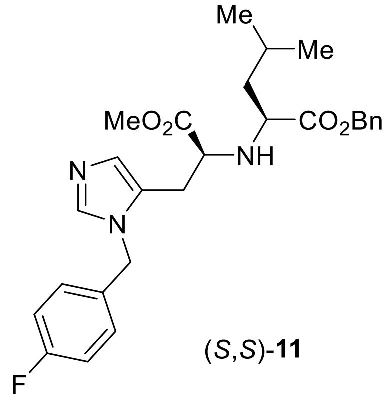

(*S*,*S*)-**11**

Triphenylphosphine (PPh_3, 189 mg, 0.720 mmol, 1.0 equiv.) and diisopropyl azodicarboxylate (DIAD, 141 μL, 0.720 mmol, 1.0 equiv.) was mixed in THF (3.60 mL, 0.2 M) at -10 °C under inert atmosphere and the mixture was left to stir for 60 minutes. Subsequently, (*R*)-**7** (160 mg, 0.720 mmol, 1.0 equiv.) was added and stirred for 2 hours. Then *(S)*-**S6** (500 mg, 1.08 mmol 1.5 equiv.) was added and the reaction mixture was warmed to room temperature and stirred overnight. After 16 hours, the mixture was concentrated *in vacuo*, and the resulting residue was redissolved in EtOAc and H_2O (1:1, 20 mL). The aqueous layer was extracted with EtOAc (3 × 10 mL), and the combined organic layer was washed with brine (25 mL), dried over Na_2SO_4 and concentrated under reduced pressure. The resulting crude product was used directly in the next step. Then potassium carbonate (K_2CO_3, 149 mg, 1.08 mmol, 1.5 equiv.) was added to this crude intermediate in *N*,*N*-dimethylformamide (3.60 mL, 0.2 M) at room temperature, thiophenol (PhSH, 111 μL, 1.08 mmol, 1.5 equiv.) was added and the resulting mixture was stirred for 1 hour at room temperature. The solution was then partitioned between equal volumes of EtOAc and H_2O, and the aqueous layer was further extracted with EtOAc (3 × 20 mL). The combined organic layer was washed with brine (25 mL), dried over Na_2SO_4 and concentrated under reduced pressure. The resulting residue was purified by flash column chromatography (2% MeOH in EtOAc) to afford the product as a viscous cream oil (118 mg, 0.245 mmol, 34%, > 20:1 *d.r.*).

Alternative resequenced procedure: Di-*tert*-butyl dicarbonate (Boc_2O, 350 mg, 1.60 mmol, 2.2 equiv.) in CH_2Cl_2 (0.65 mL) was slowly added to a solution of (*S*,*S*)-**9** (300 mg, 0.802 mmol, 1.1

equiv.) and DIPEA (254 μL, 1.46 mmol, 2.0 equiv.) in CH_2Cl_2 (3.00 mL, 0.2 M) at room temperature. After stirring for 24 hours, the reaction mixture was concentrated under reduced pressure, and the residue was redissolved in dichloromethane (CH_2Cl_2) and H_2O (1:1, 10 mL). After separation, and the organic phase was washed with brine, dried over Na_2SO_4, filtered, and concentrated to obtain a crude intermediate. Separately, a solution of trifluoromethanesulfonic anhydride (Tf_2O, 122 μL, 0.729 mmol, 1.0 equiv.) in CH_2Cl_2 (3.00 mL, 0.2 M) was cooled to -78 °C. To this solution, (4-fluorophenyl)methanol **10** (92.0 mg, 0.729 mmol, 1.0 equiv.) and diisopropylethylamine (DIPEA, 191 μL, 1.09 mmol, 1.5 equiv.) in CH_2Cl_2 (0.35 mL) were added. After stirring for 20 minutes, to this solution was added a solution of the crude intermediate in CH_2Cl_2 (0.30 mL) was added, and the reaction mixture was slowly brought to room temperature and stirred overnight. Thereafter, HCl (0.201 mL, 4.0 M in dioxane, 1.1 equiv.) was added to the reaction mixture and stirred for 1 hour. The reaction mixture was then concentrated and the organic phase was extracted with EtOAc (3 × 25 mL), washed with brine (25 mL), dried over Na_2SO_4, and concentrated to yield the crude product. The crude product was further purified using flash column chromatography (gradient 0 - 10% MeOH in EtOAc) to obtain the product as a viscous cream oil (150 mg, 0.311 mmol, 43%, > 20:1 *d.r.*).

R_f 0.32 (2% MeOH/EtOAc).
^{1}H NMR (500 MHz, $CDCl_3$) δ 8.60 – 8.56 (m, 1H), 7.41 – 7.30 (m, 6H), 7.23 – 7.16 (m, 2H), 7.14 – 7.07 (m, 2H), 5.38 – 5.28 (m, 2H), 5.14 – 5.11 (m, 2H), 3.74 (s, 3H), 3.56 – 3.50 (m, 1H), 3.47 (dd, *J* = 8.2, 5.5 Hz, 1H), 3.01 (dd, *J* = 15.5, 5.6 Hz, 1H), 2.88 (dd, *J* = 15.6, 6.8 Hz, 1H), 1.72 – 1.61 (m, 1H), 1.57 (ddd, *J* = 13.7, 8.2, 5.6 Hz, 1H), 1.46 (ddd, *J* = 13.9, 8.2, 6.1 Hz, 1H), 0.91 (d, *J* = 6.6 Hz, 3H), 0.89 (d, *J* = 6.5 Hz, 3H).
^{13}C{^{1}H} NMR (126 MHz, $CDCl_3$) δ 174.1, 172.6, 163.4 (d, *J* = 250.0 Hz), 135.5, 130.6, 130.0 (d, *J* = 8.5 Hz), 128.9, 128.8, 128.5, 128.2 (d, *J* = 3.3 Hz), 119.7, 117.0 (d, *J* = 21.9 Hz), 67.2, 59.0, 58.9, 53.0, 50.5, 42.2, 27.6, 24.9, 22.9, 21.9.
^{19}F NMR (471 MHz, $CDCl_3$) δ -110.86 – -110.95 (m).
HRMS (ESI$^+$) m/z: $[M+H]^+$ Calcd for $C_{27}H_{33}FN_3O_4$ 482.2450; Found 482.2451.
IR (thin layer film) ν (cm^{-1}) 3335, 2968, 2358, 1619, 1598, 1505, 1426, 1379, 1277, 1176, 1135, 888, 813, 750, 700, 682.
$[\alpha]_D^{25\ °C}$ -16.3 (c 0.020, $CHCl_3$).

((*S*)-1-Carboxy-2-(1-(4-fluorobenzyl)-1*H*-imidazol-5-yl)ethyl)-*L*-leucine ((*S*,*S*)-1)

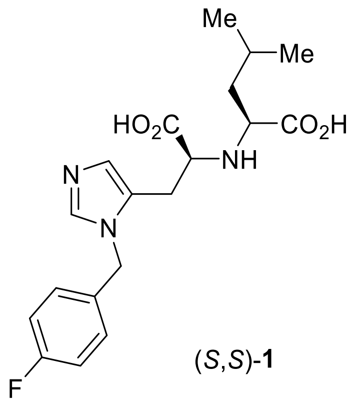

(*S*,*S*)-**1**

(*S,S*)-**11** (100 mg, 0.208 mmol, 1.0 equiv.) was subjected to hydrolysis using NaOH (24.9 mg, 0.623 mmol, 3.0 equiv.) in H_2O and MeOH (1:1, 0.415 mL, 0.5 M). The resulting mixture was stirred for 1 hour at room temperature, followed by the addition of aqueous HCl (1.0 M) to adjust the pH to 7.0. The mixture was stirred for an additional 30 minutes and then concentrated under reduced pressure and obtained the crude product (74.0 mg, 0.196 mmol, 94%, > 20:1 d.r.). The crude product was further purified using semi-preparative HPLC to obtain (*S,S*)-**1** as a white solid (38.0 mg, 101 μmol, 48%).

Reverse phase HPLC details: Phenomenex Kinetex® 5 μm C18 100 Å, 250 x 4.6 mm, flow rate = 7 mL/min, column temperature = 25 °C, eluent = 10% MeCN (0.1% TFA) and 90% H_2O (0.1% TFA) for 25 min, retention time = 11.6 min;

^{1}H NMR (500 MHz, D_2O) δ 8.67 (d, *J* = 1.7 Hz, 1H), 7.53 (d, *J* = 1.5 Hz, 1H), 7.41 – 7.33 (m, 2H), 7.24 – 7.18 (m, 2H), 5.43 – 5.39 (m, 2H), 3.69 – 3.63 (m, 1H), 3.58 – 3.55 (m, 1H), 3.31 – 3.29 (m, 1H), 3.29 – 3.27 (m, 1H), 1.83 – 1.75 (m, 1H), 1.73 – 1.68 (m, 2H), 0.94 (d, *J* = 4.4 Hz, 3H), 0.93 (d, *J* = 4.6 Hz, 3H).

^{13}C{^{1}H} NMR (126 MHz, D_2O) δ 173.8, 171.0, 162.8 (d, *J* = 246.1 Hz), 135.6, 130.3 (d, *J* = 8.6 Hz), 129.0, 128.6 (d, *J* = 3.2 Hz), 119.2, 116.2 (d, *J* = 21.8 Hz), 61.9, 60.4, 49.9, 39.7, 24.8, 24.3, 21.7, 21.3.

^{19}F NMR (471 MHz, D_2O) δ -115.60 (tt, *J* = 10.4, 5.2 Hz).

HRMS (ESI^+) m/z: $[M+H]^+$ Calcd for $C_{19}H_{25}FN_3O_4$ 378.1824; Found 378.1821.

IR (thin layer film) ν (cm^{-1}) 3449, 3125, 3051, 2971, 2943, 2879, 1744, 1682, 1620, 1514, 1465, 1436, 1388, 1304, 1187, 1041, 939, 841, 825, 798, 724, 687, 657.

MP 198–200 °C.

[α]$_D$$^{25\ °C}$ +32.2 (c 0.015, H_2O).

Benzyl (*S*)-2-hydroxy-4-methylpentanoate ((*S*)-7)

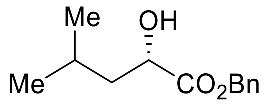

(*S*)-**7**

To a solution of *(S)*-2-hydroxy-4-methylpentanoic acid (2.00 g, 15.1 mmol, 1.0 equiv.) in H_2O (5.50 mL) and MeOH (55.0 mL, 0.25 M), Cs_2CO_3 (4.93 g, 15.1 mmol, 1.0 equiv.) was added and stirred for 30 minutes. The reaction mixture was then concentrated *in vacuo* and redissolved in *N*,*N*-dimethylformamide (DMF, 60.5 mL, 0.25 M) and cooled to 0 °C. Benzyl bromide (BnBr, 2.59 g, 15.1 mmol, 1.0 equiv.) was added to the solution and stirred at room temperature for 12 hours. The reaction was quenched with NH_4Cl, extracted with EtOAc (3 × 10 mL), washed with brine (25 mL), dried over Na_2SO_4, and concentrated under reduced pressure. The crude product was purified by flash column chromatography (5% EtOAc in pentane) to yield the product as a colourless liquid (3.00 g, 13.5 mmol, 89%).

R_f 0.42 (5% EtOAc/pentane).

^{1}H NMR (500 MHz, $CDCl_3$) δ 7.42 – 7.31 (m, 5H), 5.22 (d, *J* = 12.3 Hz, 1H), 5.19 (d, *J* = 12.4 Hz, 1H), 4.24 (ddd, *J* = 8.5, 6.0, 4.7 Hz, 1H), 2.67 – 2.61 (m, 1H), 1.95 – 1.83 (m, 1H), 1.64 – 1.51 (m, 2H), 0.94 (d, *J* = 5.5 Hz, 3H), 0.93 (d, *J* = 5.8 Hz, 3H).

^{13}C{^{1}H} NMR (126 MHz, $CDCl_3$) δ 175.9, 135.4, 128.8, 128.7, 128.5, 69.3, 67.4, 43.6, 24.6, 23.4, 21.7.

HRMS (ESI$^+$) m/z: [M+Na]$^+$ Calcd for $C_{13}H_{18}O_3Na$ 245.1148; Found 245.1159.

IR (thin layer film) ν (cm^{-1}) 3469, 3035, 2958, 2871, 1737, 1499, 1469, 1456, 1386, 1369, 1270, 1214, 1141, 1088, 1004, 962, 850, 749, 698.

[α]$_D$$^{25\ °C}$ -11.2 (c 0.025, $CHCl_3$).

Benzyl ((*S*)-3-(1-(4-fluorobenzyl)-1*H*-imidazol-5-yl)-1-methoxy-1-oxopropan-2-yl)-*D*-leucinate ((*S*,*R*)-11)

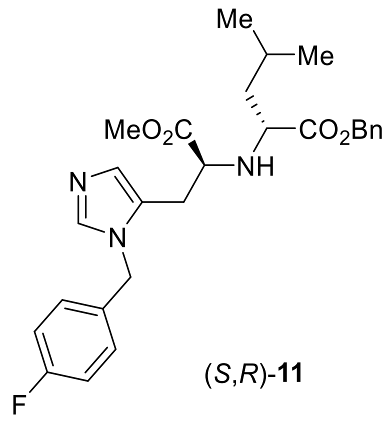

(*S*,*R*)-**11**

Triphenylphosphine (PPh_3, 113 mg, 0.432 mmol, 1.0 equiv.) and diisopropyl azodicarboxylate (DIAD, 84.8 μL, 0.432 mmol, 1.0 equiv.) was mixed in THF (2.16 mL, 0.2 M) at -10 °C under inert atmosphere and the mixture was left to stir for 60 minutes. Subsequently, *(S)*-**7** (96.0 mg, 0.432 mmol, 1.0 equiv.) was added and stirred for 2 hours. Then *(S)*-**S6** (300 mg, 0.648 mmol 1.5 equiv.) were added and the reaction mixture was warmed to room temperature and stirred overnight. After 16 hours, the mixture was concentrated *in vacuo*, and the resulting residue was redissolved in EtOAc and H_2O (1:1, 20 mL). The aqueous layer was extracted with EtOAc (3 × 10 mL), and the combined organic layer was washed with brine (25 mL), dried over Na_2SO_4 and concentrated under reduced pressure. The resulting crude product was used directly in the next step. Then potassium carbonate (K_2CO_3, 89.5 mg, 0.648 mmol, 1.5 equiv.) was added to this crude intermediate in *N*,*N*-dimethylformamide (2.16 mL, 0.2 M) at room temperature, thiophenol (PhSH, 66.3 μL, 0.648 mmol, 1.5 equiv.) was added and the resulting mixture was stirred for 1 hour at room temperature. The solution was then partitioned between equal volumes of EtOAc and H_2O, and the aqueous layer was further extracted with EtOAc (3 × 10 mL). The combined organic layer was washed with brine (25 mL), dried over Na_2SO_4 and concentrated under reduced pressure. The resulting residue was purified by flash column chromatography (2% MeOH in EtOAc) to afford the product as a viscous cream oil (68.0 mg, 0.141 mmol, 33%, > 20:1 d.r.).

R_f 0.32 (2% MeOH/EtOAc).

^{1}H NMR (500 MHz, $CDCl_3$) δ 8.62 – 8.58 (m, 1H), 7.40 – 7.31 (m, 6H), 7.18 – 7.14 (m, 2H), 7.14 – 7.08 (m, 2H), 5.31 – 5.27 (m, 2H), 5.13 – 5.10 (m, 2H), 3.63 (s, 3H), 3.42 – 3.36 (m, 1H), 3.24 (dd, *J* = 8.1, 6.3 Hz, 1H), 2.93 – 2.81 (m, 2H), 1.67 – 1.54 (m, 1H), 1.53 – 1.46 (m, 1H), 1.46 – 1.37 (m, 1H), 0.87 (d, *J* = 6.6 Hz, 3H), 0.80 (d, *J* = 6.6 Hz, 3H).

^{13}C{^{1}H} NMR (126 MHz, $CDCl_3$) δ 174.1, 172.7, 163.4 (d, *J* = 249.8 Hz), 135.5, 130.6, 130.0 (d, *J* = 8.6 Hz), 128.9, 128.8, 128.5, 128.3 (d, *J* = 3.2 Hz), 119.7, 117.0 (d, *J* = 21.8 Hz), 67.2, 59.0, 59.0, 53.0, 50.5, 42.2, 27.7, 25.0, 22.9, 21.9.

^{19}F NMR (471 MHz, $CDCl_3$) δ -110.67 – -110.76 (m).

HRMS (ESI^+) m/z: $[M+H]^+$ Calcd for $C_{27}H_{33}FN_3O_4$ 482.2450; Found 482.2452.

IR (thin layer film) ν (cm^{-1}) 3332, 2971, 2659, 1768, 1467, 1411, 1379, 1309, 1161, 1129, 952, 817, 701, 675.

$[\alpha]_D^{25\,°C}$ +6.3 (c 0.010, $CHCl_3$).

((*S*)-1-Carboxy-2-(1-(4-fluorobenzyl)-1*H*-imidazol-5-yl)ethyl)-*D*-leucine ((*S*,*R*)-1)

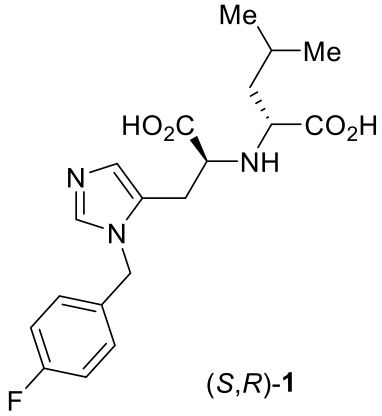

(*S*,*R*)-**1**

(*S*,*R*)-**11** (100 mg, 0.208 mmol, 1.0 equiv.) was subjected to hydrolysis using NaOH (24.9 mg, 0.623 mmol, 3.0 equiv.) in H_2O and MeOH (1:1, 0.415 mL, 0.5 M). The resulting mixture was stirred for 1 hour at room temperature, followed by the addition of aqueous HCl (1.0 M) to adjust the pH to 7.0. The mixture was stirred for an additional 30 minutes and then concentrated under reduced pressure and obtained the crude product (62.0 mg, 0.164 mmol, 79%, > 20:1 d.r.). The crude product was further purified using semi-preparative HPLC to obtain the desired product as a white solid (35.0 mg, 92.7 μmol, 45%).

Reverse phase HPLC details: Phenomenex Kinetex® 5 μm C18 100 Å, 250 x 4.6 mm, flow rate = 7 mL/min, column temperature = 25 °C, eluent = 10% MeCN (0.1% TFA) and 90% H_2O (0.1% TFA) for 25 min, retention time = 13.9 min;

^{1}H NMR (500 MHz, D_2O) δ 8.73 – 8.70 (m, 1H), 7.49 – 7.45 (m, 1H), 7.40 – 7.32 (m, 2H), 7.26 – 7.18 (m, 2H), 5.48 – 5.38 (m, 2H), 3.71 – 3.64 (m, 1H), 3.56 – 3.50 (m, 1H), 3.33 – 3.21 (m, 2H), 1.75 – 1.60 (m, 2H), 1.59 – 1.50 (m, 1H), 0.93 – 0.87 (m, 6H).

^{13}C{^{1}H} NMR (126 MHz, D_2O) δ 173.7, 171.1, 162.8 (d, *J* = 246.1 Hz), 135.6, 130.0 (d, *J* = 9.1 Hz), 129.2 (d, *J* = 2.8 Hz), 128.7 (d, *J* = 3.2 Hz), 119.1, 116.3 (d, *J* = 22.0 Hz), 60.1, 58.9, 49.9, 38.6, 24.6, 23.8, 21.8, 21.2.

^{19}F NMR (471 MHz, D_2O) δ -112.93 (tt, *J* = 10.4, 5.2 Hz, 1F).

HRMS (ESI^+) m/z: $[M+H]^+$ Calcd for $C_{19}H_{25}FN_3O_4$ 378.1824; Found 378.1825.

IR (thin layer film) ν (cm^{-1}) 3449, 3124, 3046, 2971, 2943, 2880, 1744, 1676, 1621, 1515, 1464, 1436, 1388, 1304, 1188, 939, 841, 825, 798, 724, 636.

MP 198 - 200 °C.

$[\alpha]_D^{25\ °C}$ +3.5 (c 0.050, H_2O).

Methyl N^{π}-(4-fluorobenzyl)-N^{α}-((2-nitrophenyl)sulfonyl)-*L*-histidinate ((*S*)-6)

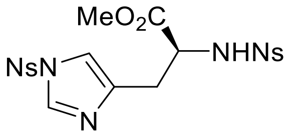

(*S*)-**6**

(S)-**5** (2.00 g, 8.26 mmol, 1.0 equiv.) was stirred with triethylamine (Et_3N, 3.68 mL, 26.4 mmol, 3.0 equiv.) in dichloromethane (CH_2Cl_2, 33.0 mL, 0.25 M) for 60 minutes at room temperature. 2-Nitrobenzenesulfonyl chloride (4.03 g, 18.2 mmol, 2.2 equiv.) was added to the solution, and the reaction mixture was stirred overnight at room temperature for 18 hours. After completion of the reaction, the mixture was concentrated and extracted with EtOAc (3 × 20 mL), and the combined organic layer was washed with brine (25 mL), dried over Na_2SO_4, and concentrated under reduced pressure. The resulting crude product was purified using flash column chromatography (20% pentane in EtOAc) to afford the product as a yellow solid (3.40 g, 6.30 mmol, 76%).

R_f = 0.33 (20% pentane/EtOAc).

^{1}H NMR (400 MHz, $CDCl_3$) δ 8.07 – 8.02 (m, 1H), 8.02 – 7.98 (m, 1H), 7.96 (d, *J* = 1.3 Hz, 1H), 7.91 – 7.80 (m, 4H), 7.75 – 7.69 (m, 2H), 7.22 – 7.20 (m, 1H), 6.52 (d, *J* = 8.4 Hz, 1H), 4.53 (ddd, *J* = 8.4, 6.3, 5.0 Hz, 1H), 3.54 (s, 3H), 3.14 – 3.08 (m, 2H).

^{13}C{^{1}H} NMR (101 MHz, $CDCl_3$) [overlapping signals] δ 170.8, 139.1, 137.7, 136.2, 134.5, 133.7, 133.5, 133.0, 131.2, 130.9, 130.5, 125.7, 125.6, 116.1, 55.9, 52.8, 31.4.

HRMS (ESI$^+$) m/z: $[M+H]^+$ Calcd for $C_{19}H_{18}N_5O_{10}S_2$ 540.0490; Found 540.0480.

IR (thin layer film) ν (cm^{-1}) 3657, 2981, 2889, 1746, 1543, 1473, 1462, 1442, 1390, 1252, 1165, 1081, 955, 854, 762, 743, 655, 613.

MP 72–74 °C.

$[\alpha]_D^{25\,°C}$ -42.2 (c 0.015, $CHCl_3$).

Benzyl *N*-((*S*)-1-methoxy-3-(1-((4-nitrophenyl)sulfonyl)-1*H*-imidazol-4-yl)-1-oxopropan-2-yl)-*N*-((4-nitrophenyl)sulfonyl)-*L*-leucinate ((*S*,*S*)-8)

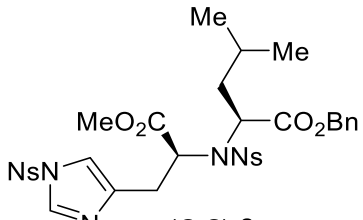

(*S*,*S*)-**8**

A mixture of diisopropyl azodicarboxylate (DIAD, 121 μL, 0.618 mmol, 1.0 equiv.) and triphenylphosphine (PPh_3, 162 mg, 0.618 mmol, 1.0 equiv.) was mixed in tetrahydrofuran (THF, 3.09 mL, 0.2 M) under an inert atmosphere at -10 °C. The mixture was left to stir for 60 minutes. Subsequently, *(S)*-**6** (500 mg, 0.927 mmol, 1.5 equiv.) were added to the reaction mixture and stirred for another 2 hours at room temperature. Then (*R*)-**7** (137 mg, 0.618 mmol, 1.0 equiv.) was added and the reaction mixture was stirred overnight for 16 hours. After that, the mixture was concentrated *in vacuo*, and the resulting residue was redissolved in EtOAc and H_2O (1:1, 20 mL). The aqueous layer was extracted with EtOAc (3 × 10 mL), and the combined organic layer was washed with brine (25 mL), dried over Na_2SO_4 and concentrated under reduced pressure. The residue was purified using flash column chromatography (2% MeOH in EtOAc) to yield the product as a viscous yellow oil (252 mg, 0.339 mmol, 55%, > 20:1 *d.r.*).

R_f 0.27 (2% MeOH/EtOAc).

^{1}H NMR (500 MHz, $CDCl_3$) δ 8.19 (dd, *J* = 7.9, 1.3 Hz, 1H), 8.11 (dd, *J* = 7.7, 1.7 Hz, 1H), 8.06 (d, *J* = 1.4 Hz, 1H), 7.90 – 7.82 (m, 2H), 7.79 (dd, *J* = 7.5, 1.7 Hz, 1H), 7.77 – 7.68 (m, 1H), 7.49 (ddd, *J* = 8.3, 7.3, 1.3 Hz, 1H), 7.47 – 7.43 (m, 1H), 7.34 – 7.29 (m, 3H), 7.28 (d, *J* = 1.4 Hz, 1H), 7.18 – 7.15 (m, 2H), 5.00 – 4.94 (m, 1H), 4.92 – 4.80 (m, 2H), 4.43 (dd, *J* = 10.1, 5.1 Hz, 1H), 3.59 (s, 3H), 3.50 (dd, *J* = 14.7, 7.0 Hz, 1H), 3.31 (dd, *J* = 14.8, 7.8 Hz, 1H), 1.78 (ddd, *J* = 13.3, 10.1, 5.2 Hz, 1H), 0.98 – 0.83 (m, 2H), 0.77 (d, *J* = 6.6 Hz, 3H), 0.74 (d, *J* = 6.6 Hz, 3H).

^{13}C{^{1}H} NMR (126 MHz, $CDCl_3$) δ 170.6, 169.8, 149.1, 148.1, 140.4, 137.1, 136.2, 135.0, 133.9, 133.4, 133.2, 132.6, 131.7, 131.4, 131.3, 128.6, 128.5, 128.5, 125.2, 123.4, 116.8, 67.2, 59.1, 58.2, 52.7, 39.6, 31.2, 25.0, 23.1, 21.6.

HRMS (ESI^+) m/z: $[M+H]^+$ Calcd for $C_{32}H_{34}N_5O_{12}S_2$ 744.1640; Found 744.1634.

IR (thin layer film) ν (cm^{-1}) 2958, 2927, 2855, 1738, 1713, 1546, 1468, 1287, 1183, 1082, 924, 852, 779, 697, 655.

$[\alpha]_D^{25\,°C}$ -12.8 (c 0.050, $CHCl_3$).

Benzyl ((*S*)-3-(1*H*-imidazol-4-yl)-1-methoxy-1-oxopropan-2-yl)-*L*-leucinate ((*S*,*S*)-9)

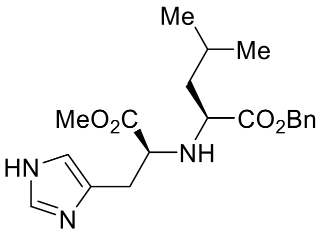

(*S*,*S*)-**9**

Thiophenol (60.4 μL, 0.592 mmol, 2.2 equiv.) was added to a solution of (*S*,*S*)-**8** (200 mg, 0.269 mmol, 1.0 equiv.) and potassium carbonate (K_2CO_3, 111 mg, 0.807 mmol, 3.0 equiv.) in *N*,*N*-dimethylformamide (DMF, 2.69 mL, 0.1 M) at room temperature and stirred for 1 hour. The resulting solution was partitioned between equal volumes of EtOAc and H_2O. The aqueous layer was extracted with EtOAc (3 × 20 mL), and the combined organic layer was washed with brine (25 mL), dried over Na_2SO_4 and concentrated under reduced pressure. The residue was purified by flash column chromatography (2% MeOH in EtOAc) to afford the product as a viscous colourless oil (70.0 mg, 0.187 mmol, 70 %, > 20:1 *d.r.*).

R_f = 0.22 (2% MeOH/CH_2Cl_2).

^{1}H NMR (500 MHz, $CDCl_3$) δ 7.47 – 7.43 (m, 1H), 7.39 – 7.27 (m, 5H), 6.81 – 6.77 (m, 1H), 5.13 – 5.11 (m, 2H), 3.70 (s, 3H), 3.50 (dd, *J* = 8.7, 3.8 Hz, 1H), 3.41 (dd, *J* = 7.9, 6.0 Hz, 1H), 2.99 (dd, *J* = 15.3, 3.8 Hz, 1H), 2.82 (ddd, *J* = 15.3, 8.7, 1.0 Hz, 1H), 1.77 – 1.66 (m, 1H), 1.62 – 1.49 (m, 2H), 0.96 – 0.88 (m, 6H).

^{13}C{^{1}H} NMR (126 MHz, $CDCl_3$) δ 175.5, 174.1, 135.5, 134.9, 128.8, 128.6, 128.4, 67.0, 59.9, 58.8, 52.3, 42.9, 25.0, 23.0, 22.1. *note: broad peaks at 122 ppm and 129 ppm were found by HSQC and HMBC experiments.*

HRMS (ESI^+) m/z: $[M+H]^+$ Calcd for $C_{20}H_{28}N_3O_4$ 374.2074; Found 374.2074.

IR (thin layer film) ν (cm^{-1}) 3502, 3153, 3050, 2880, 1749, 1632, 1561, 1474, 1446, 1282, 1251, 1180, 1032, 963, 944, 765, 644.

$[\alpha]_D^{25\,°C}$ -14.6 (c 0.010, $CHCl_3$).

Benzyl ((*S*)-3-(1-(3-fluoro-5-methylbenzyl)-1*H*-imidazol-5-yl)-1-methoxy-1-oxopropan-2-yl)-*L*-leucinate ((*S*,*S*)-15)

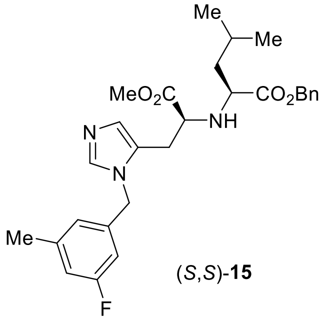

(*S*,*S*)-**15**

Di-*tert*-butyl dicarbonate (Boc_2O, 350 mg, 1.60 mmol, 2.2 equiv.) in CH_2Cl_2 (0.65 mL) was slowly added to a solution of (*S*,*S*)-**9** (300 mg, 0.802 mmol, 1.1 equiv.) and DIPEA (254 µL, 1.46 mmol, 2.0 equiv.) in CH_2Cl_2 (3.00 mL, 0.2 M) at room temperature. After stirring for 24 hours, the reaction mixture was concentrated in *vacuo*, and the residue was redissolved in CH_2Cl_2 and H_2O (1:1, 10 mL). After separation, and the organic phase was washed with brine, dried over Na_2SO_4, filtered, and concentrated to obtain a crude intermediate. Separately, a solution of trifluoromethanesulfonic anhydride (Tf_2O, 122 µL, 0.729 mmol, 1.0 equiv.) in CH_2Cl_2 (3.00 mL, 0.2 M) was cooled to -78 °C. To this solution, (3-fluoro-5-methylphenyl)methanol (**12**, 102 mg, 0.729 mmol, 1.0 equiv.) and diisopropylethylamine (DIPEA, 191 µL, 1.09 mmol, 1.5 equiv.) in CH_2Cl_2 (0.35 mL) were added. After stirring for 20 minutes, the crude intermediate obtained previously was dissolved in CH_2Cl_2 (0.30 mL) was added, and the reaction mixture was slowly brought to room temperature and stirred for 24 hours. Thereafter, HCl (0.201 mL, 4.0 M in dioxane, 1.1 equiv.) was added to the reaction mixture and stirred for 1 hour. The reaction mixture was then concentrated and the organic phase was extracted with EtOAc (3 × 25 mL), washed with brine (25 mL), dried over Na_2SO_4, and concentrated to yield the crude product. The crude product was further purified using flash column chromatography (gradient 0 - 10% MeOH in EtOAc) to obtain product as a viscous cream oil (170 mg, 0.343 mmol, 47%, > 20:1 d.r.).

R_f 0.30 (5% MeOH/EtOAc).

^{1}H NMR (500 MHz, $CDCl_3$) δ 8.63 – 8.59 (m, 1H), 7.41 – 7.30 (m, 6H), 6.97 – 6.83 (m, 1H), 6.76 – 6.73 (m, 1H), 6.72 – 6.67 (m, 1H), 5.33 – 5.29 (m, 2H), 5.14 (d, *J* = 12.4 Hz, 1H), 5.11 (d, *J* = 12.5 Hz, 1H), 3.75 (s, 3H), 3.57 – 3.52 (m, 1H), 3.49 (dd, *J* = 8.3, 5.6 Hz, 1H), 3.02 (dd, *J* = 15.9,

5.7 Hz, 1H), 2.90 (dd, *J* = 15.6, 6.9 Hz, 1H), 2.35 (s, 3H), 1.70 – 1.62 (m, 1H), 1.61 – 1.54 (m, 1H), 1.52 – 1.43 (m, 1H), 0.91 (d, *J* = 6.6 Hz, 3H), 0.89 (d, *J* = 6.6 Hz, 3H).

^{13}C{^{1}H} NMR (126 MHz, $CDCl_3$) δ 174.6, 173.2, 163.1 (d, *J* = 248.9 Hz), 136.0, 135.7, 130.3 (d, *J* = 27.2 Hz), 129.8 (d, *J* = 8.6 Hz), 129.5 (d, *J* = 8.6 Hz), 129.3 (d, *J* = 3.2 Hz), 128.8, 128.8, 128.7, 128.5, 116.7 (d, *J* = 21.8 Hz), 116.6 (d, *J* = 22.3 Hz), 66.9, 58.7 (d, *J* = 16.3 Hz), 52.7, 50.0, 42.4, 27.9, 24.9, 23.0, 22.7, 22.1, 21.9.

^{19}F NMR (471 MHz, $CDCl_3$) δ -112.21 – -112.30 (m).

HRMS (ESI^+) m/z: $[M+H]^+$ Calcd for $C_{28}H_{35}FN_3O_4$ 496.2606; Found 496.2610.

IR (thin layer film) ν (cm^{-1}) 3493, 3468, 3091, 3067, 3035, 2957, 2871, 1734, 1618, 1456, 1369, 1267, 1213, 1140, 1087, 1029, 1004, 850, 805, 748, 697.

$[\alpha]_D^{25\,°C}$ -12.7 (c 0.005, $CHCl_3$).

((*S*)-1-Carboxy-2-(1-(3-fluoro-5-methylbenzyl)-1*H*-imidazol-5-yl)ethyl)-*L*-leucine ((*S*,*S*)-2)

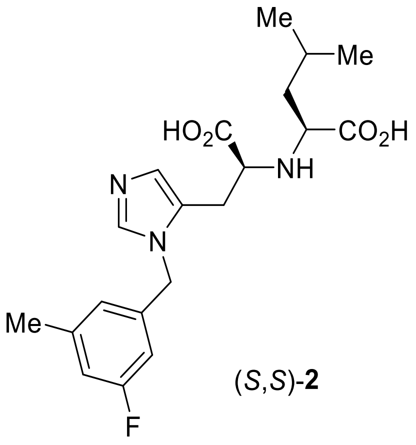

(*S*,*S*)-**2**

(*S*,*S*)-**15** (100 mg, 0.202 mmol, 1.0 equiv.) was subjected to hydrolysis using NaOH (24.2 mg, 0.606 mmol, 3.0 equiv.) in H_2O and MeOH (1:1, 0.404 mL, 0.5 M). The resulting mixture was stirred for 1 hour at room temperature, followed by the addition of aqueous HCl (1.0 M) to adjust the pH to 7.0. The mixture was stirred for an additional 30 minutes and then concentrated under reduced pressure and obtained the crude product (68.0 mg, 0.174 mmol, 86%, > 20:1 d.r.). The crude product was further purified using semi-preparative HPLC to obtain desired (*S*,*S*)-**2** as a white solid (35.0 mg, 89.4 μmol, 44%).

Reverse phase HPLC details: NUCLEODUR® RP-Säulen 5 μm C18 110 Å, 250 x 4.6 mm, flow rate = 15 mL/min, column temperature = 25 °C, eluent = gradient 2% - 55% MeCN/ H_2O (0.1% TFA) over 40 min, retention time = 17.9 min.

^{1}H NMR (500 MHz, D_2O) δ 8.77 (d, *J* = 1.7 Hz, 1H), 7.56 (d, *J* = 1.5 Hz, 1H), 7.09 – 7.01 (m, 1H), 6.98 – 6.95 (m, 1H), 6.92 – 6.86 (m, 1H), 5.45 – 5.41 (m, 2H), 3.78 – 3.68 (m, 2H), 3.31 – 3.29 (m, 1H), 3.29 – 3.27 (m, 1H), 2.35 (s, 3H), 1.86 – 1.78 (m, 1H), 1.77 – 1.68 (m, 2H), 0.97 – 0.93 (m, 6H).

^{13}C{^{1}H} NMR (126 MHz, D_2O) δ 173.7, 171.0, 162.8 (d, *J* = 246.1 Hz), 158.6, 135.6, 130.3 (d, *J* = 8.6 Hz), 128.9, 128.6 (d, *J* = 3.2 Hz), 119.2, 116.3, 116.2, 64.0, 61.9, 60.4, 49.9, 39.7, 24.8, 24.3, 21.6, 21.3.

^{19}F NMR (471 MHz, D_2O) δ -112.80 – -112.89 (m).

HRMS (ESI$^+$) m/z: $[M+H]^+$ Calcd for $C_{20}H_{27}FN_3O_4$ 392.1980; Found 392.1982.

IR (thin layer film) ν (cm^{-1}) 3657, 3343, 2980, 2972, 2887, 1770, 1636, 1471, 1463, 1380, 1252, 1160, 1130, 952, 817, 692.

MP 195 - 197 °C.

$[\alpha]_D^{25\,°C}$ +29.5 (c 0.070, H_2O).

Benzyl ((*S*)-3-(1-(3-chloro-5-fluorobenzyl)-1*H*-imidazol-5-yl)-1-methoxy-1-oxopropan-2-yl)-*L*-leucinate ((*S*,*S*)-16)

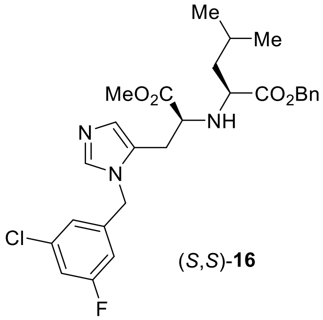

(*S*,*S*)-**16**

Di-*tert*-butyl dicarbonate (Boc_2O, 350 mg, 1.60 mmol, 2.2 equiv.) in CH_2Cl_2 (0.65 mL) was slowly added to a solution of (*S*,*S*)-**9** (300 mg, 0.802 mmol, 1.1 equiv.) and DIPEA (254 μL, 1.46 mmol, 2.0 equiv.) in CH_2Cl_2 (3.00 mL, 0.2 M) at room temperature. After stirring for 24 hours, the reaction mixture was concentrated *in vacuo*, and the residue was redissolved in CH_2Cl_2 and H_2O (1:1, 10 mL). After separation, the organic phase was washed with brine, dried over Na_2SO_4, filtered, and concentrated to obtain a crude intermediate. Separately, a solution of

trifluoromethanesulfonic anhydride (Tf_2O, 122 μL, 0.729 mmol, 1.0 equiv.) in CH_2Cl_2 (3.00 mL, 0.2 M) was cooled to -78 °C. To this solution, (3-chloro-5-fluorophenyl)methanol (**13**) (117 mg, 0.729 mmol, 1.0 equiv.) and diisopropylethylamine (DIPEA, 191 μL, 1.09 mmol, 1.5 equiv.) in CH_2Cl_2 (0.35 mL) were added. After stirring for 20 minutes, the crude intermediate obtained previously was dissolved in CH_2Cl_2 (0.30 mL) was added, and the reaction mixture was slowly brought to room temperature and stirred for 24 hours. Thereafter, HCl (0.201 mL, 4.0 M in dioxane, 1.1 equiv.) was added to the reaction mixture and stirred for 1 hour. The reaction mixture was then concentrated and the organic phase was extracted with EtOAc (3 × 25 mL), washed with brine (25 mL), dried over Na_2SO_4, and concentrated to yield the crude product. The crude product was further purified using flash column chromatography (gradient 0 - 10% MeOH in EtOAc) to obtain the product as a viscous cream oil (172 mg, 0.333 mmol, 46%, > 20:1 d.r.).

R_f 0.28 (5% MeOH/EtOAc).

^{1}H NMR (500 MHz, $(CD_3)_2CO$) δ 8.84 – 8.80 (m, 1H), 7.37 – 7.33 (m, 1H), 7.29 – 7.24 (m, 3H), 7.22 – 7.19 (m, 2H), 7.15 – 7.09 (m, 1H), 7.04 – 7.00 (m, 1H), 6.93 – 6.88 (m, 1H), 5.41 – 5.37 (m, 2H), 5.02 – 4.92 (m, 2H), 3.56 (s, 3H), 3.32 – 3.25 (m, 1H), 3.22 – 3.16 (m, 1H), 2.99 (dd, *J* = 15.6, 6.3 Hz, 1H), 2.86 (dd, *J* = 15.8, 7.9 Hz, 1H), 1.46 – 1.37 (m, 1H), 1.36 – 1.24 (m, 2H), 0.73 – 0.65 (m, 6H).

^{13}C{^{1}H} NMR (126 MHz, $(CD_3)_2CO$) δ 175.2, 173.9, 164.0 (d, *J* = 249.3 Hz), 140.2, 137.4, 137.0, 136.4 (d, *J* = 10.9 Hz), 132.2, 129.5, 129.2, 129.1, 125.0 (d, *J* = 3.6 Hz), 120.9, 117.1 (d, *J* = 25.0 Hz), 114.7 (d, *J* = 22.7 Hz), 67.1, 59.5, 59.2, 52.6, 49.9, 43.0, 28.1, 25.5, 23.3, 22.3.

^{19}F NMR (471 MHz, $(CD_3)_2CO$) δ -112.25 – -112.35 (m, 1F).

HRMS (ESI^+) m/z: $[M+H]^+$ Calcd for $C_{27}H_{32}ClFN_3O_4$ 516.2060; Found 516.2060.

IR (thin layer film) ν (cm^{-1}) 3457, 3067, 3040, 2957, 2871, 1737, 1677, 1512, 1439, 1388, 1282, 1260, 1226, 1181, 1113, 937, 807, 755, 663.

$[\alpha]_D^{25\,°C}$ -12.0 (c 0.010, $CHCl_3$).

((*S*)-1-Carboxy-2-(1-(3-chloro-5-fluorobenzyl)-1*H*-imidazol-5-yl)ethyl)-*L*-leucine ((*S,S*)-3)

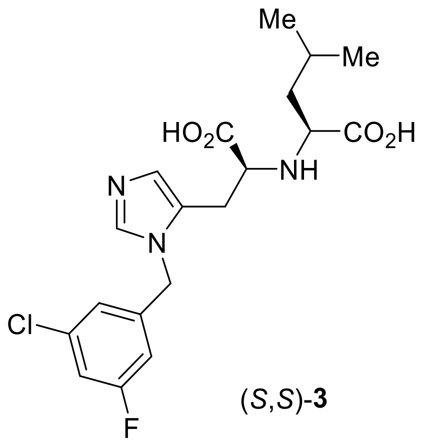

(*S*,*S*)-**3**

(*S,S*)-**16** (100 mg, 0.194 mmol, 1.0 equiv.) was subjected to hydrolysis using NaOH (23.3 mg, 0.581 mmol, 3.0 equiv.) in H_2O and MeOH (1:1, 0.388 mL, 0.5 M). The resulting mixture was stirred for 1 hour at room temperature, followed by the addition of aqueous HCl (1.0 M) to adjust the pH to 7.0. The mixture was stirred for an additional 30 minutes and then concentrated under reduced pressure and obtained the crude product (68.0 mg, 0.165 mmol, 85%, > 20:1 d.r.). The crude product was further purified using semi-preparative HPLC to obtain desired (*S*,*S*)-**3** as a white solid (35.0 mg, 85.0 μmol, 44%).

Reverse phase HPLC details: NUCLEODUR® RP-Säulen 5 μm C18 110 Å, 250 x 4.6 mm, flow rate = 15 mL/min, column temperature = 25 °C, eluent = gradient 2% - 55% MeCN/ H_2O (0.1% TFA) over 40 min, retention time = 16.0 min.

^{1}H NMR (500 MHz, D_2O) δ 8.77 (d, *J* = 1.7 Hz, 1H), 7.43 (d, *J* = 1.5 Hz, 1H), 7.25 – 7.18 (m, 1H), 7.09 – 7.05 (m, 1H), 6.97 – 6.91 (m, 1H), 5.43 (d, *J* = 16.2 Hz, 1H), 5.37 (d, *J* = 16.2 Hz, 1H), 3.69 – 3.62 (m, 2H), 3.21 – 3.16 (m, 2H), 1.71 – 1.63 (m, 1H), 1.63 – 1.56 (m, 1H), 1.50 – 1.41 (m, 1H), 0.82 (d, *J* = 6.6 Hz, 3H), 0.80 (d, *J* = 6.6 Hz, 3H).

^{13}C{^{1}H} NMR (126 MHz, D_2O) δ 173.1, 170.7, 163.8 (d, *J* = 245.1 Hz), 136.5 (d, *J* = 9.2 Hz), 136.0, 135.5 (d, *J* = 12.1 Hz), 129.0, 123.7, 119.4, 116.8 (d, *J* = 25.0 Hz), 113.5 (d, *J* = 22.7 Hz), 61.1, 60.1, 49.4, 39.5, 24.7, 24.3, 21.6, 21.3.

^{19}F NMR (471 MHz, D_2O) δ -110.03 – -110.15 (m, 1F).

HRMS (ESI$^+$) m/z: $[M+H]^+$ Calcd for $C_{19}H_{24}ClFN_3O_4$ 412.1434; Found 412.1435.

IR (thin layer film) ν (cm^{-1}) 3568, 3216, 3122, 2980, 2928, 2889, 2859, 1677, 1622, 1515, 1436, 1387, 1189, 1141, 938, 798, 723, 656.
MP 201–203 °C.
$[\alpha]_D^{25\,°C}$ +27.4 (c 0.025, H_2O).

Methyl 3-hydroxy-5-methoxybenzoate (S7)

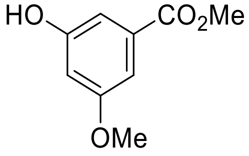

S7

A solution of methyl 3,5-dihydroxybenzoate (1.00 g, 5.97 mmol, 1.1 equiv.) in anhydrous DMF (7.00 mL) was added to a solution of sodium hydride (60% dispersion in mineral oil, 217 mg, 5.43 mmol, 1.0 equiv.) in DMF (20.0 mL) at 0˚C and stirred for 20 minutes. Therafter, iodomethane (0.372 mL, 5.97 mmol, 1.1 equiv.) was added dropwise over 10 minutes. The resulting mixture was slowly brought to room temperature and stirred for 1.5 hours, quenched with distilled H_2O (20 mL) at 0 ˚C and acidified with cold aqueous hydrochloric acid (1 M, 7.00 mL). After extraction with diethyl ether (3 × 25 mL), the combined organic phase was washed with brine (25 mL), dried over Na_2SO_4, and concentrated under reduced pressure. The crude product was further purified using flash column chromatography (10% EtOAc in pentane) to afford the desired product as a white solid (586 mg, 3.22 mmol, 59%).
$\mathbf{R_f}$ 0.35 (10% EtOAc/pentane).
^{1}H NMR (400 MHz, $CDCl_3$) δ 7.21 (dd, *J* = 2.3, 1.3 Hz, 1H), 7.12 (dd, *J* = 2.4, 1.4 Hz, 1H), 6.78 (br s, 1H), 6.64 (dd, *J* = 2.4, 2.4 Hz, 1H), 3.90 (s, 3H), 3.78 (s, 3H).
^{13}C{^{1}H} NMR (101 MHz, $CDCl_3$) δ 167.7, 160.9, 157.2, 131.8, 109.6, 107.0, 107.0, 55.7, 52.6.
Characterization data were in accordance with those found in the literature.[17]

3-(Hydroxymethyl)-5-methoxyphenol (S8)

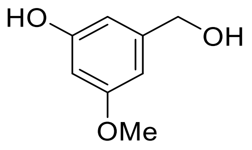

S8

Lithium aluminium hydride solution in dry THF (2.4 M, 11.4 mL, 27.5 mmol, 5.0 equiv.) was added dropwise to a solution of methyl 3-hydroxy-5-methoxybenzoate (**S7**) (1.00 g, 5.49 mmol, 1.0 equiv.) in dry tetrahydrofuran (THF, 54.9 mL) at 0˚C. After stirring for 10 minutes, the reaction mixture was slowly heated to reflux and stirred for overnight. After 16 hours, the reaction mixture was quenched by adding EtOAc (10 mL) and distilled H_2O (10 mL) dropwise at 0 ˚C, followed by the addition of aqueous HCl (1.0 M) to adjust the pH to 7.0. After concentration, the resulting residue was redissolved in EtOAc and H_2O (1:1, 30 mL). The aqueous layer was extracted with EtOAc (3 × 10 mL), and the combined organic layer was washed with brine (25 mL), dried over Na_2SO_4 and concentrated under reduced pressure. The crude product was further purified using flash column chromatography (30% EtOAc in pentane) to afford product **S8** as a yellow oil (721 mg, 4.68 mmol, 85%).

R_f 0.30 (30% EtOAc/pentane).

^{1}H NMR (500 MHz, $(CD_3)_2CO$) δ 8.39 (s, 1H), 6.49 (dd, *J* = 2.3, 1.4 Hz, 1H), 6.46 (dd, *J* = 2.3, 1.4 Hz, 1H), 6.31 (dd, *J* = 2.3, 2.3 Hz, 1H), 4.57 (br s, 2H), 3.71 (s, 3H).

^{13}C{^{1}H} NMR (126 MHz, $(CD_3)_2CO$) δ 161.8, 159.1, 145.3, 106. 8, 104.1, 100.7, 64.6, 55.3.

Characterization data were in accordance with those found in the literature.[18]

(3-(2-Fluoroethoxy)-5-methyoxyphenyl)methanol (14)

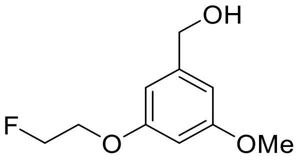

14

To a solution of **S8** (1.00 g, 6.49 mmol, 1.0 equiv.) dissolved in DMF (32.4 mL, 0.2 M) at room temperature, 2-fluoroethyl 4-methylbenzenesulfonate (1.42 g, 6.49 mmol, 1.0 equiv.) and caesium carbonate (4.23 g, 13.0 mmol, 2.0 equiv.) were added sequentially. After heating to 80 ˚C, the reaction mixture was stirred for 2 hours and subsequently quenched with distilled H_2O (30 mL) and EtOAc (30 mL) at room temperature, followed by neutralization with aqueous HCl (1.0 M). The concentrated mixture was then partitioned between equal volumes of EtOAc and H_2O, and the aqueous layer was further extracted with EtOAc (3 × 20 mL). The combined organic layer was washed with brine (25 mL), dried over Na_2SO_4 and concentrated under reduced pressure. The resulting residue was purified by flash column chromatography (50% EtOAc in pentane) to afford product **14** as a light brown solid (816 mg, 4.08 mmol, 63%).

R_f 0.32 (50% EtOAc/pentane).

^{1}H NMR (500 MHz, $CDCl_3$) δ 6.58 – 6.53 (m, 2H), 6.42 (dd, *J* = 2.4, 2.4 Hz, 1H), 4.75 (dt, *J* = 47.4, 4.0 Hz, 2H), 4.65 (d, *J* = 6.0 Hz, 2H), 4.21 (dt, *J* = 27.9, 4.1 Hz, 2H), 3.80 (s, 3H), 1.62 (t, *J* = 6.0 Hz, 1H).

^{13}C{^{1}H} NMR (126 MHz, $CDCl_3$) δ 161.2, 160.0, 143.6, 105.4, 105.1, 100.5, 82.0 (d, *J* = 170.8 Hz), 67.3 (d, *J* = 20.0 Hz), 65.5, 55.5.

^{19}F NMR (471 MHz, $CDCl_3$) δ -223.87 (tt, *J* = 46.8, 27.7 Hz).

HRMS (ESI^+) m/z: $[M+H]^+$ Calcd for $C_{10}H_{14}FO_3$ 201.0921; Found 201.0919.

IR (thin layer film) ν (cm^{-1}) 3391, 2997, 2954, 2873, 2842, 1599, 1469, 1438, 1348, 1322, 1296, 1238, 1198, 1165, 1053, 930, 886, 836, 688.

MP 44–45 °C.

Benzyl ((S)-3-(1-(3-(2-fluoroethoxy)-5-methoxybenzyl)-1*H*-imidazol-5-yl)-1-methoxy-1-oxopropan-2-yl)-*L*-leucinate ((*S*,*S*)-17)

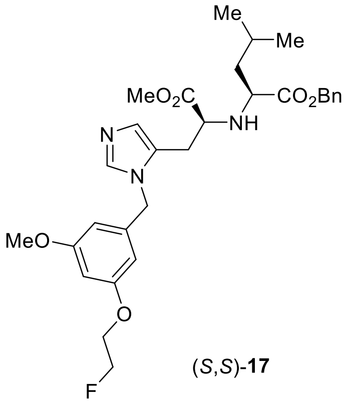

(*S*,*S*)-**17**

Di-*tert*-butyl dicarbonate (Boc$_2$O, 350 mg, 1.60 mmol, 2.2 equiv.) in CH_2Cl_2 (0.65 mL) was slowly added to a solution of (*S*,*S*)-**9** (300 mg, 0.802 mmol, 1.1 equiv.) and DIPEA (254 μL, 1.46 mmol, 2.0 equiv.) in CH_2Cl_2 (3.00 mL, 0.2 M) at room temperature. After stirring for 24 hours, the reaction mixture was concentrated under reduced pressure, and the residue was redissolved in CH_2Cl_2 and H_2O (1:1, 10 mL). After separation, and the organic phase was washed with brine (25 mL), dried over Na_2SO_4, filtered, and concentrated to obtain a crude intermediate. Separately, a solution of trifluoromethanesulfonic anhydride (Tf$_2$O, 122 μL, 0.729 mmol, 1.0 equiv.) in CH_2Cl_2 (3.00 mL, 0.2 M) was cooled to -78 °C. To this solution, **14** (146 mg, 0.729 mmol, 1.0 equiv.) and diisopropylethylamine (DIPEA, 191 μL, 1.09 mmol, 1.5 equiv.) in CH_2Cl_2 (0.35 mL) were added. After stirring for 20 minutes, a solution of the previously obtained crude intermediate in CH_2Cl_2 (0.30 mL) was added, and the reaction mixture was slowly brought to room temperature and stirred for 24 hours. After that, HCl (0.201 mL, 4.0 M in dioxane, 1.1 equiv.) was added to the reaction mixture and stirred for 1 hour. The reaction mixture was then concentrated and the organic phase was extracted with EtOAc (3 × 25 mL), washed with brine (25 mL), dried over Na_2SO_4, and concentrated to yield the crude product. The crude product was further purified using flash column chromatography (gradient 50 - 0% pentane in EtOAc) to obtain the product as a viscous cream oil (165 mg, 0.297 mmol, 41%, > 20:1 *d.r.*).

R$_f$ 0.33 (10% pentane/EtOAc).

^{1}H NMR (500 MHz, $CDCl_3$) δ 7.53 – 7.49 (m, 1H), 7.41 – 7.28 (m, 5H), 6.83 – 6.77 (m, 1H), 6.58 – 6.53 (m, 2H), 6.42 (dd, *J* = 2.4, 2.4 Hz, 1H), 5.15 – 5.11 (m, 2H), 4.81 – 4.67 (m, 2H), 4.64 (s, 3H), 4.26 – 4.15 (m, 2H), 3.79 (s, 3H), 3.74 – 3.70 (m, 2H), 3.48 (dd, *J* = 8.9, 3.5 Hz, 1H), 3.42

(dd, J = 8.1, 5.8 Hz, 1H), 2.99 (dd, J = 15.4, 3.5 Hz, 1H), 2.81 (ddd, J = 15.6, 8.9, 1.1 Hz, 1H), 1.78 – 1.67 (m, 1H), 1.65 – 1.52 (m, 2H), 0.93 (d, J = 6.6 Hz, 3H), 0.92 (d, J = 6.6 Hz, 3H).

^{13}C{^{1}H} NMR (126 MHz, $CDCl_3$) δ 175.6, 174.0, 161.2, 159.9, 143.7, 135.5, 134.7, 128.8, 128.7 (d, J = 2.3 Hz), 128.5, 127.8, 127.1, 105.4, 105.1, 100.5, 82.0 (d, J = 170.8 Hz), 67.3 (d, J = 20.4 Hz), 67.2, 65.4, 59.8, 58.9, 55.5, 52.4, 42.9, 29.2, 25.0, 23.0, 22.1.

^{19}F NMR (471 MHz, $CDCl_3$) δ -224.23 (tt, J = 46.8, 27.7 Hz, 1F).

HRMS (ESI^+) m/z: $[M+H]^+$ Calcd for $C_{30}H_{39}FN_3O_6$ 556.2817; Found 556.2817.

IR (thin layer film) ν (cm^{-1}) 3349, 3128, 3065, 3038, 2965, 2939, 2877, 1740, 1679, 1611, 1593, 1449, 1275, 1205, 1140, 954, 867, 801, 752, 722, 700.

$[\alpha]_D^{25\ °C}$ -14.2 (c 0.010, $CHCl_3$).

(*(S)*-1-Carboxy-2-(1-(3-(2-fluoroethoxy)-5-methoxybenzyl)-1*H*-imidazol-5-yl)ethyl)-*L*-leucine ((*S,S*)-4)

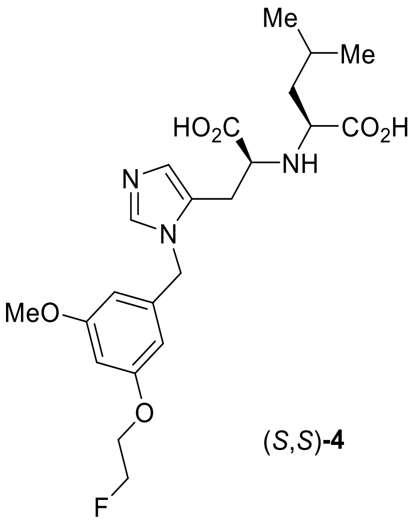

(*S*,*S*)-**4**

The protected (*S,S*)-**17** (100 mg, 0.180 mmol, 1.0 equiv.) was subjected to hydrolysis using NaOH (21.6 mg, 0.540 mmol, 3.0 equiv.) in H_2O and MeOH (1:1, 0.415 mL, 0.5 M). The resulting mixture was stirred for 1 hour at room temperature, followed by the addition of HCl (1.0 M) to adjust the pH to 7.0. The mixture was stirred for an additional 30 minutes and then concentrated under reduced pressure and obtained the crude product (67.0 mg, 0.148 mmol, 82%, > 20:1 *d.r.*). The crude product was further purified using semi-preparative HPLC to obtain desired (*S,S*)-**1** as

a white solid (40.0 mg, 88.6 μmol, 49%). **Reverse phase HPLC details**: NUCLEODUR® RP-Säulen 5 μm C18 110 Å, 250 x 4.6 mm, flow rate = 15 mL/min, column temperature = 25 °C, eluent = gradient 2% - 55% MeCN/ H_2O (0.1% TFA) over 40 min, retention time = 24.0 min.

^{1}H NMR (500 MHz, D_2O) δ 8.75 (d, *J* = 1.5 Hz, 1H), 7.55 (d, *J* = 1.5 Hz, 1H), 6.67 (dd, *J* = 2.3, 2.3 Hz, 1H), 6.60 – 6.43 (m, 2H), 5.41 – 5.38 (m, 2H), 4.87 – 4.82 (m, 1H), 4.77 – 4.72 (m, 1H), 4.36 – 4.31 (m, 1H), 4.30 – 4.25 (m, 1H), 3.81 (s, 3H), 3.77 (dd, *J* = 7.2, 7.2 Hz, 1H), 3.71 (dd, *J* = 7.5, 6.1 Hz, 1H), 3.32 – 3.27 (m, 2H), 1.84 – 1.77 (m, 1H), 1.76 – 1.68 (m, 2H), 0.96 – 0.90 (m, 6H).

^{13}C{^{1}H} NMR (126 MHz, D_2O) δ 163.1, 162.8, 161.0, 159.8, 135.8, 135.5, 128.7, 119.4, 117.5, 115.2, 114.0, 106.9 (d, *J* = 6.8 Hz), 101.4, 82.6 (d, *J* = 164.4 Hz), 67.7 (d, *J* = 18.6 Hz), 55.6, 50.3, 39.2, 24.6, 24.3, 21.4, 21.2.

^{19}F NMR (471 MHz, D_2O) δ -223.30 – -223.49 (m, 1F).

HRMS (ESI^+) m/z: $[M+H]^+$ Calcd for $C_{22}H_{31}FN_3O_6$ 452.2191; Found 452.2192.

IR (thin layer film) ν (cm^{-1}) 3356, 3125, 2965, 2936, 1618, 1592, 1447, 1387, 1347, 1270, 1099, 1047, 986, 855, 670, 637.

MP 186–188 °C.

$[\alpha]_D^{25\ °C}$ +24.5 (c 0.015, H_2O).

Preparation of radiofluorination precursor ((*S*,*S*)-18)

Scheme S2.4. Two-step synthesis of (*S*,*S*)-**18** in an N3-selective benzylation, Miyaura borylation sequence.

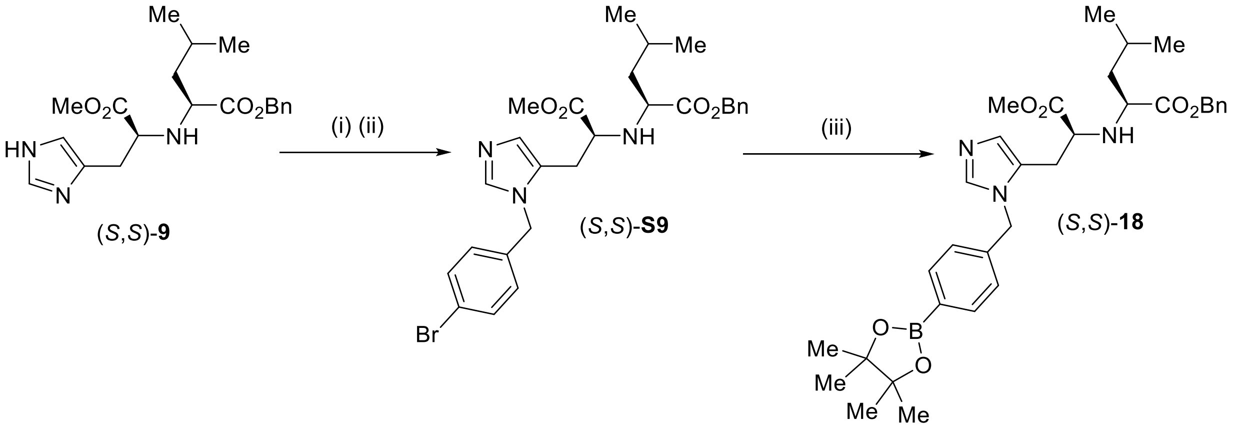

(i) (*S*,*S*)-**9** (1.1 equiv.), Boc_2O (2.2 equiv.), DIPEA (2.0 equiv.), CH_2Cl_2, rt, 24 h, isolated as crude, then (ii) $(CF_3SO_2)_2O$ (1.0 equiv.), (4-bromophenyl)methanol (1.0 equiv.), DIPEA (1.5 equiv.), -78 °C to rt, 24 h, then HCl (4.0 M in dioxane, 1.1 equiv.), rt, 1 h; (iii) (*S*,*S*)-**S9** (1.0 equiv.), bis(pinacolato)diboron (2.2 equiv.), $Pd(OAc)_2$ (2 mol%), XPhos (4 mol%), KOAc (3.0 equiv.), 1,4-dioxane (0.1 M), 110 °C, 20 min.

Benzyl ((*S*)-3-(1-(4-bromobenzyl)-1*H*-imidazol-5-yl)-1-methoxy-1-oxopropan-2-yl)-*L*-leucinate ((*S*,*S*)-S9)

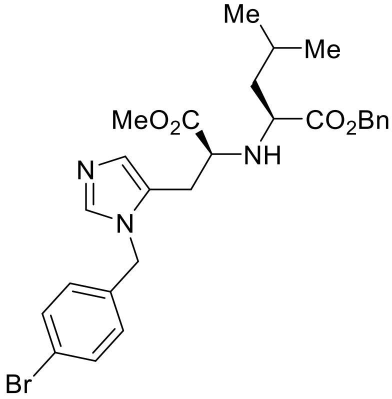

Di-*tert*-butyl dicarbonate (Boc_2O, 350 mg, 1.60 mmol, 2.2 equiv.) in CH_2Cl_2 (0.65 mL) was slowly added to a solution of (*S*,*S*)-**9** (300 mg, 0.802 mmol, 1.1 equiv.) and DIPEA (254 µL, 1.46 mmol,

2.0 equiv.) in CH_2Cl_2 (3.00 mL, 0.2 M) at room temperature. After stirring for 24 hours, the reaction mixture was concentrated under reduced pressure, and the residue was redissolved in dichloromethane (CH_2Cl_2) and H_2O (1:1, 10 mL). After separation, and the organic phase was washed with brine, dried over Na_2SO_4, filtered, and concentrated to obtain a crude intermediate. Separately, a solution of trifluoromethanesulfonic anhydride (Tf_2O, 122 µL, 0.729 mmol, 1.0 equiv.) in CH_2Cl_2 (3.00 mL, 0.2 M) was cooled to -78 °C. To this solution, (4-bromophenyl)methanol (136 mg, 0.729 mmol, 1.0 equiv.) and diisopropylethylamine (DIPEA, 191 µL, 1.09 mmol, 1.5 equiv.) in CH_2Cl_2 (0.35 mL) were added. After stirring for 20 minutes, to this solution was added a solution of the crude intermediate in CH_2Cl_2 (0.30 mL) was added, and the reaction mixture was slowly brought to room temperature and stirred overnight. Thereafter, HCl (0.201 mL, 4.0 M in dioxane, 1.1 equiv.) was added to the reaction mixture and stirred for 1 hour. The reaction mixture was then concentrated and the organic phase was extracted with EtOAc (3 × 25 mL), washed with brine (25 mL), dried over Na_2SO_4, and concentrated to yield the crude product. The crude product was further purified using flash column chromatography (gradient 0 - 10% MeOH in EtOAc) to obtain the product as a viscous cream oil (173 mg, 0.319 mmol, 44%, > 20:1 *d.r.*).

R_f 0.35 (2% MeOH/EtOAc).

^{1}H NMR (500 MHz, $CDCl_3$) δ 8.66 – 8.63 (m, 1H), 7.57 – 7.55 (m, 1H), 7.55 – 7.53 (m, 1H), 7.40 – 7.30 (m, 6H), 7.08 – 7.06 (m, 1H), 7.06 – 7.03 (m, 1H), 5.38 – 5.29 (m, 2H), 5.16 – 5.09 (m, 2H), 3.74 (s, 3H), 3.52 (dd, *J* = 6.9, 5.6 Hz, 1H), 3.47 (dd, *J* = 8.2, 5.5 Hz, 1H), 2.99 (dd, *J* = 15.6, 5.8 Hz, 1H), 2.87 (dd, *J* = 15.7, 6.7 Hz, 1H), 1.70 – 1.61 (m, 1H), 1.57 (ddd, *J* = 13.7, 8.2, 5.6 Hz, 1H), 1.46 (ddd, *J* = 14.0, 8.3, 6.1 Hz, 1H), 0.91 (d, *J* = 6.6 Hz, 3H), 0.89 (d, *J* = 6.6 Hz, 3H).

^{13}C{^{1}H} NMR (126 MHz, $CDCl_3$) δ 174.1, 172.6, 135.6, 135.4, 133.0, 131.5, 130.7, 129.4, 128.9, 128.8, 128.5, 124.0, 119.8, 67.2, 59.1, 59.0, 53.0, 50.6, 42.1, 27.6, 25.0, 22.9, 21.9.

HRMS (ESI^+) m/z: $[M+H]^+$ Calcd for $C_{27}H_{33}BrN_3O_4$ 542.1649; Found 542.1648.

IR (thin layer film) ν (cm^{-1}) 3389, 2984, 2951, 2684, 1640, 1541, 1475, 1397, 1222, 1025, 838, 670.

$[\alpha]_D^{25\ °C}$ -16.5 (c 0.015, $CHCl_3$).

Benzyl ((*S*)-1-methoxy-1-oxo-3-(1-(4-(4,4,5,5-tetramethyl-1,3,2-dioxaborolan-2-yl)benzyl)-1*H*-imidazol-5-yl)propan-2-yl)-*L*-leucinate ((*S*,*S*)-18)

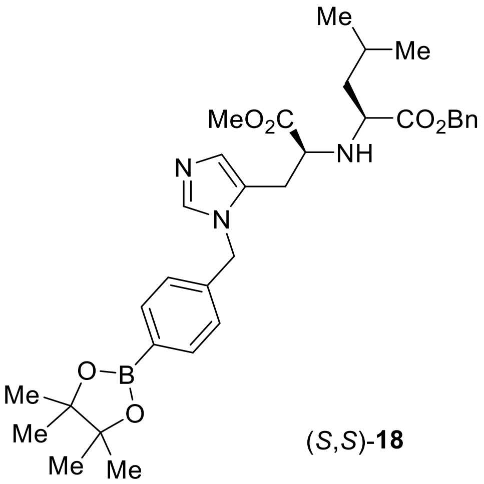

(*S*,*S*)-**18**

An oven-dried Schlenk tube was charged with (*S*,*S*)-**S9** (80 mg, 148 µmol, 1.0 equiv.), bis(pinacolato)diboron (82.4 mg, 324 µmol, 2.2 equiv.), $Pd(OAc)_2$ (0.662 mg, 2.95 µmol, 2 mol%), XPhos (2.81 mg, 5.90 µmol, 4 mol%), and potassium acetate (43.4 mg, 442 µmol, 3.0 equiv.). The tube was sealed with a rubber septum, evacuated, and backfilled with argon (repeated twice). Degassed 1,4-dioxane (1.47 mL, 0.1 M) was added via syringe, and the reaction mixture was stirred at 110 °C for 20 minutes. The mixture was then cooled to room temperature, filtered through a pad of celite, and concentrated under reduced pressure. The organic phase was extracted with EtOAc (3 × 10 mL), washed with brine (10 mL), dried over Na_2SO_4, and purified using flash column chromatography (gradient 0–5% MeOH in EtOAc) to afford the crude product (62.0 mg, 105 µmol, 71% yield). The crude product was further purified using preparative HPLC to obtain the product as a viscous colourless oil (26.0 mg, 44.1 µmol, 30%).

Reverse phase HPLC details: NUCLEODUR® RP-Säulen 5 µm C18 110 Å, 250 x 4.6 mm, flow rate = 12 mL/min, column temperature = 25 °C, eluent = gradient 50% - 98% MeCN/ H_2O (0.1% TFA) over 40 min, retention time = 31.8 min;

^{1}H NMR (500 MHz, $(CD_3)_2CO$) δ 8.84 – 8.77 (m, 1H), 7.48 – 7.29 (m, 8H), 7.22 – 7.14 (m, 2H), 5.67 – 5.62 (m, 2H), 5.16 – 5.11 (m, 2H), 3.68 (s, 3H), 3.62 (dd, *J* = 7.7, 5.3 Hz, 1H), 3.48 (dd, *J* = 8.2, 6.0 Hz, 1H), 3.15 – 3.08 (m, 1H), 3.02 – 2.93 (m, 1H), 1.75 – 1.65 (m, 1H), 1.55 – 1.41 (m, 2H), 1.21 (s, 12H), 0.90 – 0.84 (m, 6H).

$^{13}C\{^1H\}$ NMR (126 MHz, $(CD_3)_2CO$) δ 175.0, 173.8, 164.6, 162.7, 137.2, 131.1, 131.0, 129.4, 129.1, 129.0, 116.9, 116.7, 83.7, 66.9, 59.4, 59.3, 59.0, 52.4, 50.3, 42.9, 25.4, 24.9, 23.2, 22.2.
HRMS (ESI^+) m/z: $[M+H]^+$ Calcd for $C_{33}H_{45}BN_3O_6$ 590.3396; Found 590.3391.
IR (thin layer film) ν (cm^{-1}) 3348, 2971, 2932, 2884, 1739, 1675, 1467, 1379, 1341, 1306, 1161, 1129, 952, 817, 722, 698, 650, 618.
$[\alpha]_D^{25\,°C}$ -21.8 (c 0.010, $CHCl_3$).

Scheme S2.5. Two-step synthesis of (*S*,*S*)-**19** in an N3-selective benzylation, Miyaura borylation sequence.

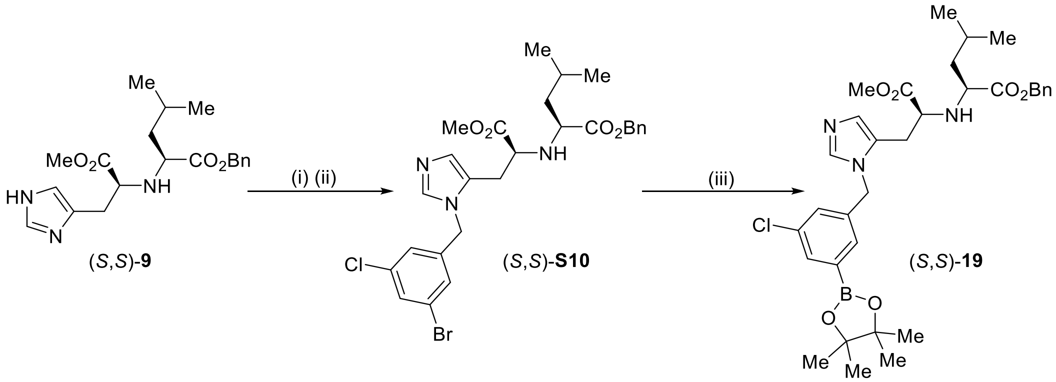

(i) (*S,S*)-**9** (1.1 equiv.), Boc_2O (2.2 equiv.), DIPEA (2.0 equiv.), CH_2Cl_2, rt, 24 h, isolated as crude, then (ii) $(CF_3SO_2)_2O$ (1.0 equiv.), (3-bromo-5-chlorophenyl)methanol (1.0 equiv.), DIPEA (1.5 equiv.), -78 °C to rt, 24 h, then HCl (4.0 M in dioxane, 1.1 equiv.), rt, 1 h; (iii) (*S,S*)-**S10** (1.0 equiv.), bis(pinacolato)diboron (2.2 equiv.), Pd(dppf)Cl_2 (2 mol%), KOAc (3.0 equiv.), 1,4-dioxane (0.1 M), 110 °C, 30 min.

Benzyl ((*S*)- 3-(1-(3-bromo-5-chlorobenzyl)-1*H*-imidazol-5-yl)-1-methoxy-1-oxopropan-2-yl)-*L*-leucinate ((*S*,*S*)-S10)

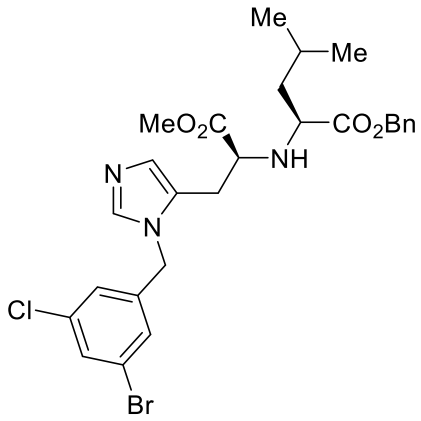

Di-*tert*-butyl dicarbonate (Boc_2O) (350 mg, 1.60 mmol, 2.2 equiv.) in CH_2Cl_2 (0.65 mL) was slowly added to a solution of (*S*,*S*)-**9** (300 mg, 0.802 mmol, 1.1 equiv.) and DIPEA (254 μL, 1.46 mmol, 2.0 equiv.) in CH_2Cl_2 (3.00 mL, 0.2 M) at room temperature. After stirring for 24 hours, the reaction mixture was concentrated under reduced pressure, and the residue was redissolved in dichloromethane (CH_2Cl_2) and H_2O (1:1, 10 mL). After separation, the organic phase was washed with brine, dried over Na_2SO_4, filtered, and concentrated to obtain a crude intermediate. Separately, a solution of trifluoromethanesulfonic anhydride (Tf_2O, 122 μL, 0.729 mmol, 1.0 equiv.) in CH_2Cl_2 (3.00 mL, 0.2 M) was cooled to -78 °C. To this solution, (3-bromo-5-chlorophenyl)methanol (162 mg, 0.729 mmol, 1.0 equiv.) and diisopropylethylamine (DIPEA, 191 μL, 1.09 mmol, 1.5 equiv.) in CH_2Cl_2 (0.35 mL) were added. After stirring for 20 minutes, to this solution was added a solution of the crude intermediate in CH_2Cl_2 (0.30 mL), and the reaction mixture was slowly brought to room temperature and stirred overnight. Thereafter, HCl (0.201 mL, 4.0 M in dioxane, 1.1 equiv.) was added to the reaction mixture and stirred for 1 hour. The reaction mixture was then concentrated and the organic phase was extracted with EtOAc (3 × 25 mL), washed with brine (25 mL), dried over Na_2SO_4, and concentrated to yield the crude product. The crude product was further purified using flash column chromatography (gradient 0 - 10% MeOH in EtOAc) to obtain the product as a viscous cream oil (170 mg, 0.295 mmol, 40%, > 20:1 *d.r.*).

R_f 0.30 (2% MeOH/EtOAc).

^{1}H NMR (500 MHz, $CDCl_3$) δ 7.45 – 7.42 (m, 2H), 7.37 – 7.30 (m, 5H), 7.06 – 7.03 (m, 1H), 6.93 – 6.91 (m, 1H), 6.90 – 6.88 (m, 1H), 5.12 – 5.09 (m, 2H), 5.06 – 5.04 (m, 2H), 3.67 (s, 3H), 3.43 – 3.39 (m, 1H), 3.35 (dd, *J* = 8.3, 5.9 Hz, 1H), 2.82 (ddd, *J* = 15.1, 6.7, 0.8 Hz, 1H), 2.74

(ddd, J = 15.1, 6.6, 0.8 Hz, 1H), 1.69 – 1.62 (m, 1H), 1.51 – 1.44 (m, 1H), 1.44 – 1.36 (m, 1H), 0.90 – 0.83 (m, 6H).

^{13}C{^{1}H} NMR (126 MHz, $CDCl_3$) δ 174.7, 173.7, 140.2, 138.3, 136.0, 135.8, 131.2, 129.2, 128.8, 128.5, 128.4, 128.1, 127.2, 125.7, 123.6, 66.7, 59.4, 58.5, 52.3, 47.4, 42.4, 28.3, 24.9, 23.0, 22.0.

HRMS (ESI^+) m/z: $[M+H]^+$ Calcd for $C_{27}H_{32}BrClN_3O_4$ 576.1259; Found 576.1249.

IR (thin layer film) ν (cm^{-1}) 3422, 2957, 1736, 1710, 1591, 1565, 1496, 1432, 1366, 1222, 1153, 1112, 971, 753, 699, 668.

$[\alpha]_D^{25\ °C}$ -12.1 (c 0.020, $CHCl_3$).

Benzyl ((*S*)-3-(1-(3-chloro-5-(4,4,5,5-tetramethyl-1,3,2-dioxaborolan-2-yl)benzyl)-1*H*-imidazol-5-yl)-1-methoxy-1-oxopropan-2-yl)-*L*-leucinate ((*S*,*S*)-19)

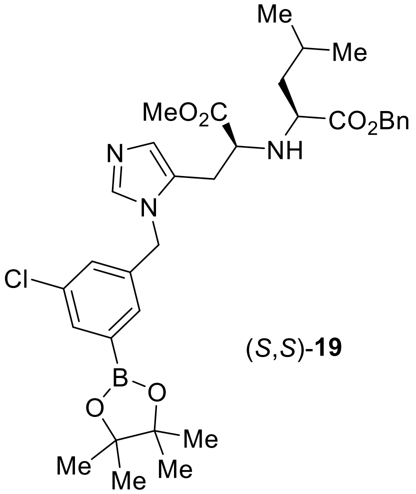

(*S*,*S*)-**19**

An oven-dried Schlenk tube was charged with (*S*,*S*)-**S10** (50 mg, 86.7 μmol, 1.0 equiv.), bis(pinacolato)diboron (48.4 mg, 191 μmol, 2.2 equiv.), (1,1'-bis(diphenylphosphino)ferrocene)palladium(II) dichloride (Pd(dppf)Cl_2, 1.27 mg, 1.73 μmol, 2 mol%), and potassium acetate (25.5 mg, 260 μmol, 3.0 equiv.). The tube was sealed with a rubber septum, evacuated, and backfilled with argon (repeated twice). Degassed 1,4-dioxane (867 μL, 0.1 M) was added via syringe, and the reaction mixture was stirred at 110 °C for 30 minutes. The mixture was then cooled to room temperature, filtered through a pad of celite, and concentrated under reduced pressure. The organic phase was extracted with EtOAc (3 × 10 mL), washed with brine (10 mL), dried over Na_2SO_4, and purified using flash column chromatography (gradient 0–

5% MeOH in EtOAc) to afford the product as a viscous colorless oil (36.0 mg, 57.7 μmol, 67% yield).

^{1}H NMR (500 MHz, $(CD_3)_2CO$) δ 7.65 – 7.62 (m, 1H), 7.58 – 7.55 (m, 1H), 7.41 – 7.34 (m, 5H), 7.27 – 7.25 (m, 1H), 7.16 – 7.14 (m, 1H), 6.84 – 6.82 (m, 1H), 5.37 – 5.33 (m, 2H), 5.15 – 5.11 (m, 2H), 3.65 (s, 3H), 3.53 – 3.49 (m, 1H), 3.41 (dd, *J* = 8.4, 6.0 Hz, 1H), 2.91 (ddd, *J* = 15.3, 6.6, 0.9 Hz, 1H), 2.79 (ddd, *J* = 15.3, 6.7, 0.9 Hz, 1H), 1.77 – 1.66 (m, 1H), 1.48 – 1.37 (m, 2H), 1.23 (s, 12H), 0.89 – 0.84 (m, 6H).

^{13}C{^{1}H} NMR (126 MHz, $(CD_3)_2CO$) δ 175.1, 174.2, 143.2, 139.1, 137.3, 136.1, 131.1, 129.3, 129.3, 129.0, 129.0, 128.3, 126.8, 123.7, 83.7, 66.8, 60.0, 58.9, 52.1, 47.5, 43.0, 28.5, 25.4, 24.9, 23.2, 22.2.

HRMS (ESI^+) m/z: $[M+H]^+$ Calcd for $C_{33}H_{44}BClN_3O_6$ 624.3006; Found 624.3016.

IR (thin layer film) ν (cm^{-1}) 3355, 2972, 1731, 1468, 1378, 1303, 1253, 1162, 1131, 1048, 953, 817, 620.

$[\alpha]_D^{25\ °C}$ -14.6 (c 0.010, $CHCl_3$).

3. Radiochemistry

3.1 General Information

[^{18}F]Fluoride was produced in an IBA Cyclon 18/9 cyclotron using the $^{18}O(p,n)^{18}F$ reaction in PETIC (UK). Analytical HPLC runs were performed with an Agilent 1200 equipped with a UV detector and LabLogic gamma-RAM Model 4 detector (approximate radio-UV detector offset = 0.1 min).

Conditions A:

Flow rate = 1.0 mL/min

Temperature = 25 °C

Wavelength = 220 nm (unless otherwise specified)

HPLC gradient: water/MeCN or water + 0.1% TFA/MeCN + 0.1% TFA

0-1 min (5% MeCN) isocratic

1-10 min (5% MeCN to 95% MeCN) linear increase

10-16 min (95% MeCN) isocratic

16-18 min (95% MeCN to 5% MeCN) linear decrease

18-20 min (5% MeCN) isocratic

Conditions B:

Flow rate = 1.0 mL/min

Temperature = 25 °C

Wavelength = 220 nm (unless otherwise specified)

HPLC gradient: water/MeCN or water + 0.1% TFA/MeCN + 0.1% TFA

0-1 min (25% MeCN) isocratic

1-10 min (25% MeCN to 95% MeCN) linear increase

10-16 min (95% MeCN) isocratic

16-18 min (95% MeCN to 25% MeCN) linear decrease

18-20 min (25% MeCN) isocratic

3.2 Radiochemistry protocols

Preparation of [^{18}F]Fluoride

The following procedure was performed using a Trasis AllinOne automated synthesizer. [^{18}F]Fluoride was first separated from [^{18}O]water using an anion exchange cartridge (Waters Sep-Pak AccellPlus QMA Carbonate Plus Light Cartridge), activated with H_2O (10 mL) prior to use and released with a solution of Kryptofix (7.5 mg), and K_2CO_3 (1.5 mg) in MeCN/H_2O (0.75 mL, 4:1, v/v). The eluate was transferred to the reactor and dried azeotropically with additional MeCN at 110 °C. The dry residue was redissolved in MeCN and transferred into a 3 mL syringe for dispensing.

Cu-mediated ^{18}F-Fluorination

To an oven-dried 3 mL v-vial equipped with a magnetic stir bar was added the relevant Bpin precursor, Cu(OTf)$_2$py$_4$ (2 equiv.) and DMI (300 µL). To the vial was then added an aliquot of the [^{18}F]KF.K$_{222}$ solution (5-20 MBq) in MeCN (approx. 20 µL) (*vide supra*). The reaction vial was purged with air (20 mL) and reaction mixture was stirred at the given temperature for 20 minutes. The reaction mixture was cooled to rt. An aliquot of this mixture was subsequently analyzed by radioHPLC (gradient: MeCN/H_2O) for radiochemical yield (RCY) and product identity.

Scheme S3.1. Copper-mediated radiofluorination of pinacol boronic ester (*S*,*S*)-**18**.

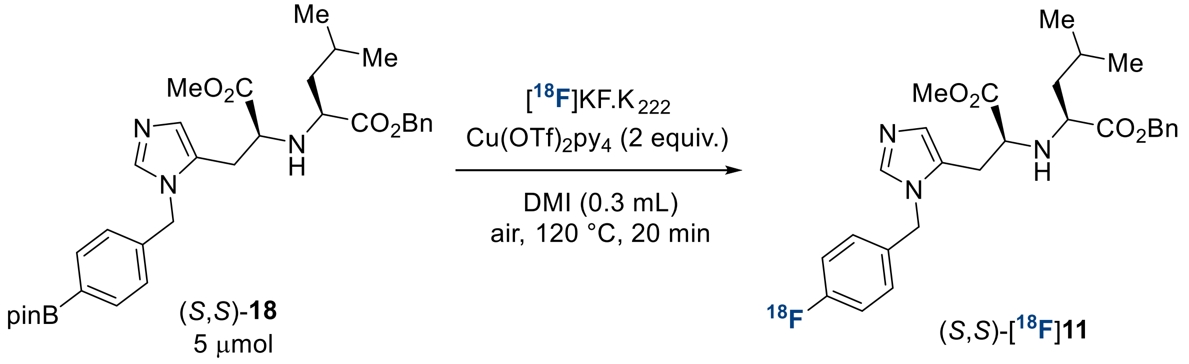

RCY$_{HPLC}$: radiochemical yield, determined by radio-HPLC analysis (Conditions A, column: Phenomenex Kinetex C18 250 x 4.6 mm) of the crude reaction mixture. Product identity confirmed by comparison of retention time to an authentic reference standard ((*S*,*S*)-**11**) by UV-HPLC.

Radio/UV-HPLC trace overlay:

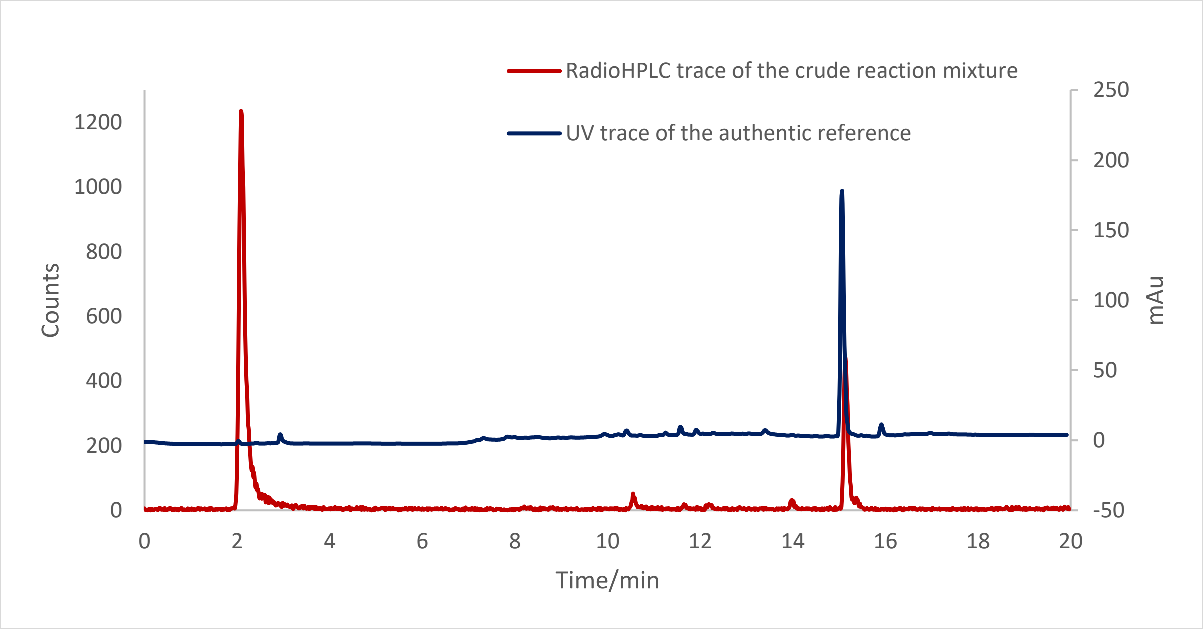

Entry	RCY$_{HPLC}$ of (S,S)-[^{18}F]**11**
1	31%
2	17%
Average	24% ± 7% (*n* = 2)

Scheme S3.2. Copper-mediated radiofluorination of pinacol boronic ester (*S*,*S*)-**19**.

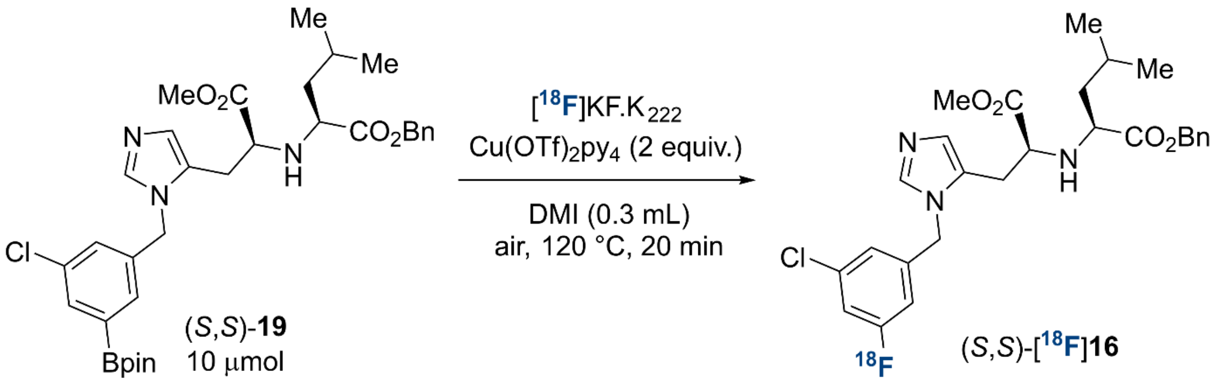

RCY$_{HPLC}$: radiochemical yield, determined by radio-HPLC analysis (Conditions B, column: Agilent C18 Eclipse Plus 80 Å 150 x 4.6 mm) of the crude reaction mixture. Product identity confirmed by comparison of retention time to an authentic reference standard ((*S*,*S*)-**16**) by UV-HPLC.

Radio/UV-HPLC trace overlay:

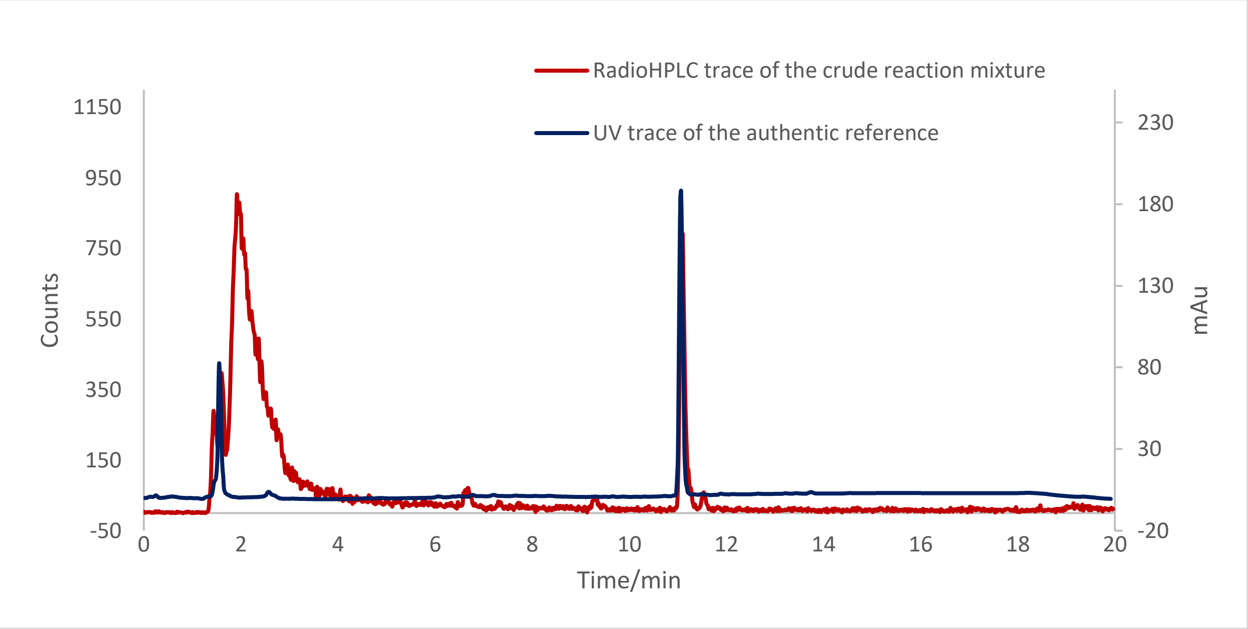

Entry	RCY$_{HPLC}$ of (S,S)-[^{18}F]**16**
1	12%
2	6%
Average	9% ± 3% (*n* = 2)

Radiosynthesis of (*S*,*S*)-[^{18}F]1

<u>*Procedure:*</u> To an oven-dried 3 mL v-vial equipped with a magnetic stir bar was added (*S*,*S*)-[^{18}F]**18** (5 μmol), $Cu(OTf)_2py_4$ (2 equiv.) and DMI (300 μL). To the vial was then added an aliquot of the [^{18}F]KF.K_{222} solution (5-20 MBq) in MeCN (approx. 20 μL) (*vide supra*). The reaction vial was purged with air (20 mL) and reaction mixture was stirred at 120 °C for 20 minutes. The reaction mixture was cooled to rt and NaOH (aq., 1 M, 0.1 mL) was added. This mixture was heated at 65 °C for 10 minutes. Once cooled, the reaction mixture was neutralised with HCl (aq., 1 M, 0.1 mL). An aliquot of this mixture was subsequently analysed by radioHPLC for radiochemical yield (RCY) and product identity.

Scheme S3.3. Two-step radiosynthesis of (*S*,*S*)-[^{18}F]**1**

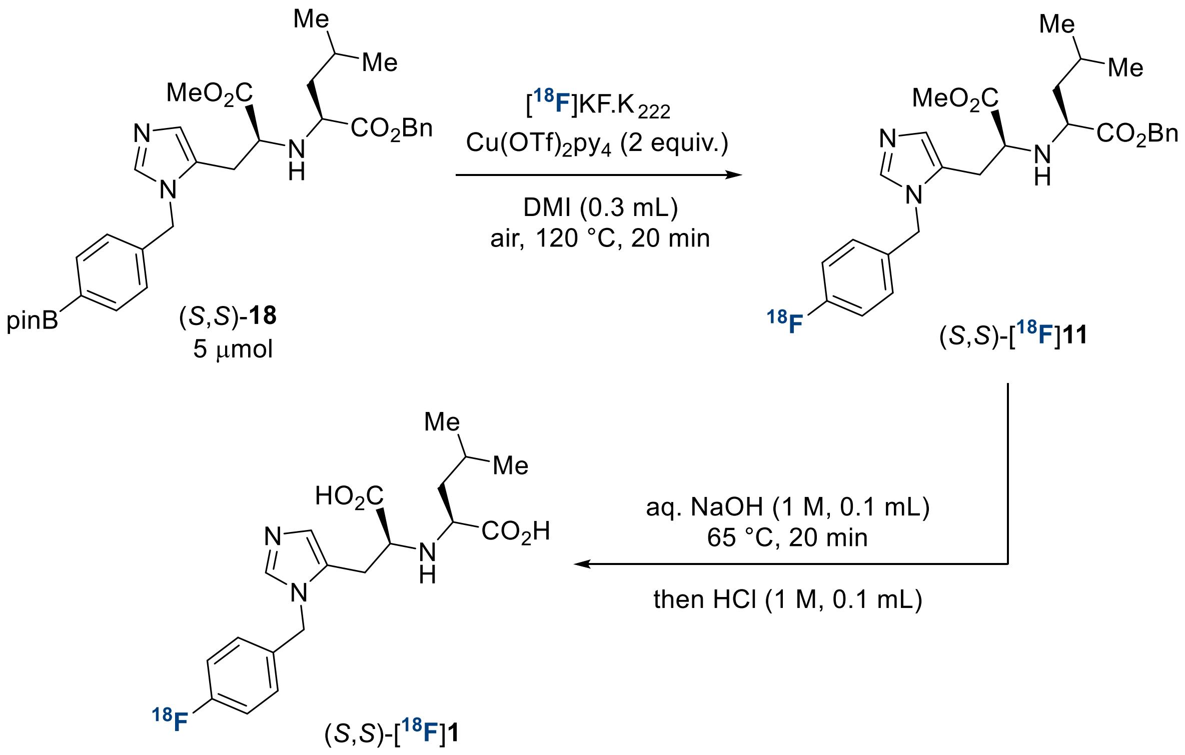

RCY_{HPLC}: radiochemical yield, determined by radio-HPLC analysis (Conditions A, Phenomenex Kinetex C18 250 x 4.6 mm, gradient: MeCN +0.1% TFA/H_2O +0.1% TFA) of the crude reaction mixture. Product identity confirmed by comparison of retention time to an authentic reference standard ((*S*,*S*)-**1**) by UV-HPLC.

Radio/UV-HPLC trace overlay:

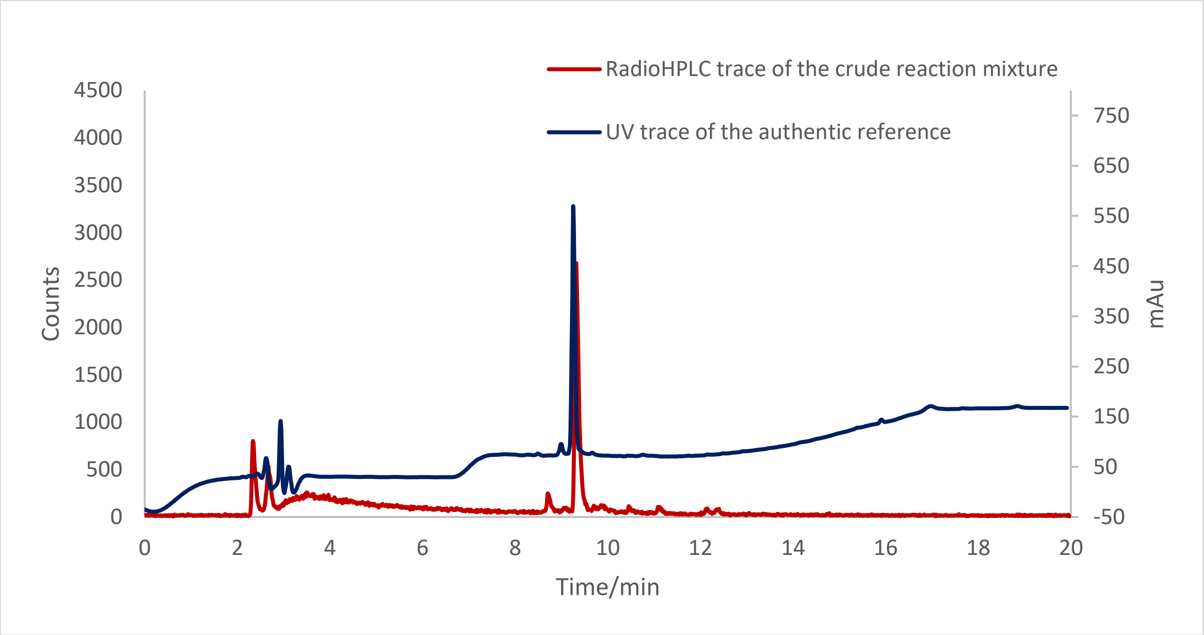

Entry	RCY_{HPLC} of (*S*,*S*)-[^{18}F]**1** (over two steps)
1	25%
2	10%
Average	18% ± 8% (*n* = 2)

Radiosynthesis of (*S*,*S*)-[^{18}F]3

Procedure: To an oven-dried 3 mL v-vial equipped with a magnetic stir bar was added (*S*,*S*)-[^{18}F]**19** (10 µmol), $Cu(OTf)_2py_4$ (2 equiv.) and DMI (300 µL). To the vial was then added an aliquot of the [^{18}F]KF.K_{222} solution (5-20 MBq) in MeCN (approx. 20 µL) (*vide supra*). The reaction vial was purged with air (20 mL) and reaction mixture was stirred at 120 °C for 20 minutes. The reaction mixture was cooled to rt and NaOH (aq., 1 M, 0.1 mL) was added. This mixture was heated at 65 °C for 10 minutes. Once cooled, the reaction mixture was neutralised with HCl (aq., 1 M, 0.1 mL). An aliquot of this mixture was subsequently analysed by radioHPLC for radiochemical yield (RCY) and product identity.

Scheme S3.4. Two-step radiosynthesis of (*S*,*S*)-[^{18}F]**3**

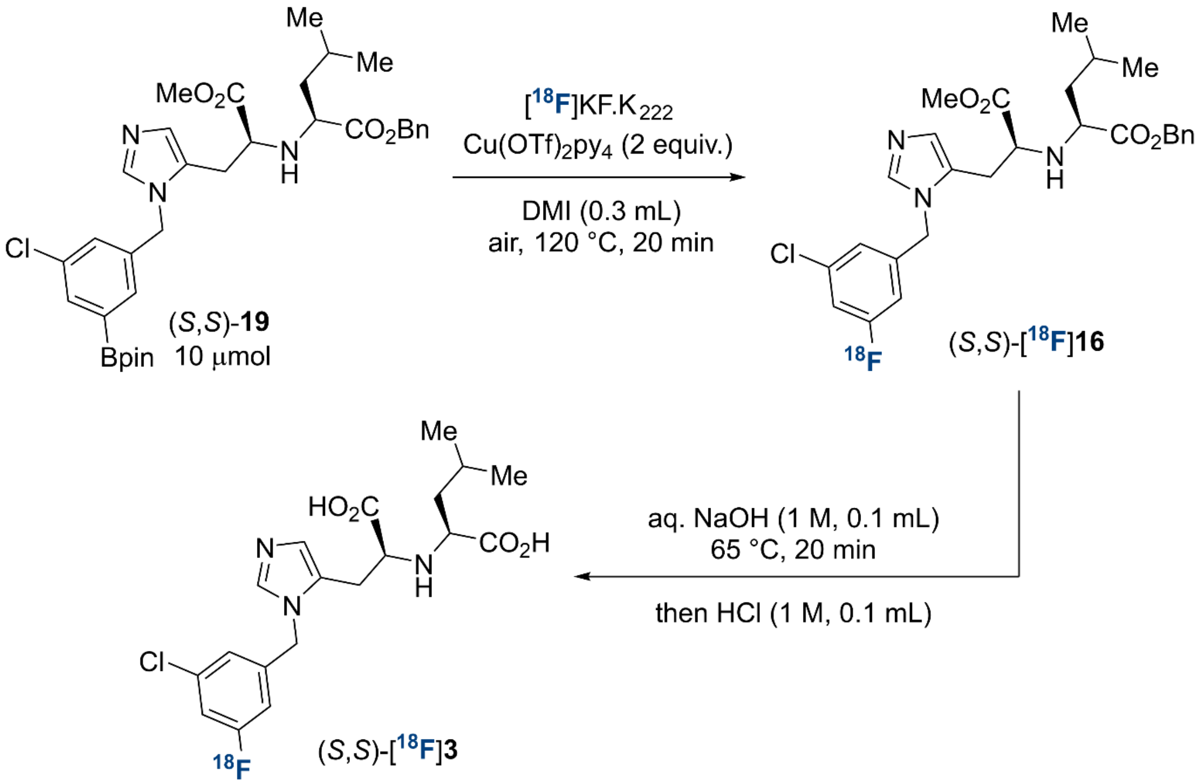

RCY_{HPLC}: radiochemical yield, determined by radio-HPLC analysis (Conditions A, Agilent C18 Eclipse Plus 80 Å 150 x 4.6 mm, gradient: MeCN +0.1% TFA/H_2O +0.1% TFA) of the crude reaction mixture. Product identity confirmed by comparison of retention time to an authentic reference standard ((*S*,*S*)-**3**) by UV-HPLC.

Radio/UV-HPLC trace overlay:

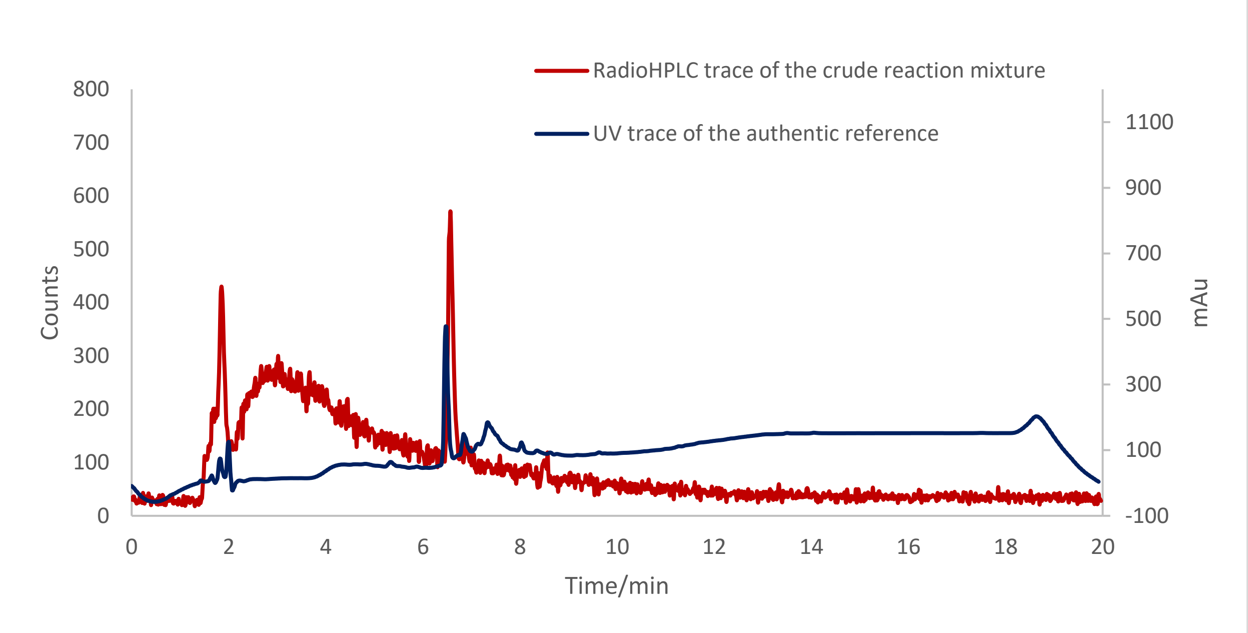

Entry	RCY_{HPLC} of (*S*,*S*)-[^{18}F]**3** (over two steps)
1	10%
2	5%
Average	8% ± 3% (*n* = 2)

4. ACE2 Inhibition Assay

The assay for the inhibition of ACE2 was conducted using a commercially available ACE2 Inhibitor Screening Kit, purchased from Abcam (ACE2 Inhibitor Screening Kit, ab273373; Abcam/BioVision), in 96-well microplates (half area, black, flat bottomed, Greiner Bio-One International) at ambient temperature (set to 23.5˚C), total volume 100 µL. The assay employs a synthetic MCA-based peptide substrate that, upon cleavage by active ACE2, releases a fluorophore measurable by a fluorescence microplate reader (Ex/Em = 320/420 nm). Compounds to be tested were serially diluted with dilution buffer from a stock solution (5 mg/mL) in dimethyl sulfoxide (DMSO) to different concentrations.

Diluted ACE2 enzyme solution was prepared by diluting ACE2 enzyme (20 µL, provided in the kit) with dilution buffer (198 µL, provided in the kit) and was aliquoted and stored at -20 ˚C. Diluted ACE2 enzyme solution (2 µL) was added to assay buffer (48 µL) to make ACE2 enzyme working solution. Inhibitor solution was prepared by adding the control inhibitors (5 µL, 0.5 mM) to dilution buffer (50 µL) and was aliquoted and stored at -20 ˚C. The ACE2 substrate was aliquoted and stored at -20 ˚C. ACE2 substrate solution was prepared by diluting substrate (2 µL) to assay buffer (38 µL).

Five controls were prepared: enzyme control (positive control, inhibition free reaction), inhibitor control (negative control to test inhibition), background control (to remove fluorescence from the background), test compound control (to test whether the test compounds react with the substrate) and vehicle control (to test the effect of solvent DMSO on ACE2 enzyme activity).

For enzyme control, inhibitor control and vehicle control, and test compounds, ACE2 enzyme working solution (50 µL) was added. For background control and test compound control, assay buffer (50 µL) was added. Then, assay buffer (10 µL) was added to enzyme control and background control. DMSO solution in assay buffer (10 µL) was added to vehicle control. Inhibitor solution (10 µL) was added to inhibitor control. Test compound solution (10 µL) was added to the test compound control. For the test compound, compound solution (10 µL) with the different concentrations were added. An example of preparation can be found on Table S3.1. The mixture

and substrate were incubated separately at room temperature for 15 minutes before substrate (40 µL) was added to each test and mixed evenly to start the reaction. Fluorescence was monitored over 61-minutes (excitation = 320 nm, emission = 420 nm) using a Tecan microplate reader (Infinite® 200 PRO NanoQuant Microplate Reader). Fluorescence spectra were measured using the fluorescence intensity mode (excitation = 320 nm).

Table S4.1. Preparation of control and test compounds

Test	Component 1	Component 2	Component 3
Vehicle control	50 µL enzyme[1]	10 µL DMSO (0.1 mM)	40 µL substrate[2]
Background control	50 µL assay buffer	10 µL assay buffer	40 µL substrate
Enzyme control	50 µL enzyme	10 µL assay buffer	40 µL substrate
Inhibitor control	50 µL enzyme	10 µL inhibitor solution	40 µL substrate
Test compound control	50 µL enzyme	10 µL MLN (0.1 mM)	40 µL substrate
LogM = -5	50 µL enzyme	10 µL MLN (0.1 mM)	40 µL substrate
LogM = -6	50 µL enzyme	10 µL MLN (10 µM)	40 µL substrate
LogM = -7	50 µL enzyme	10 µL MLN (1 µM)	40 µL substrate
LogM = -8	50 µL enzyme	10 µL MLN (0.1µM)	40 µL substrate
LogM = -9	50 µL enzyme	10 µL MLN (10 nM)	40 µL substrate
LogM = -10	50 µL enzyme	10 µL MLN (1 nM)	40 µL substrate
LogM = -11	50 µL enzyme	10 µL MLN (0.1 nM)	40 µL substrate
LogM = -12	50 µL enzyme	10 µL MLN (10 pM)	40 µL substrate

[1]Enzyme working solution (2 µL) + assay buffer (48 µL). [2]Substrate solution (2 µL) + assay buffer (38 µL)

5. References

1. Towler, P.; Staker, B.; Prasad, S.G.; Menon, S.; Tang, J.; Parsons, T.; Ryan, D.; Fisher, M.; Williams, D.; Dales, N. A.; Patane, M. A.; Pantoliano, M. W. ACE2 X-Ray Structures Reveal a Large Hinge-bending Motion Important for Inhibitor Binding and Catalysis. *J. Biol. Chem.* **2004**, *279* (17), 17996–18007.
2. Sondergaard, C.R.; Olsson, M. H. M; Rostkowski M.; Jensen, J. H. Improved treatment of ligands and coupling effects in empirical calculation and rationalization of pKa values. *J. Chem. Theory Comput.* **2011**, *7* (7), 2284–2295.
3. Dales, N. A.; Gould, A. E.; Brown, J. A.; Calderwood, E. F.; Guan, B.; Minor, C. A.; Gavin, J. M.; Hales, P.; Kaushik, V. K.; Stewart, M.; Tummino, P. J.; Vickers, C. S.; Ocain, T. D.; Patane, M. A. Substrate-based design of the first class of angiotensin-converting enzyme-related carboxypeptidase (ACE2) inhibitors. *J. Am. Chem. Soc.* **2002**, *124* (40), 11852–11853.
4. Abraham, M. J.; Murtola, T.; Schulz, R.; Páll, S.; Smith, J. C.; Hess, B.; Lindahl, E. GROMACS: High performance molecular simulations through multi-level parallelism from laptops to supercomputers. *SoftwareX* **2015**, *1–2*, 19–25.
5. Ponder, J.W.; Case, D. A. Force Fields for Protein Simulations. *Adv. Protein Chem.* **2003**, *66*, 27–85.
6. Jorgensen, W. L.; Chandrasekhar, J.; Madura, J. D.; Impey, R. W.; Klein, M. L. Comparison of simple potential functions for simulating liquid water. *J. Chem. Phys.* **1983**, *79* (2), 926–935.
7. Wang, J.; Wolf, R. M.; Caldwell, J. W.; Kollman, P. A.; Case, D. A. Development and testing of a general amber force field. *J. Comput. Chem.* **2004**, *25* (9), 1157–1174.
8. Wang, J.; Wang, W.; Kollman, P. A.; Case, D. A. Automatic atom type and bond type perception in molecular mechanical calculations. *J. Mol. Graph. Model.* **2006**, *25* (2), 247–260.
9. Shirts, M. R.; Klein, C.; Swails J. M.; Yin, J.; Gilson, M. K.; Mobley, D. L.; Case, D. A.; Zhong, E. D. Lessons learned from comparing molecular dynamics engines on the SAMPL5 dataset. *J. Comput. Aided Mol. Des.* **2017**, *31*, 147–161.
10. Suruzhon, M.; Senapathi, T.; Bodnarchuk, M. S.; Viner, R.; Wall, I. D.; Barnett, C. B.; Naidoo, K. J.; Essex, J. W. ProtoCaller: Robust Automation of Binding Free Energy Calculations. *J. Chem. Inf. Model.* **2020**, *60* (4), 1917–1921.
11. Hedges, L. O.; Mey A. S. J. S.; Laughton, C. A.; Gervasio, F. L.; Mulholland, A. J.; Woods, C. J.; Michel, J. BioSimSpace: An interoperable Python framework for biomolecular simulation. *J. Open Source Softw.* **2019**, *4* (43), 1831.
12. ProtoCaller: Full automation of relative protein-ligand binding free energy calculations in GROMACS. In: GitHub. https://github.com/protocaller/protocaller. Accessed 20 Jan 2024.
13. Klimovich, P. V.; Shirts, M. R.; Mobley, D. L. Guidelines for the analysis of free energy calculations. *J. Comput. Aided Mol. Des.* **2015**, *29*, 397–411.
14. Bennett, C. H. Efficient estimation of free energy differences from Monte Carlo data. *J. Comput. Phys.* **1976**, *22* (2), 245–268.
15. Xu, J.; Yadan, J. C. Synthesis of *L*-(+)-ergothioneine. *J. Org. Chem.* **1995**, *60* (20), 6296–6301

16. Blankley, C. J.; Hodges, J. C.; Klutchko, S. R.; Himmelsbach, R. J.; Chucholowski, A.; Connolly, C. J.; Neergaard, S. J.; Van Nieuwenhze, M. S.; Sebastian, A. Synthesis and structure-activity relationships of a novel series of non-peptide angiotensin II receptor binding inhibitors specific for the AT2 subtype. *J. Med. Chem.* **1991**, *34* (11), 3248–3260.
17. Iino, T.; Tsukahara, D.; Kamata, K.; Sasaki, K.; Ohyama, S.; Hosaka, H.; Hasegawa, T.; Chiba, M.; Nagata, Y.; Eiki, J.-i. Discovery of potent and orally active 3-alkoxy-5-phenoxy-N-thiazolyl benzamides as novel allosteric glucokinase activators. *Biorg. Med. Chem.* **2009**, *17* (7), 2733–2743.
18. Jia, F.; Li, D. H.; He, S.; Yang, L. P.; Jiang, W. Conformational Effects on the Threading Kinetics of Dumbbell-Shaped Guests into the Cavity of Oxatub[4]arene. *Angew. Chem. Int. Ed.* **2022**, *61* (45), e202212305.

6. NMR Spectra for Novel Compounds

((*S*)-1-Carboxy-2-(1-(4-fluorobenzyl)-1*H*-imidazol-5-yl)ethyl)-*L*-leucine ((*S*,*S*)-1)

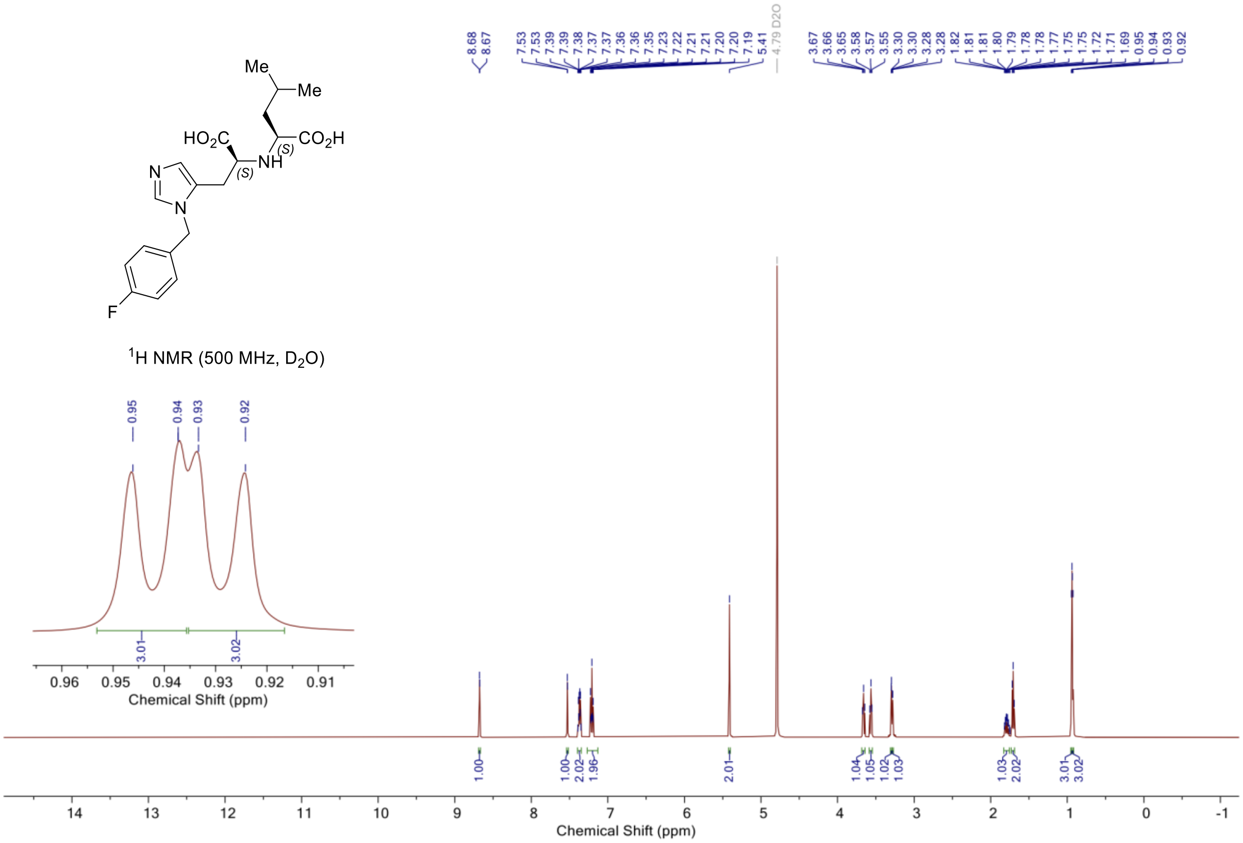

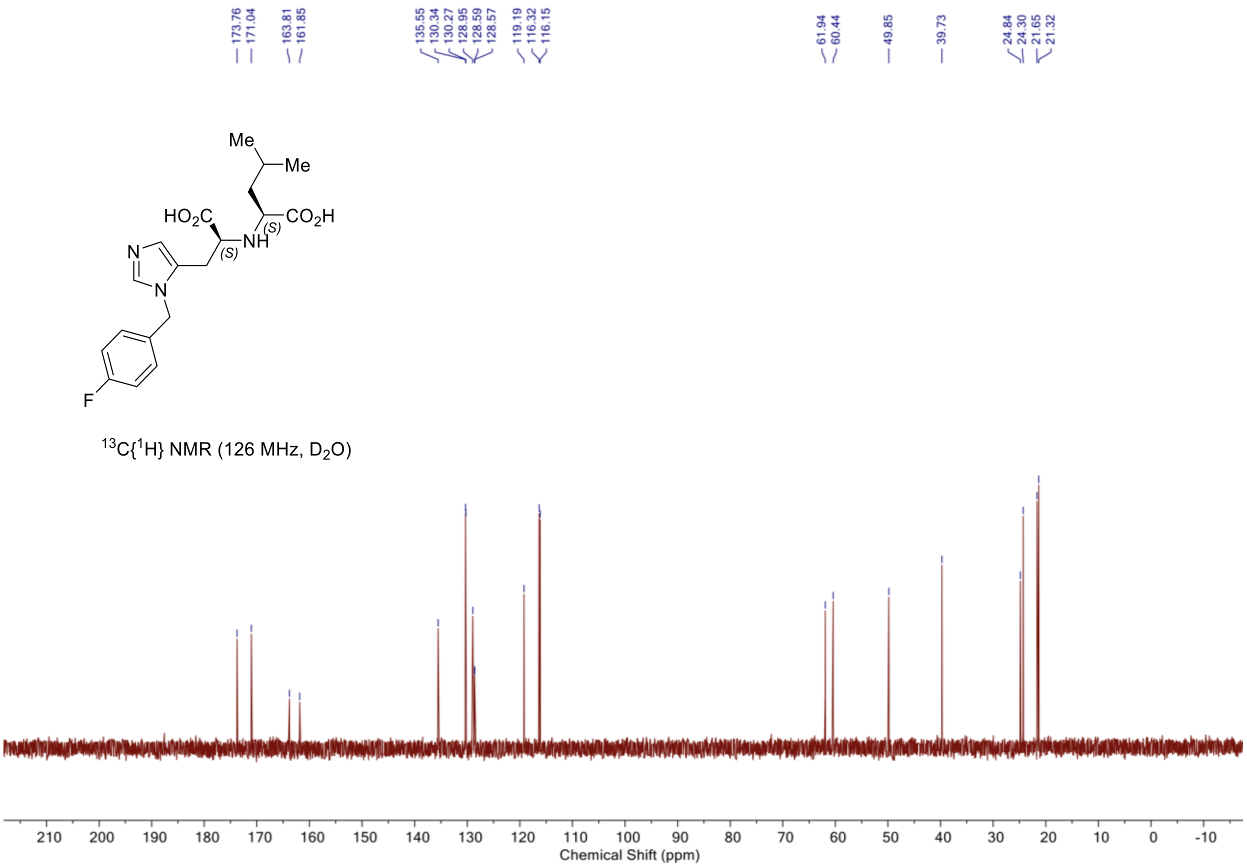
173.76
171.04
163.81
161.85
135.55
130.34
130.27
128.95
128.59
128.57
119.19
116.32
116.15
61.94
60.44
49.85
39.73
24.84
24.30
21.65
21.32
Me
Me
HO2C
CO2H
(S)
(S)
NH
N
N
F
13C{1H} NMR (126 MHz, D2O)
Chemical Shift (ppm)

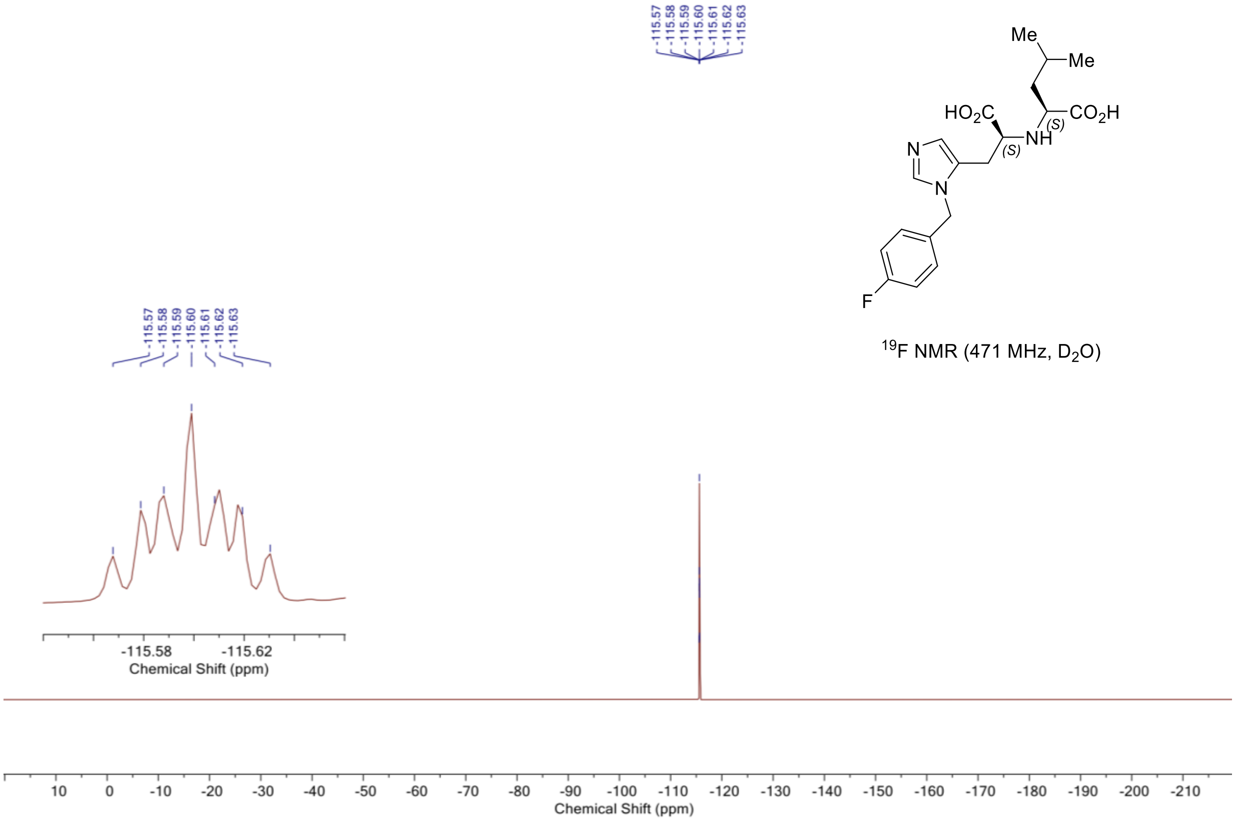
-115.57
-115.58
-115.59
-115.60
-115.61
-115.62
-115.63
Me
Me
HO2C
CO2H
(S)
(S)
NH
N
N
F
19F NMR (471 MHz, D2O)
-115.58
-115.62
Chemical Shift (ppm)

((*S*)-1-Carboxy-2-(1-(4-fluorobenzyl)-1*H*-imidazol-5-yl)ethyl)-*D*-leucine ((*S*,*R*)-1)

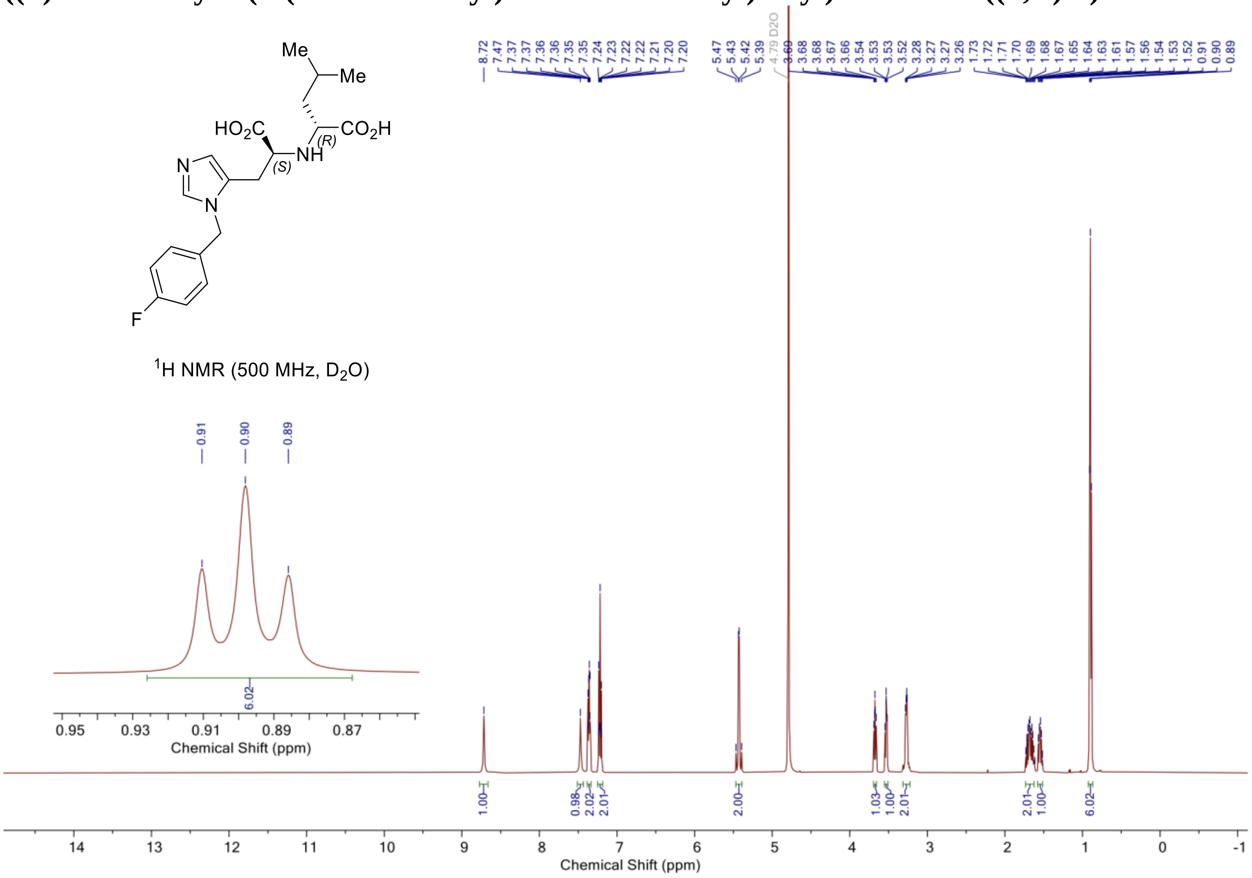

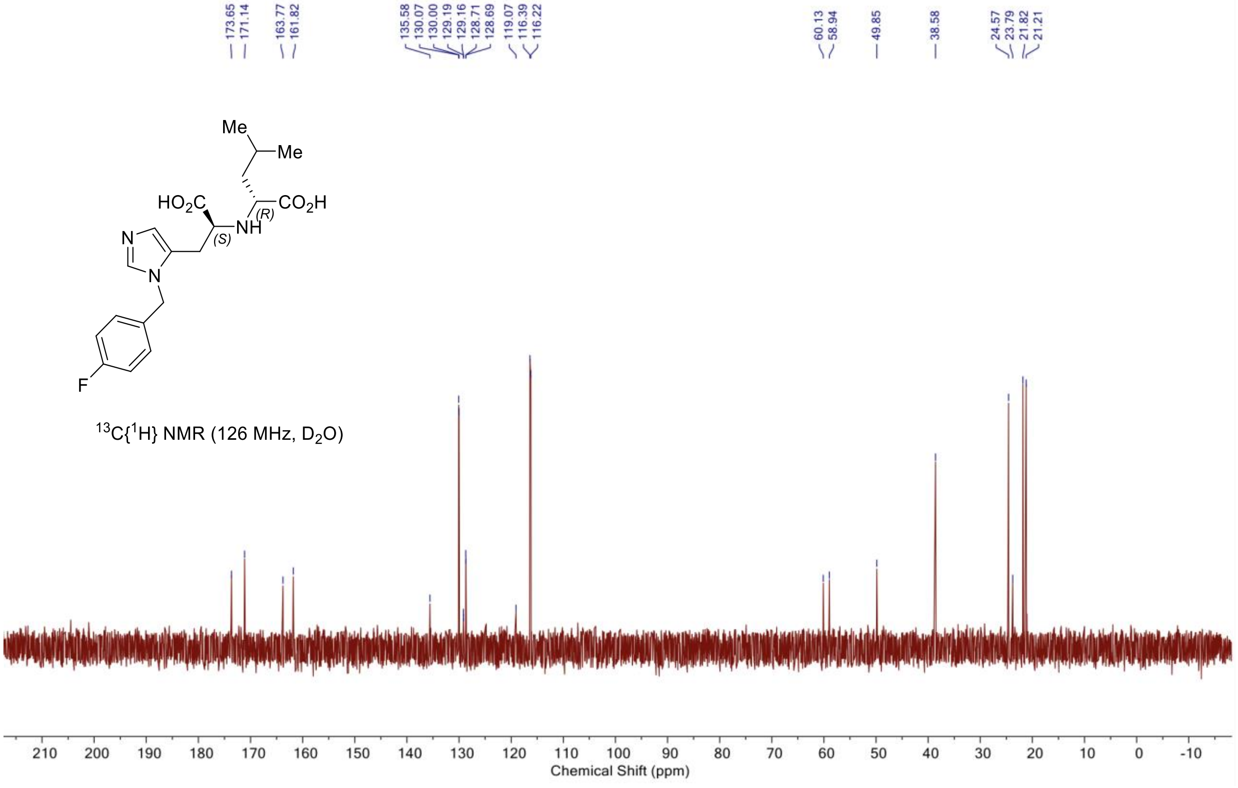

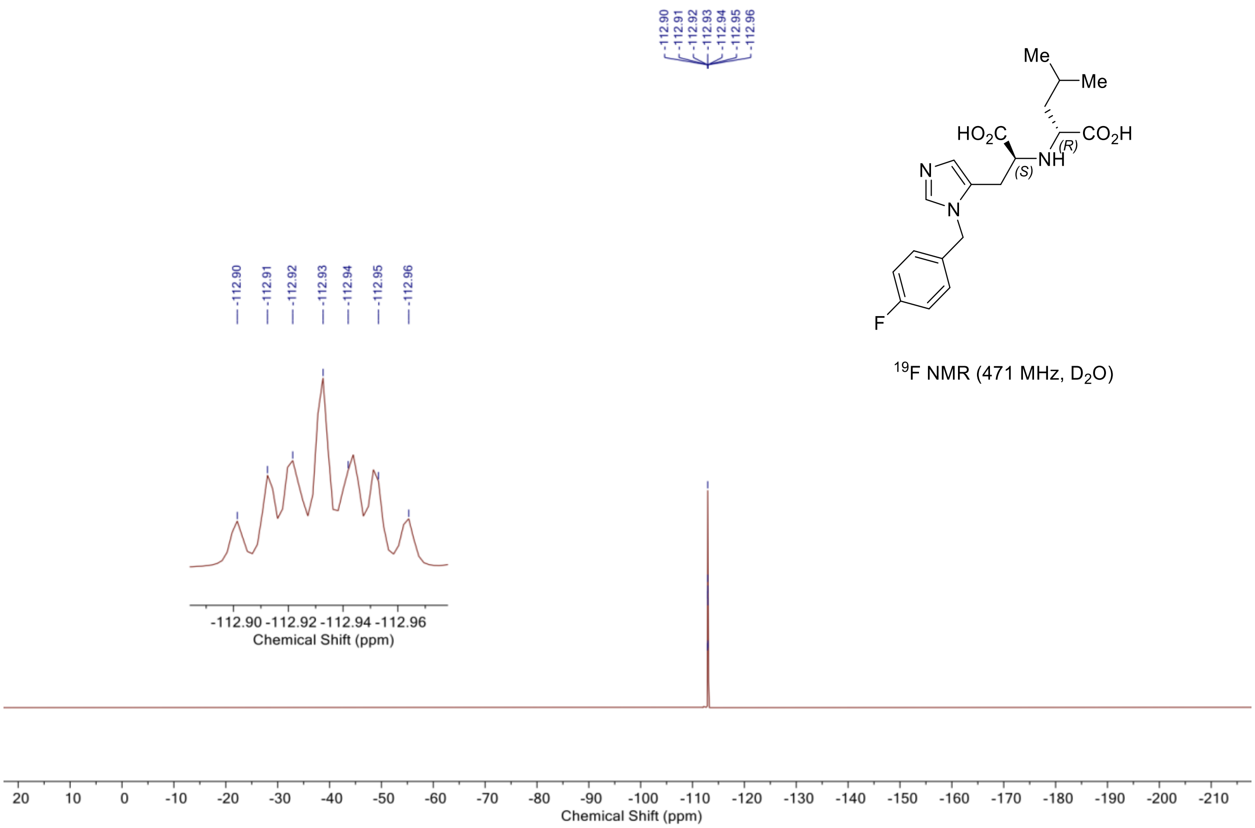

-112.90
-112.91
-112.92
-112.93
-112.94
-112.95
-112.96
Me
Me
HO2C
CO2H
(R)
NH
(S)
N
N
F
19F NMR (471 MHz, D2O)
-112.90 -112.92 -112.94 -112.96
Chemical Shift (ppm)
20 10 0 -10 -20 -30 -40 -50 -60 -70 -80 -90 -100 -110 -120 -130 -140 -150 -160 -170 -180 -190 -200 -210
Chemical Shift (ppm)

((*S*)-1-Carboxy-2-(1-(3-fluoro-5-methylbenzyl)-1*H*-imidazol-5-yl)ethyl)-*L*-leucine ((*S*,*S*)-2)

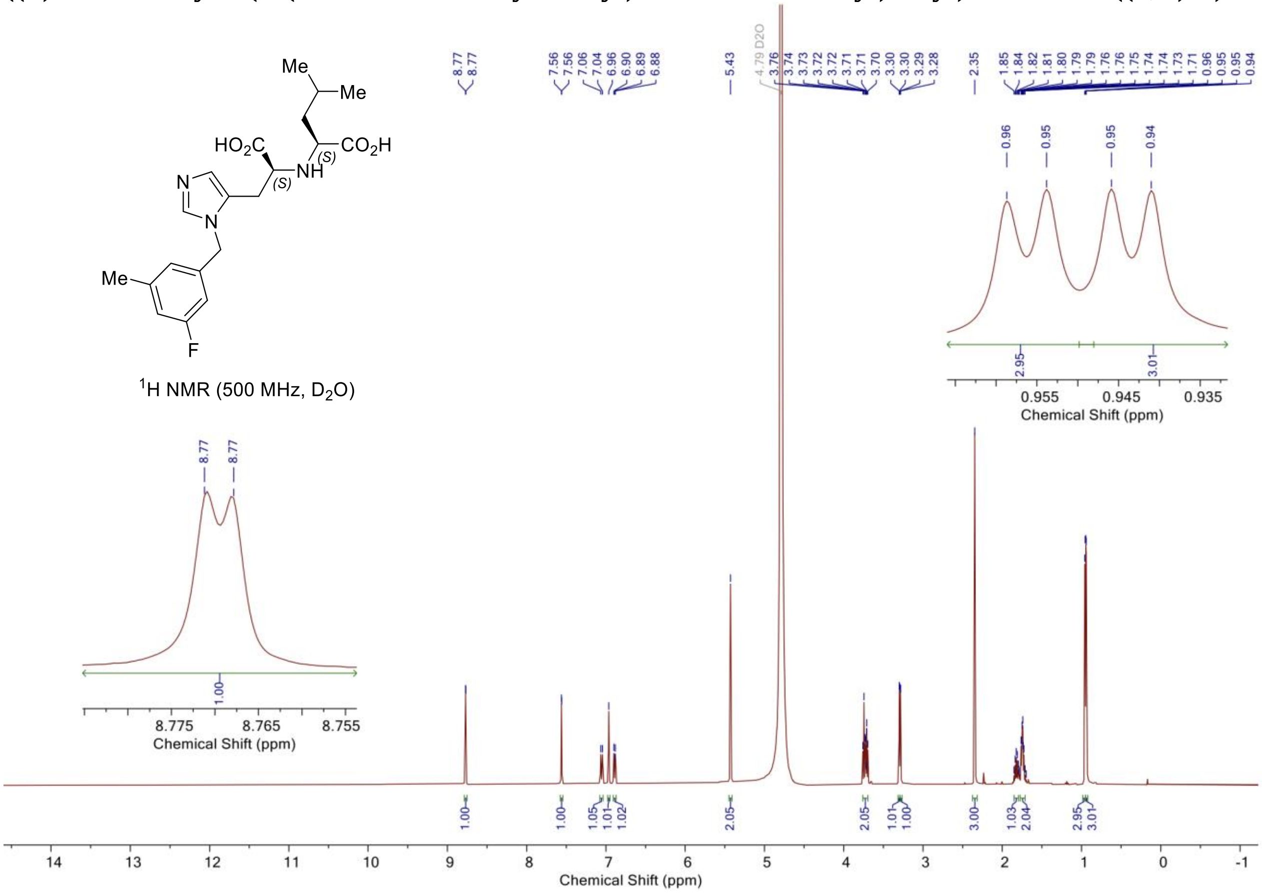

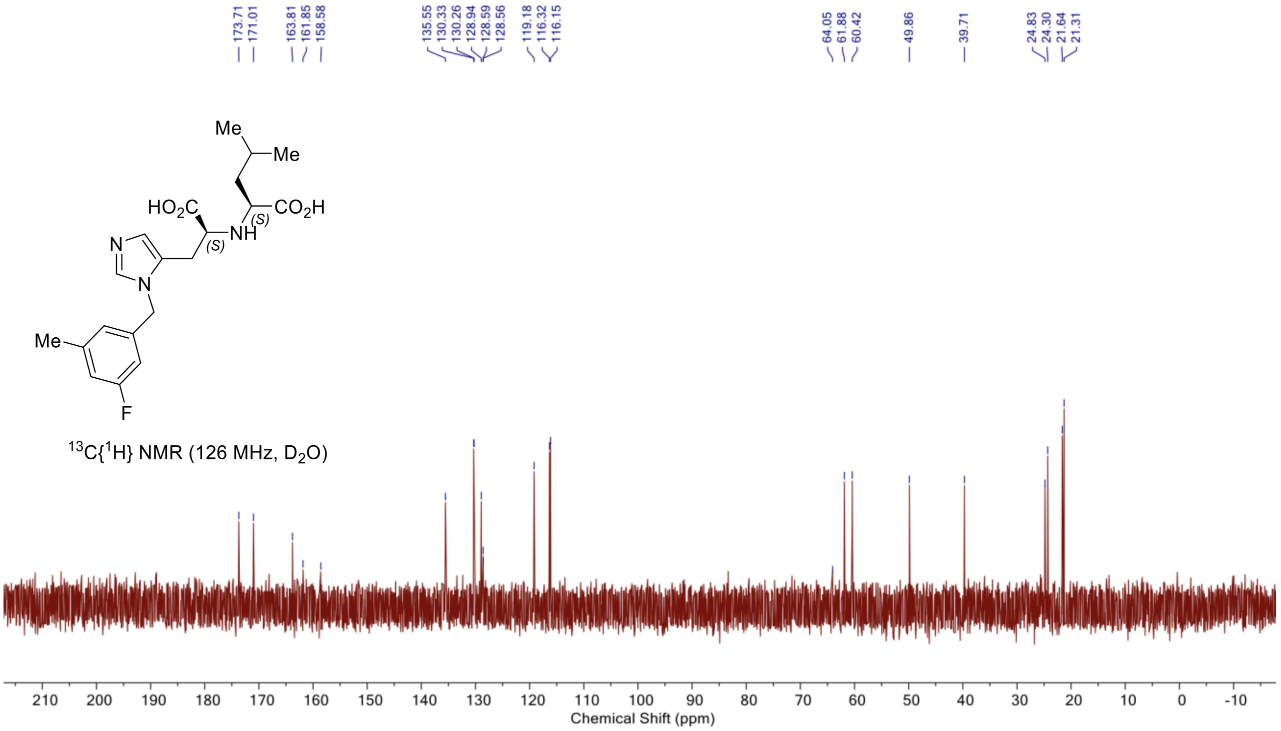
173.71
171.01
163.81
161.85
158.58
135.55
130.33
130.26
128.94
128.59
128.56
119.18
116.32
116.15
64.05
61.88
60.42
49.86
39.71
24.83
24.30
21.64
21.31
Me
Me
HO_2C
CO_2H
(S)
NH
(S)
N
N
Me
F
$^{13}C\{^1H\}$ NMR (126 MHz, D_2O)
210 200 190 180 170 160 150 140 130 120 110 100 90 80 70 60 50 40 30 20 10 0 -10
Chemical Shift (ppm)

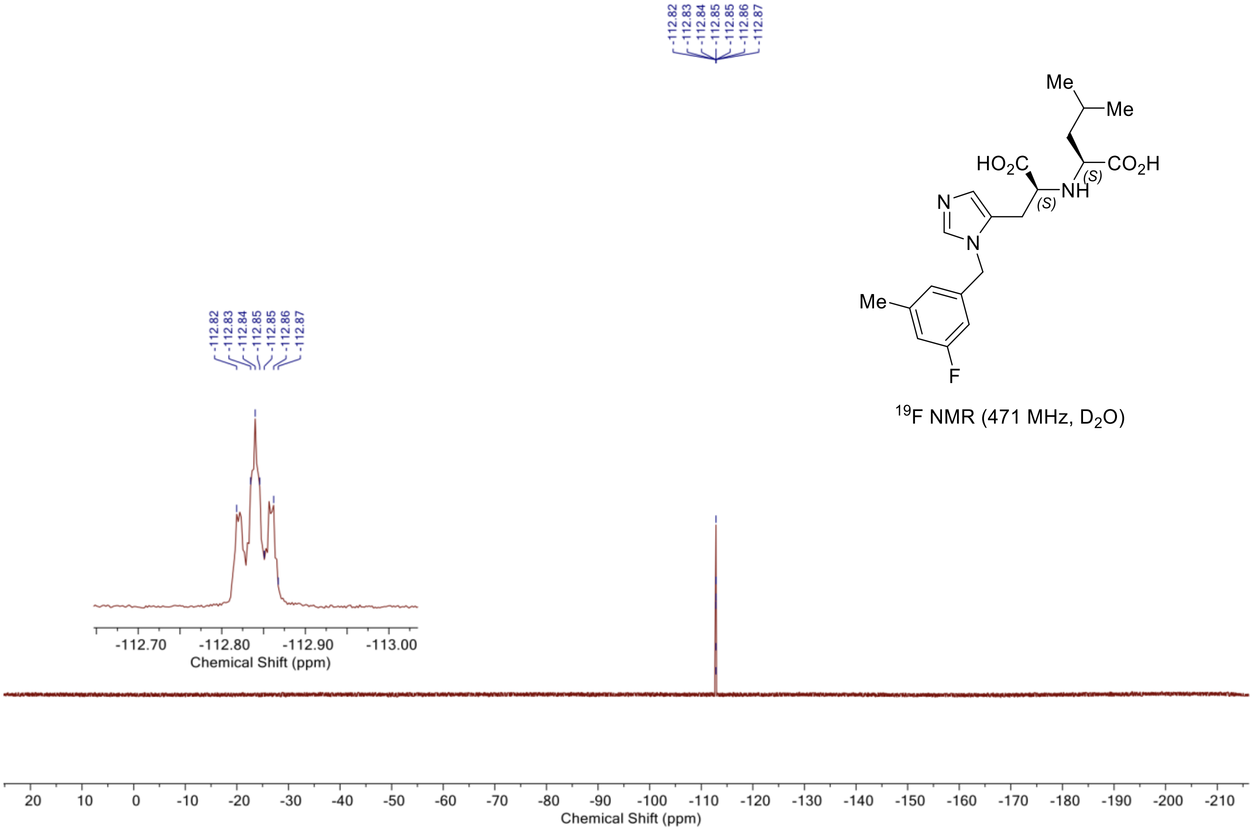
-112.82
-112.83
-112.84
-112.85
-112.85
-112.86
-112.87
Me
Me
HO_2C
CO_2H
(S)
NH
(S)
N
N
Me
F
^{19}F NMR (471 MHz, D_2O)
-112.70 -112.80 -112.90 -113.00
Chemical Shift (ppm)
20 10 0 -10 -20 -30 -40 -50 -60 -70 -80 -90 -100 -110 -120 -130 -140 -150 -160 -170 -180 -190 -200 -210
Chemical Shift (ppm)

((*S*)-1-Carboxy-2-(1-(3-chloro-5-fluorobenzyl)-1*H*-imidazol-5-yl)ethyl)-*L*-leucine ((*S*,*S*)-3)

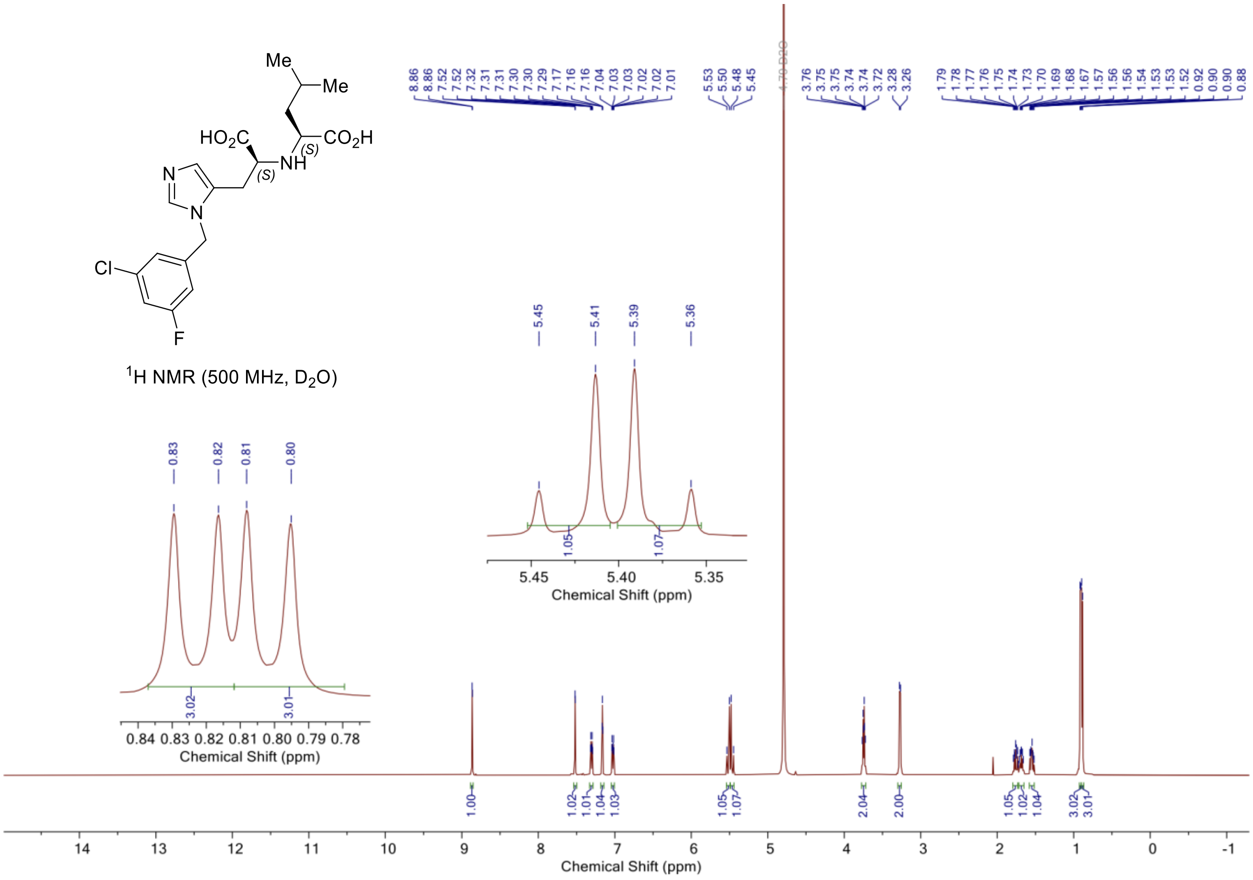

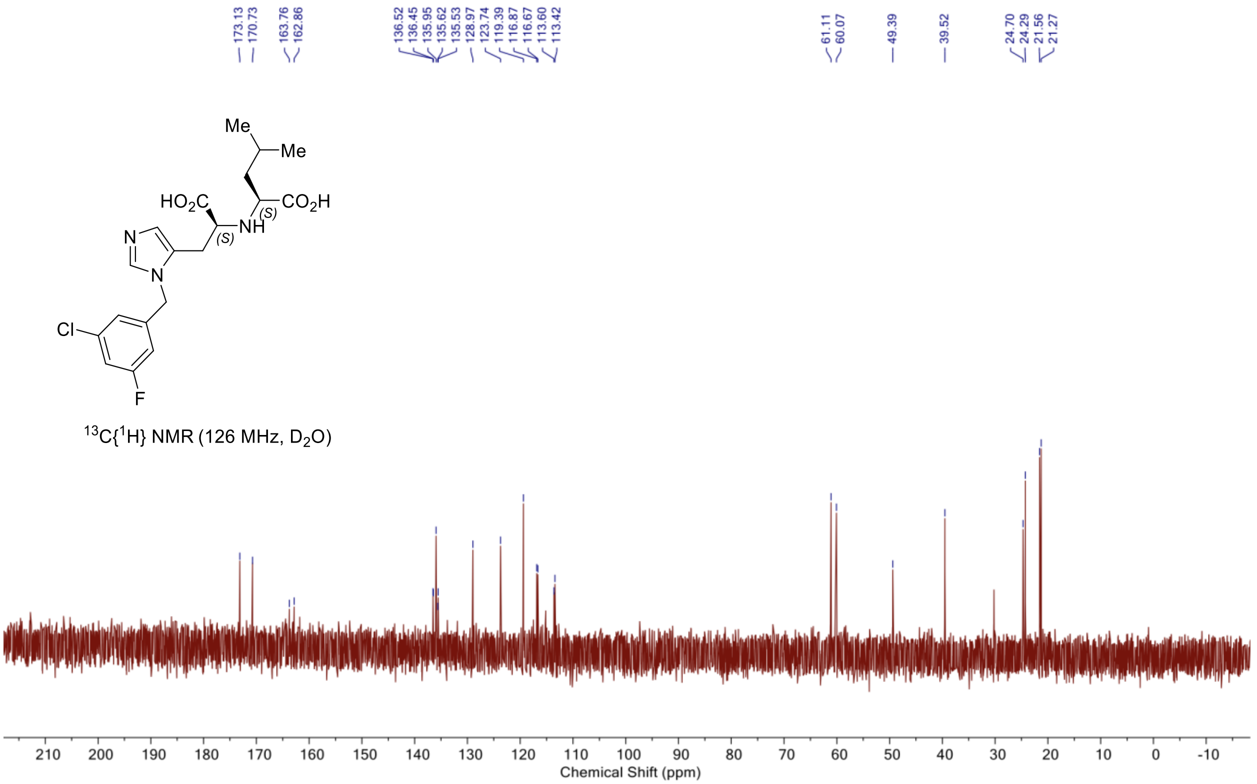
173.13
170.73
163.76
162.86
136.52
136.45
135.95
135.62
135.53
128.97
123.74
119.39
116.87
116.67
113.60
113.42
61.11
60.07
49.39
39.52
24.70
24.29
21.56
21.27
Me
Me
HO_2C
CO_2H
(S)
NH
(S)
N
N
Cl
F
$^{13}C\{^1H\}$ NMR (126 MHz, D_2O)
Chemical Shift (ppm)

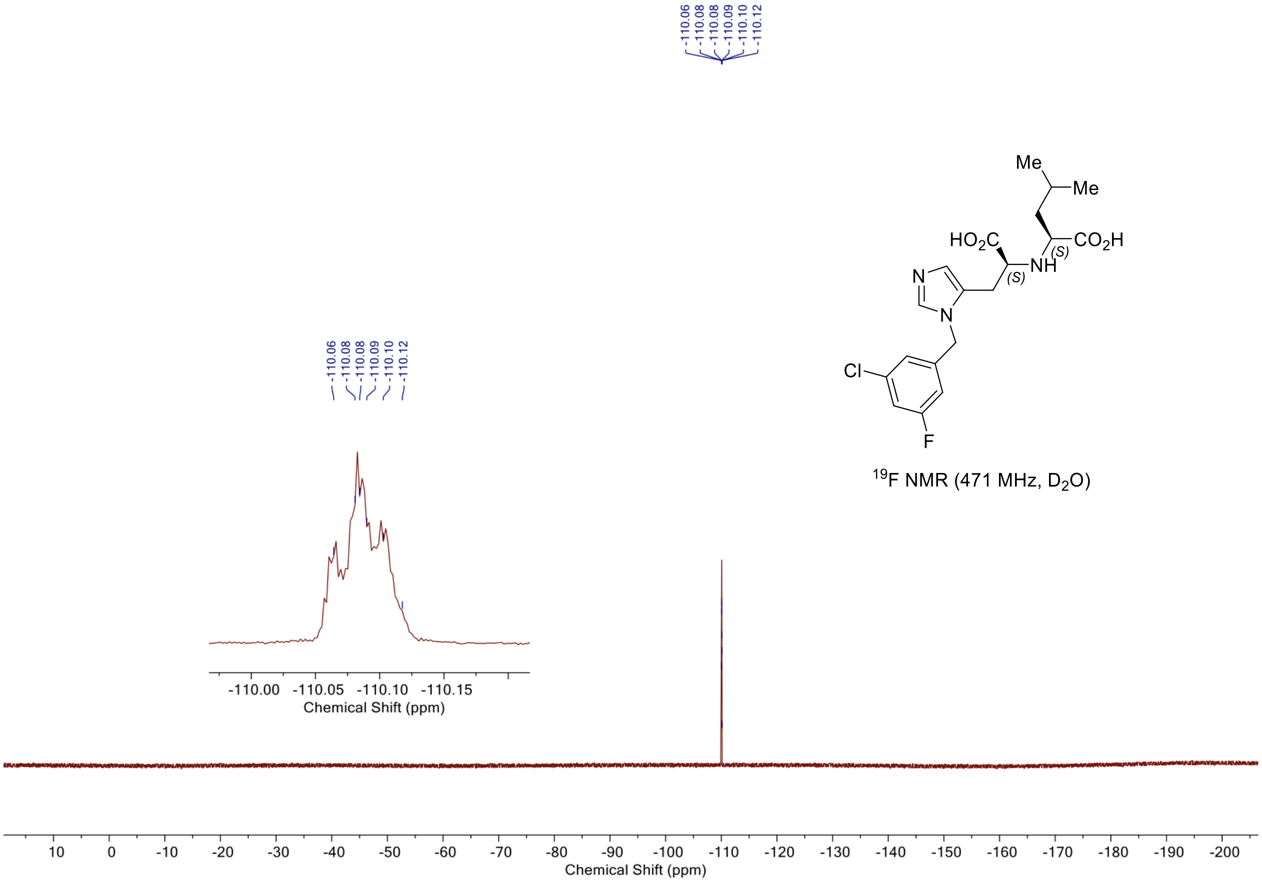
-110.06
-110.08
-110.08
-110.09
-110.10
-110.12
Me
Me
HO_2C
CO_2H
(S)
NH
(S)
N
N
Cl
F
^{19}F NMR (471 MHz, D_2O)
Chemical Shift (ppm)

((*S*)-1-Carboxy-2-(1-(3-(2-fluoroethoxy)-5-methoxybenzyl)-1*H*-imidazol-5-yl)ethyl)-*L*-leucine ((*S,S*)-4)

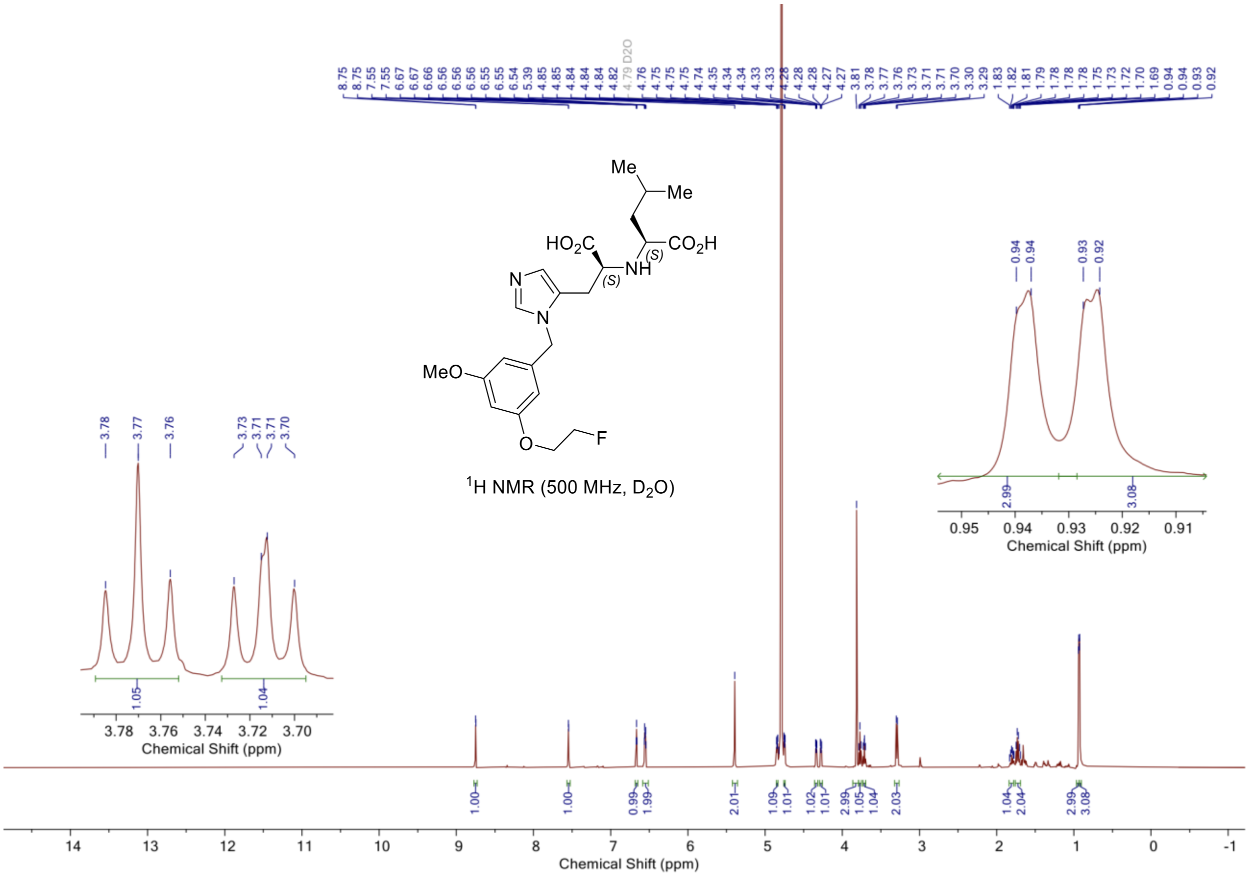

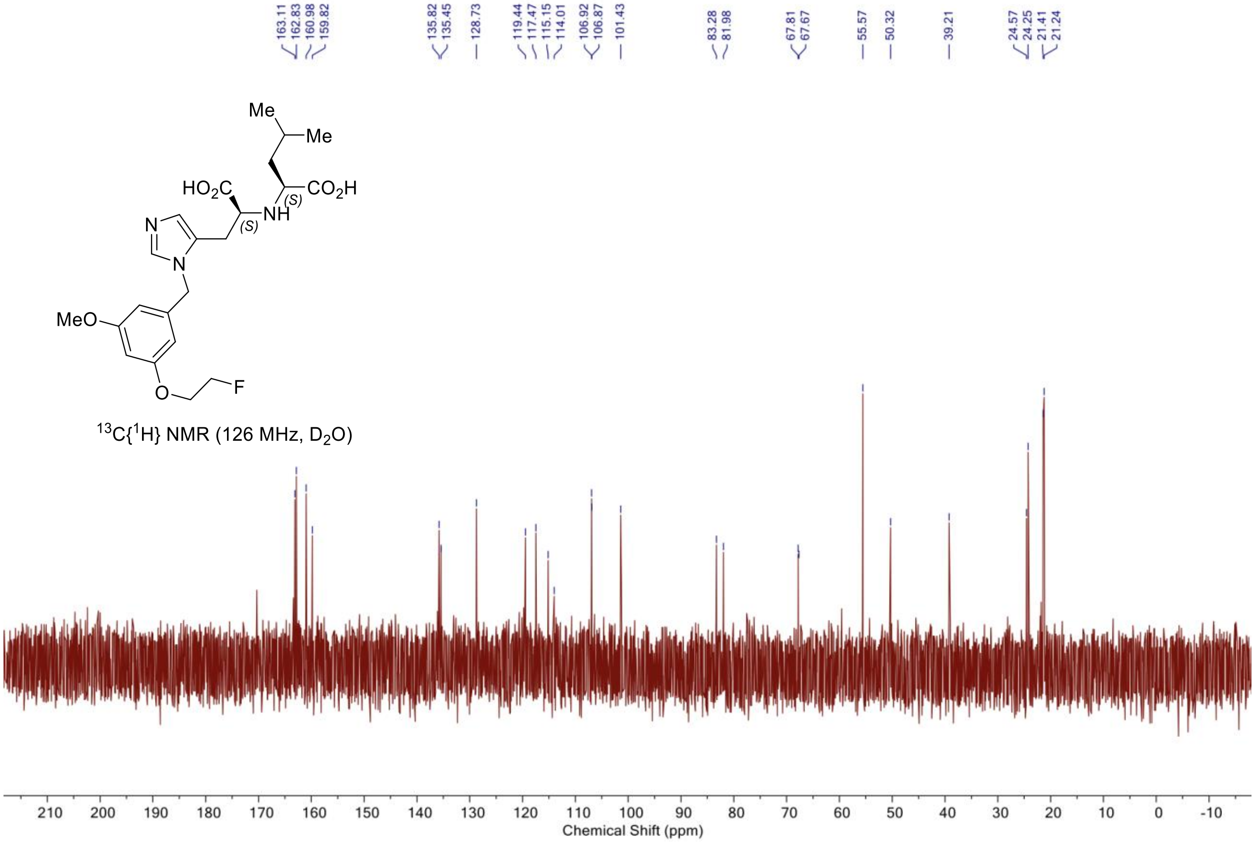
163.11
162.83
160.98
159.82
135.82
135.45
128.73
119.44
117.47
115.15
114.01
106.92
106.87
101.43
83.28
81.98
67.81
67.67
55.57
50.32
39.21
24.57
24.25
21.41
21.24
Me
Me
HO2C
CO2H
(S)
(S)
NH
N
N
MeO
O
F
13C{1H} NMR (126 MHz, D2O)
Chemical Shift (ppm)

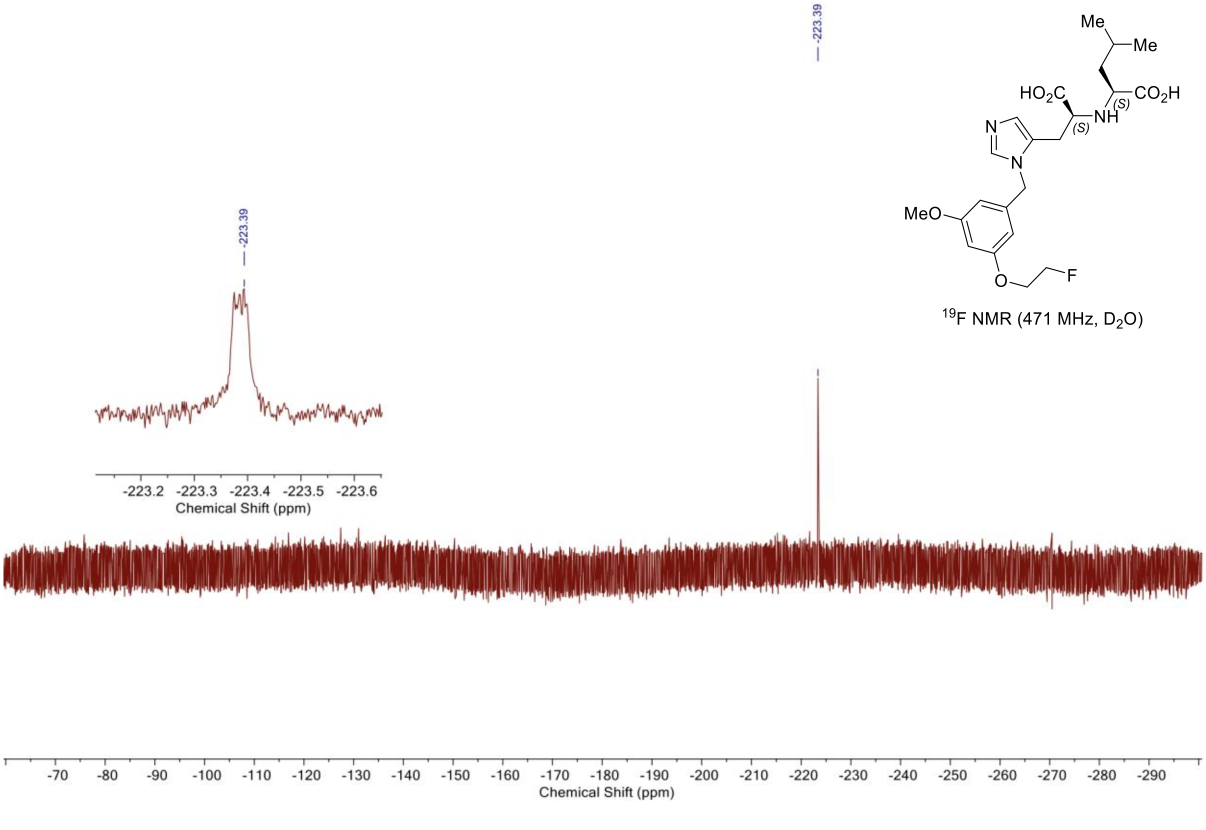
-223.39
-223.39
Chemical Shift (ppm)
Me
Me
HO2C
CO2H
(S)
(S)
NH
N
N
MeO
O
F
19F NMR (471 MHz, D2O)
Chemical Shift (ppm)

Methyl *Nα*-(*tert*-butoxycarbonyl)-*Nπ*-(4-fluorobenzyl)-*L*-histidinate ((*S*)-S2)

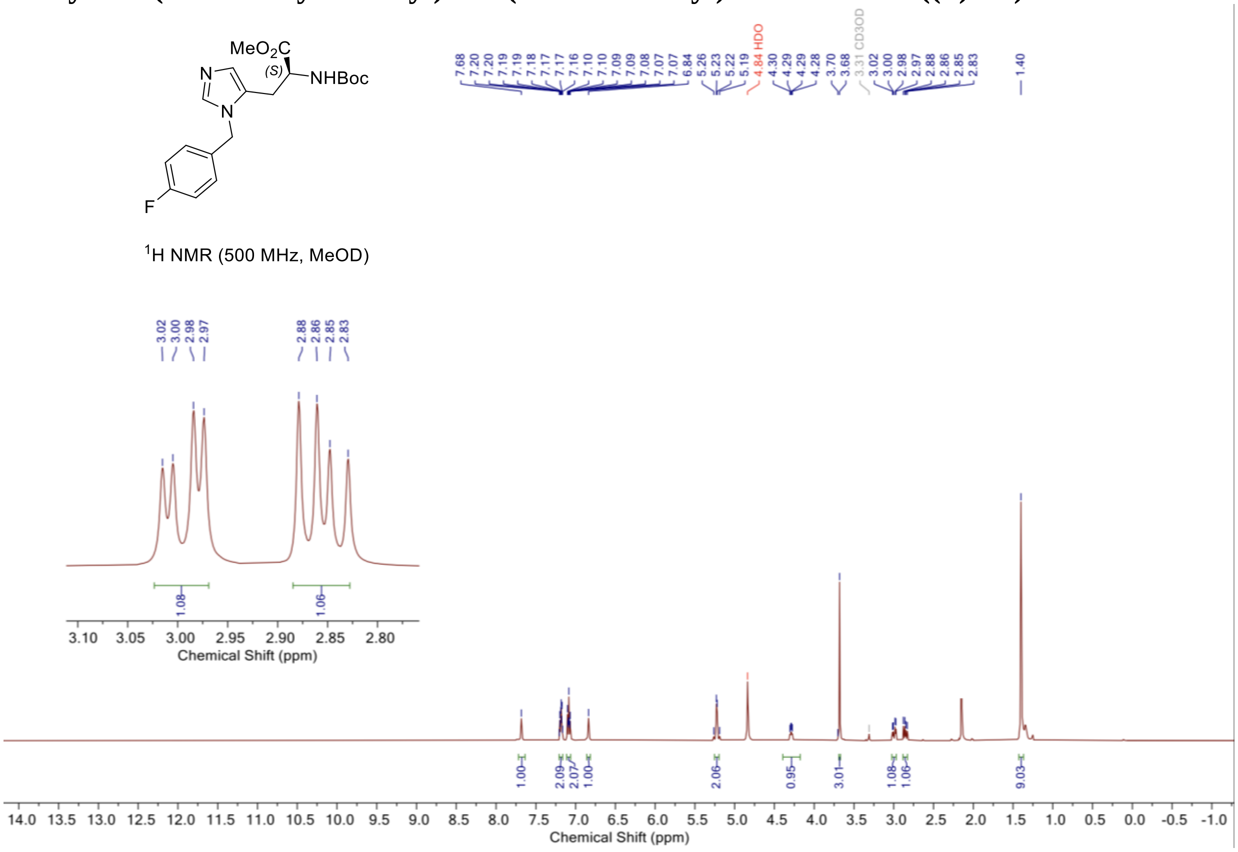

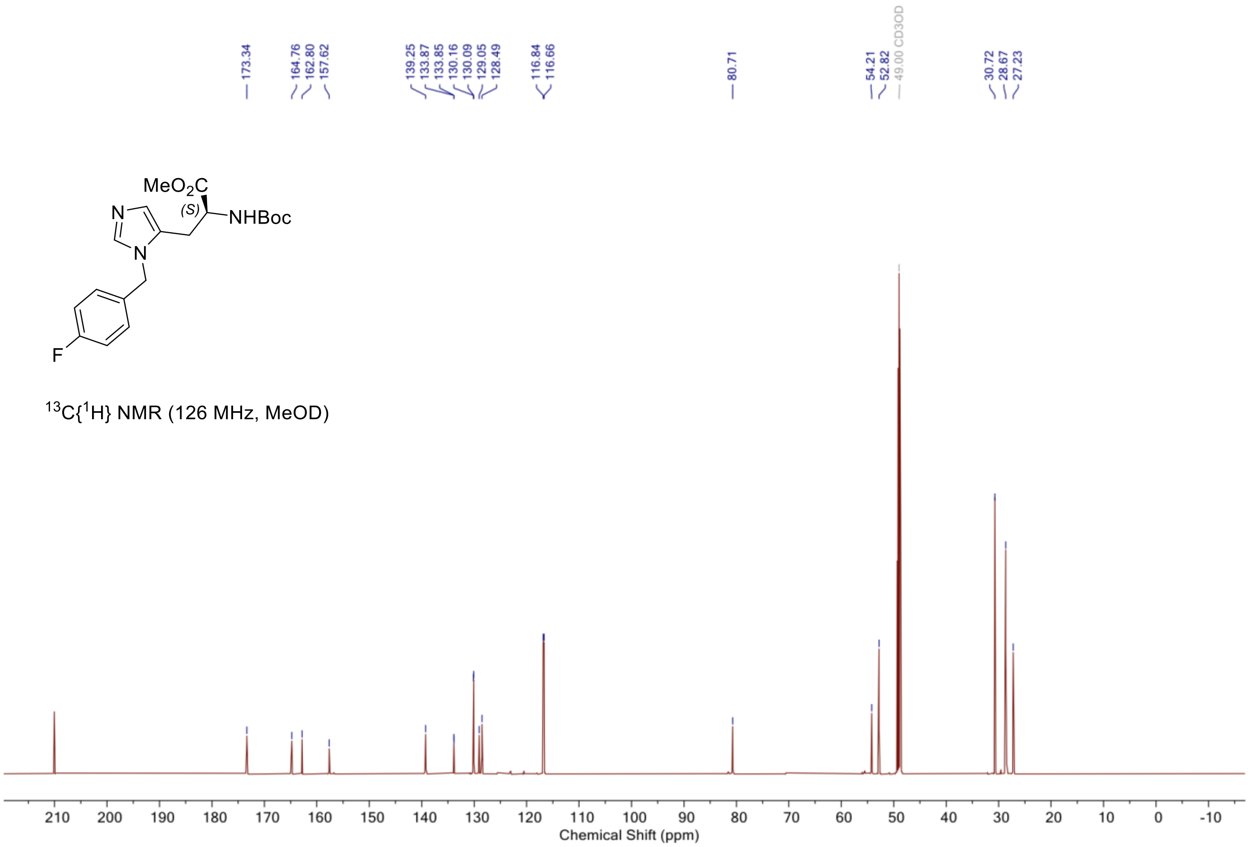
173.34
164.76
162.80
157.62
139.25
133.87
133.85
130.16
130.09
129.05
128.49
116.84
116.66
80.71
54.21
52.82
49.00 CD3OD
30.72
28.67
27.23
MeO2C
(S)
NHBoc
N
N
F
13C{1H} NMR (126 MHz, MeOD)
Chemical Shift (ppm)

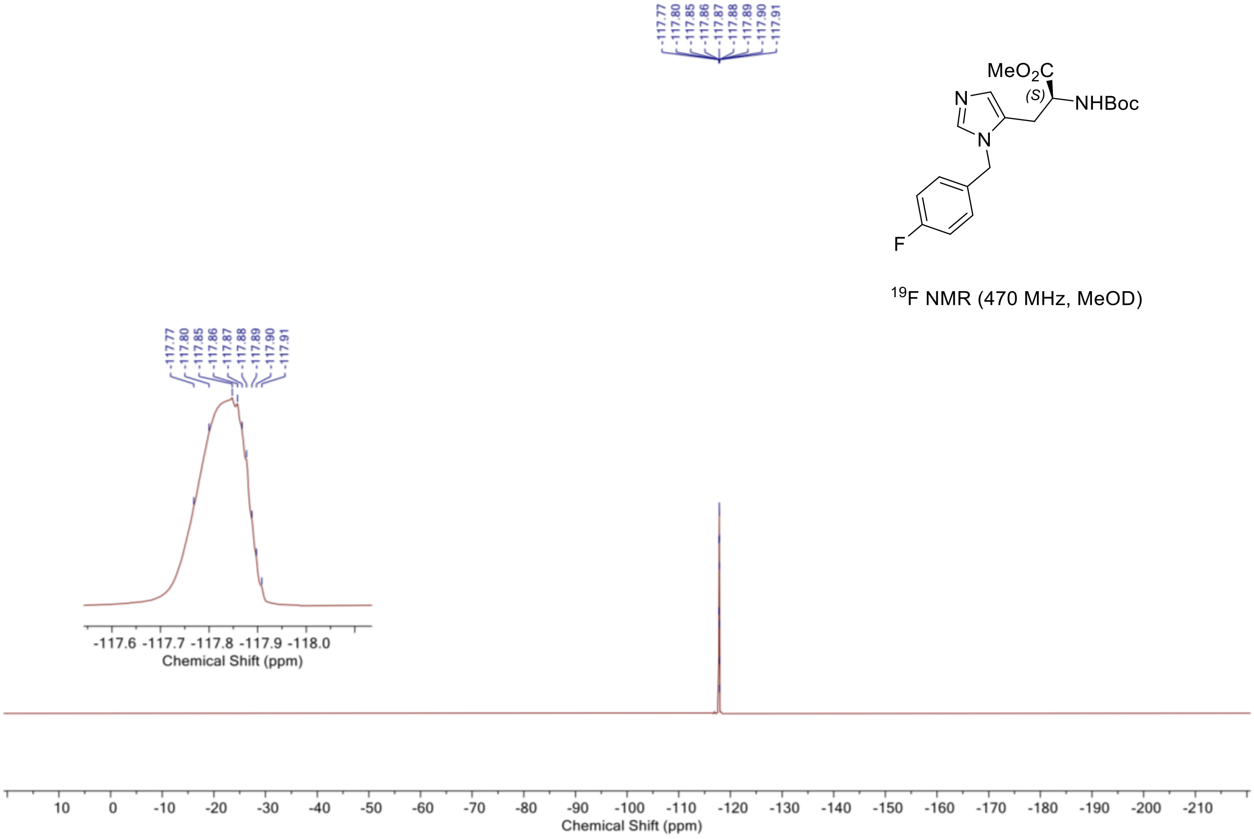
-117.77
-117.80
-117.85
-117.86
-117.87
-117.88
-117.89
-117.90
-117.91
MeO2C
(S)
NHBoc
N
N
F
19F NMR (470 MHz, MeOD)
-117.6 -117.7 -117.8 -117.9 -118.0
Chemical Shift (ppm)
Chemical Shift (ppm)

Methyl *Nπ*-(4-fluorobenzyl)-*Nα*-((2-nitrophenyl)sulfonyl)-*L*-histidinate ((*S*)-S6)

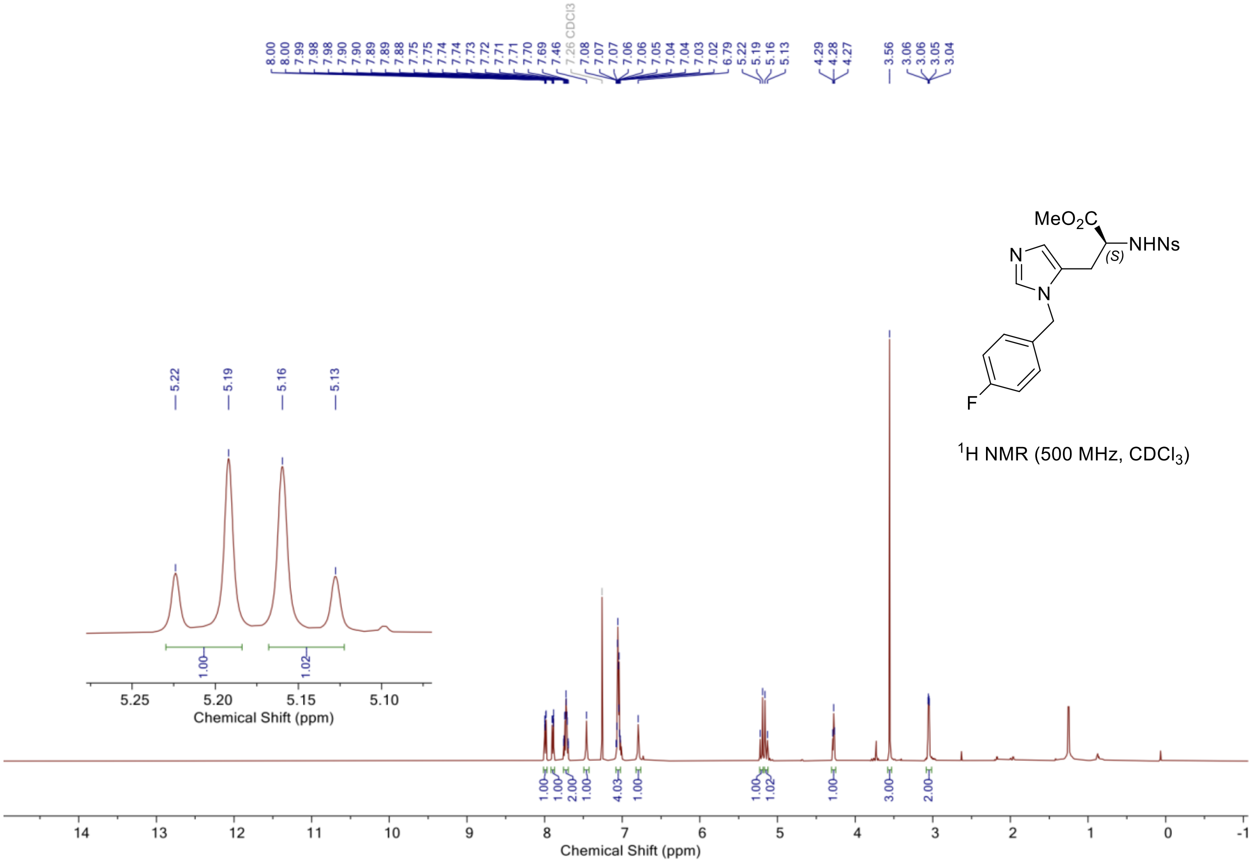

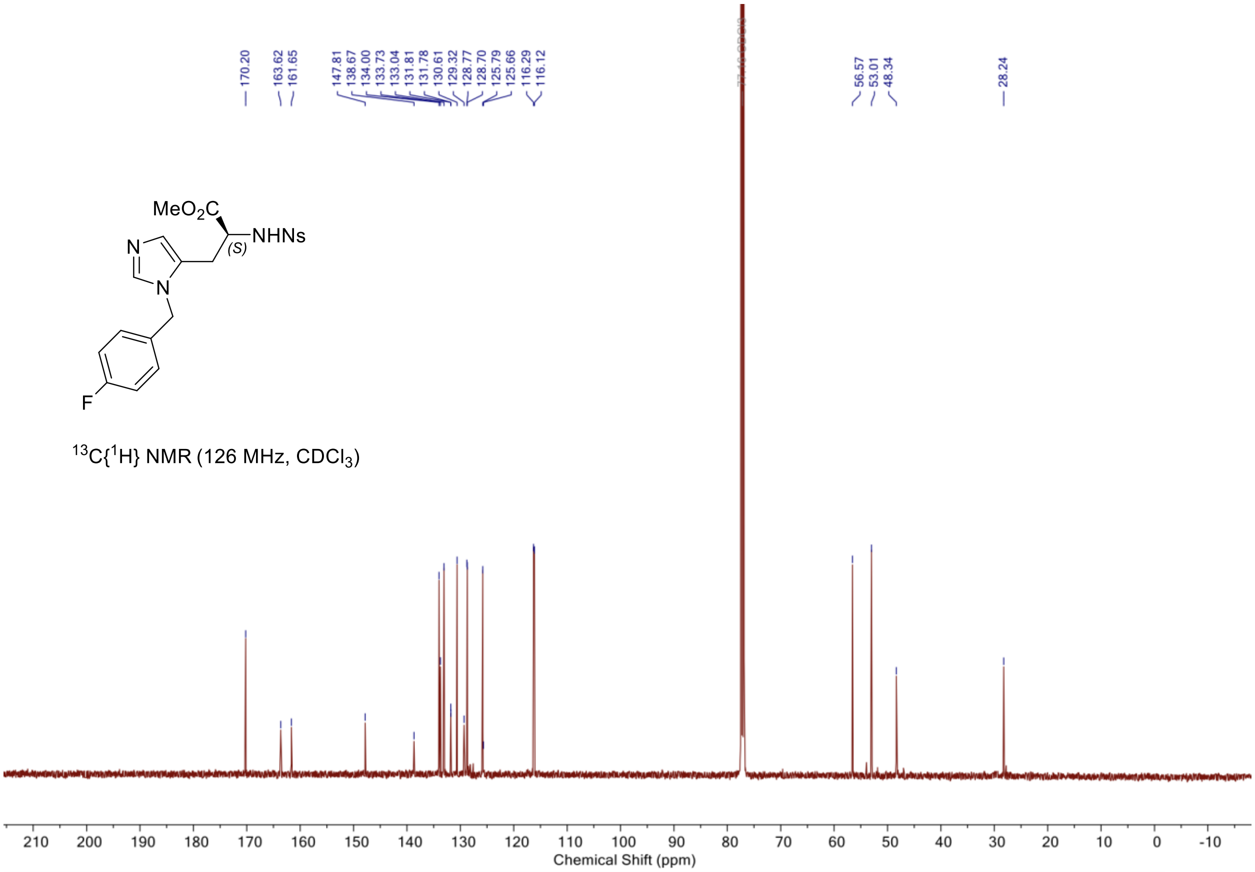
170.20
163.62
161.65
147.81
138.67
134.00
133.73
133.04
131.81
131.78
130.61
129.32
128.77
128.70
125.79
125.66
116.29
116.12
56.57
53.01
48.34
28.24
MeO2C
NHNs
(S)
N
N
F
13C{1H} NMR (126 MHz, CDCl3)
Chemical Shift (ppm)

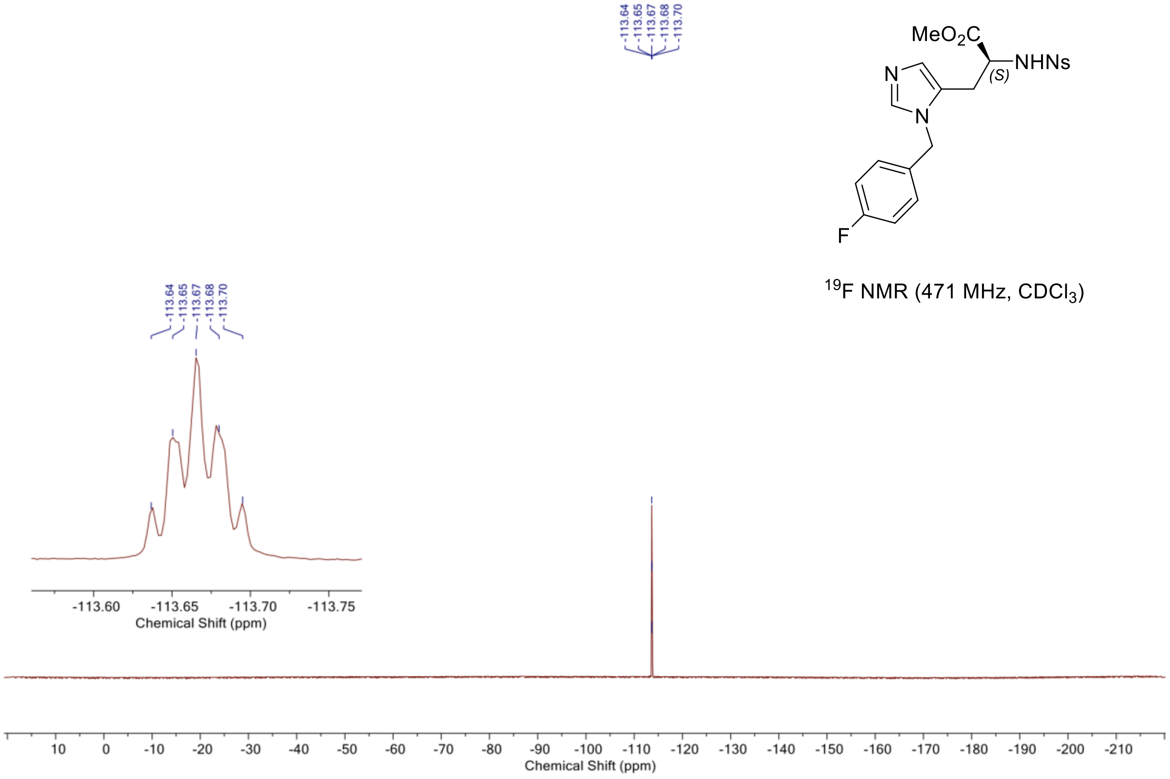
-113.64
-113.65
-113.67
-113.68
-113.70
MeO2C
NHNs
(S)
N
N
F
19F NMR (471 MHz, CDCl3)
Chemical Shift (ppm)

Methyl *Nπ*-(4-fluorobenzyl)-*Nα*-((2-nitrophenyl)sulfonyl)-*L*-histidinate ((*S*)-6)

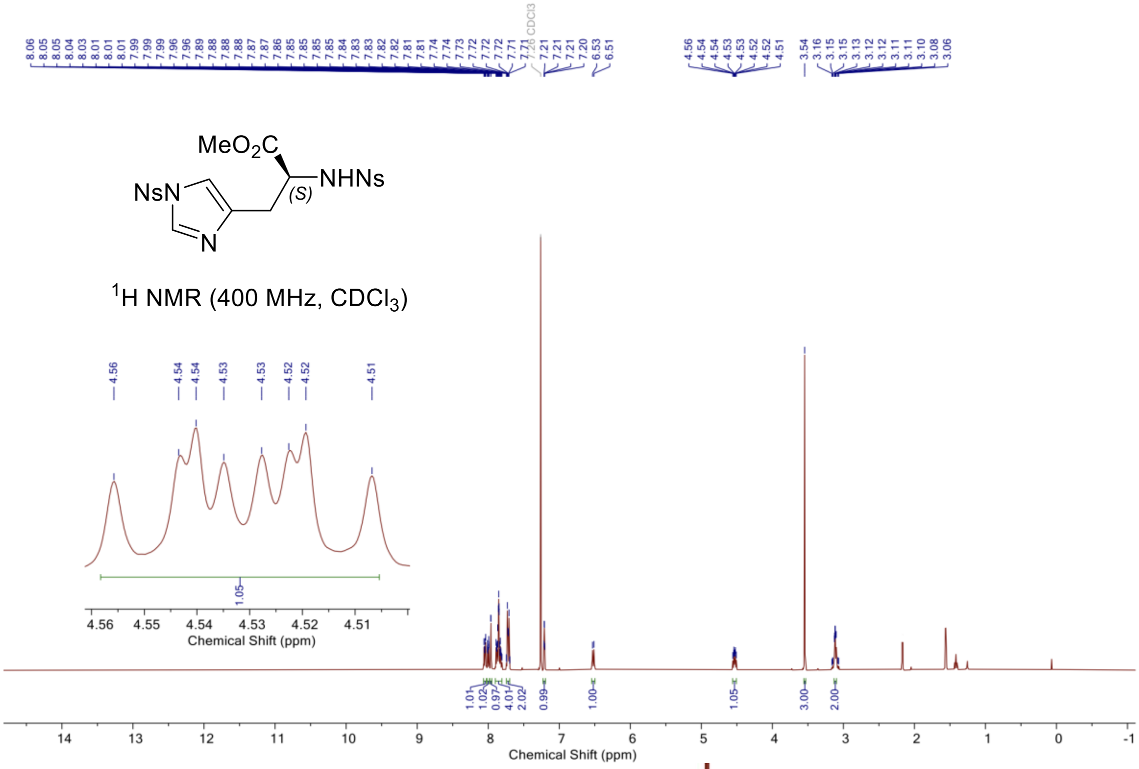

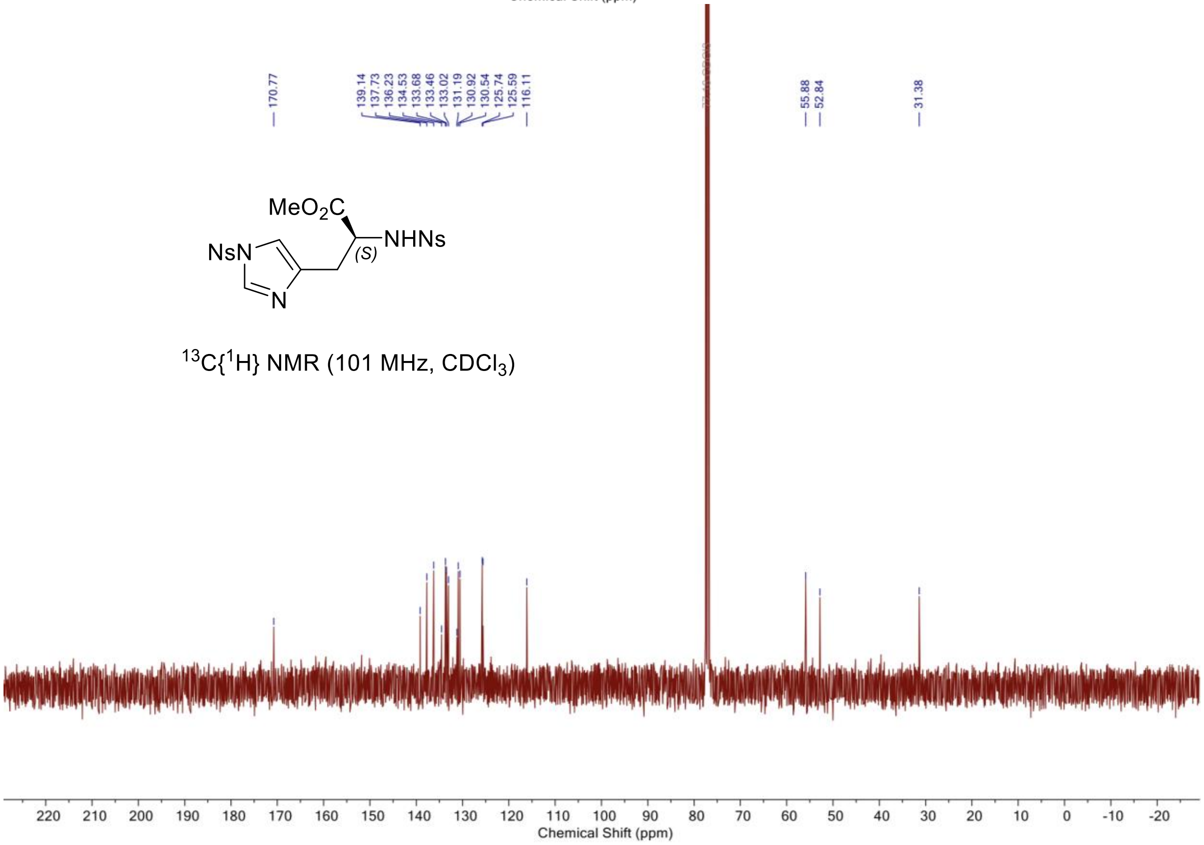

Benzyl (*R*)-2-hydroxy-4-methylpentanoate ((*R*)-7)

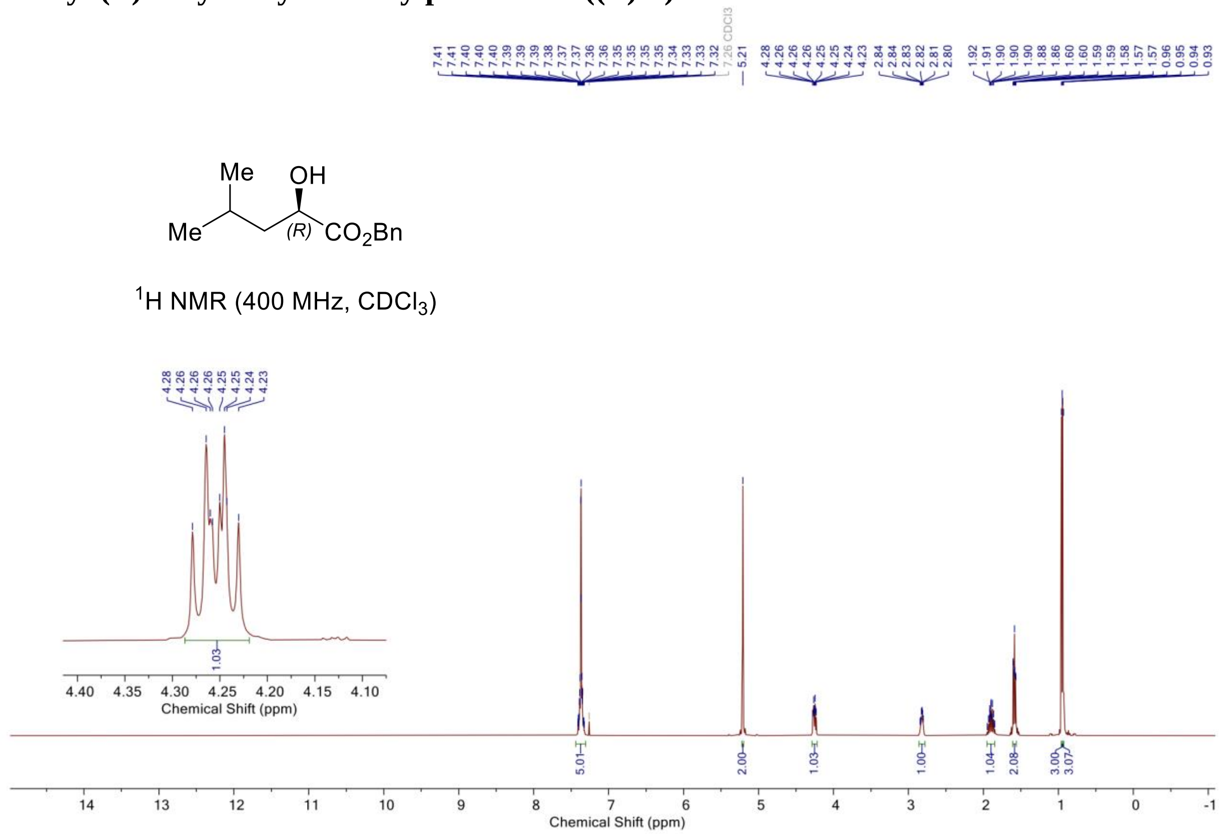

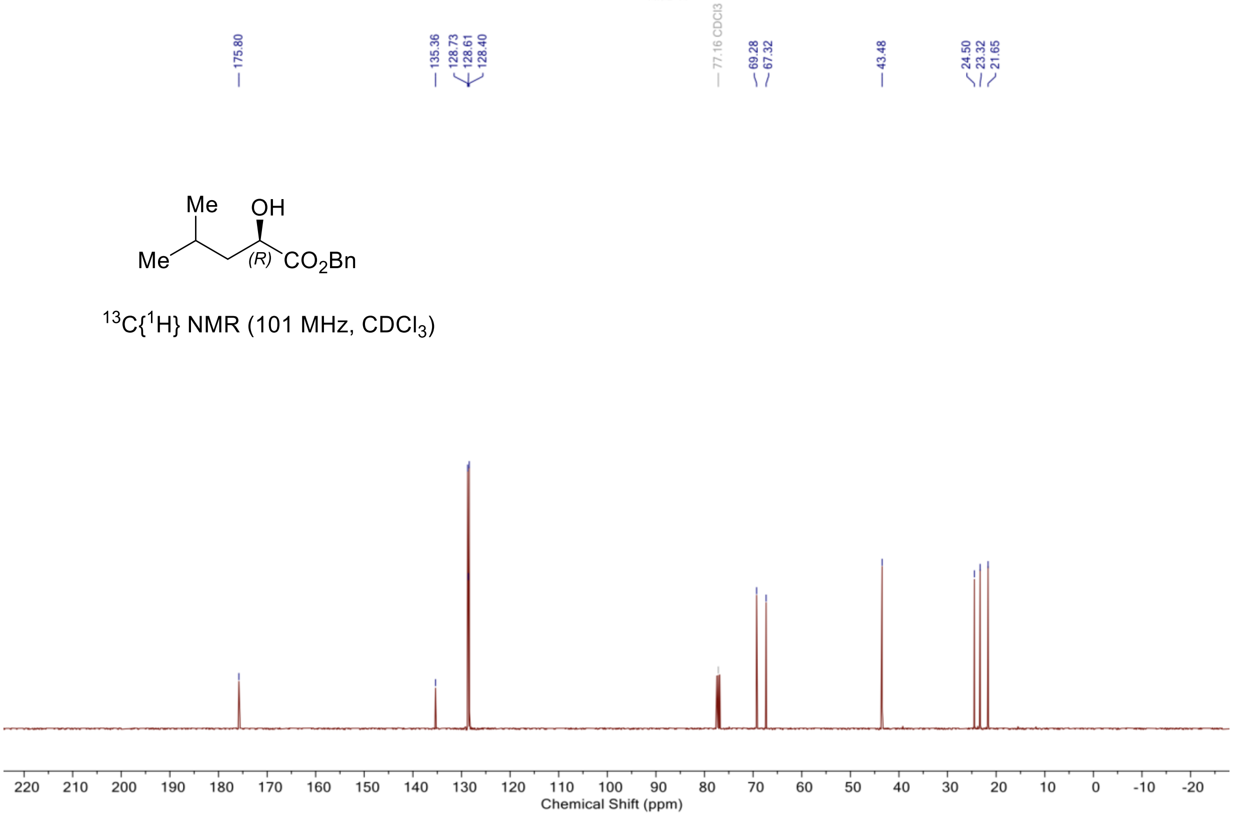

Benzyl (*S*)-2-hydroxy-4-methylpentanoate ((*S*)-7)

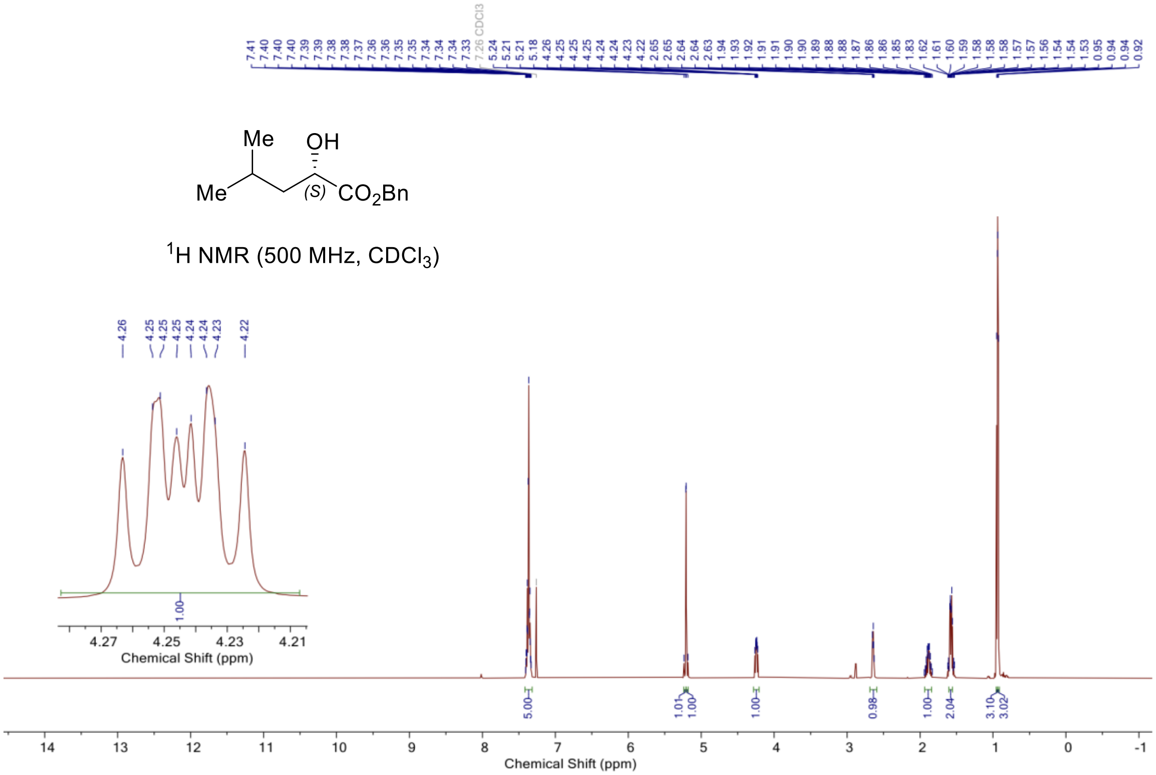

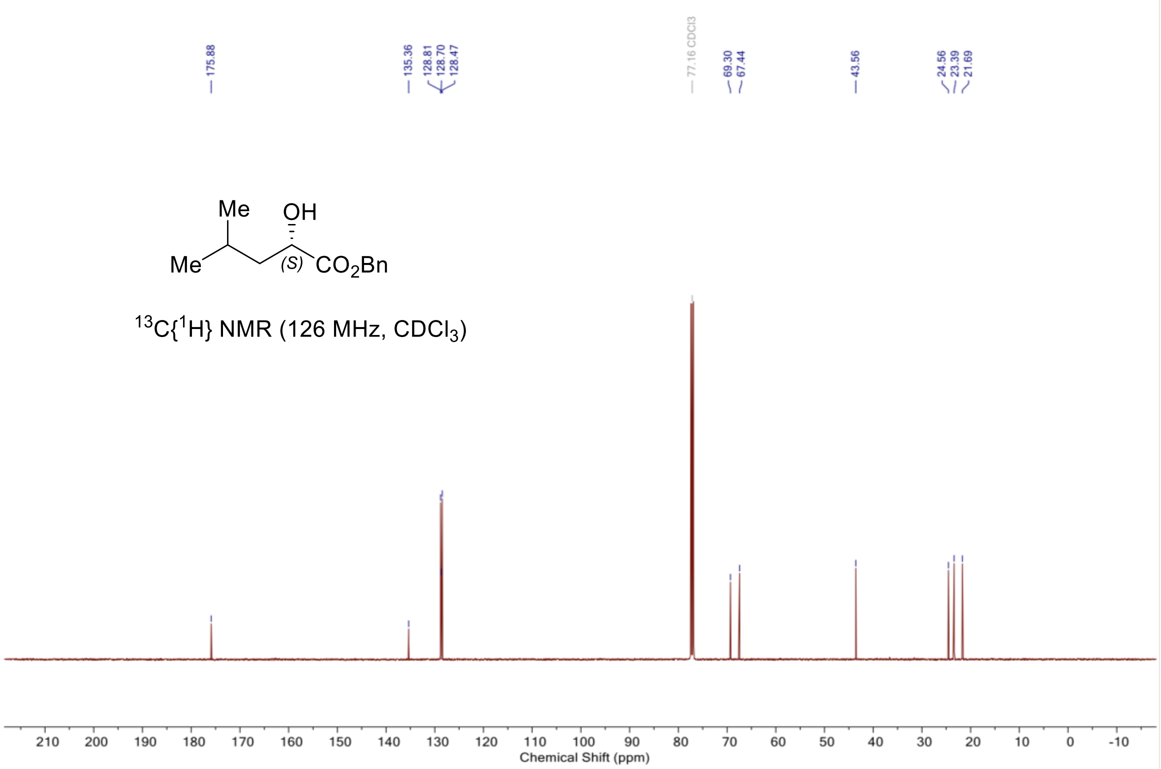

Benzyl *N*-((*S*)-1-methoxy-3-(1-((4-nitrophenyl)sulfonyl)-1*H*-imidazol-4-yl)-1-oxopropan-2-yl)-*N*-((4-nitrophenyl)sulfonyl)-*L*-leucinate ((*S*,*S*)-8)

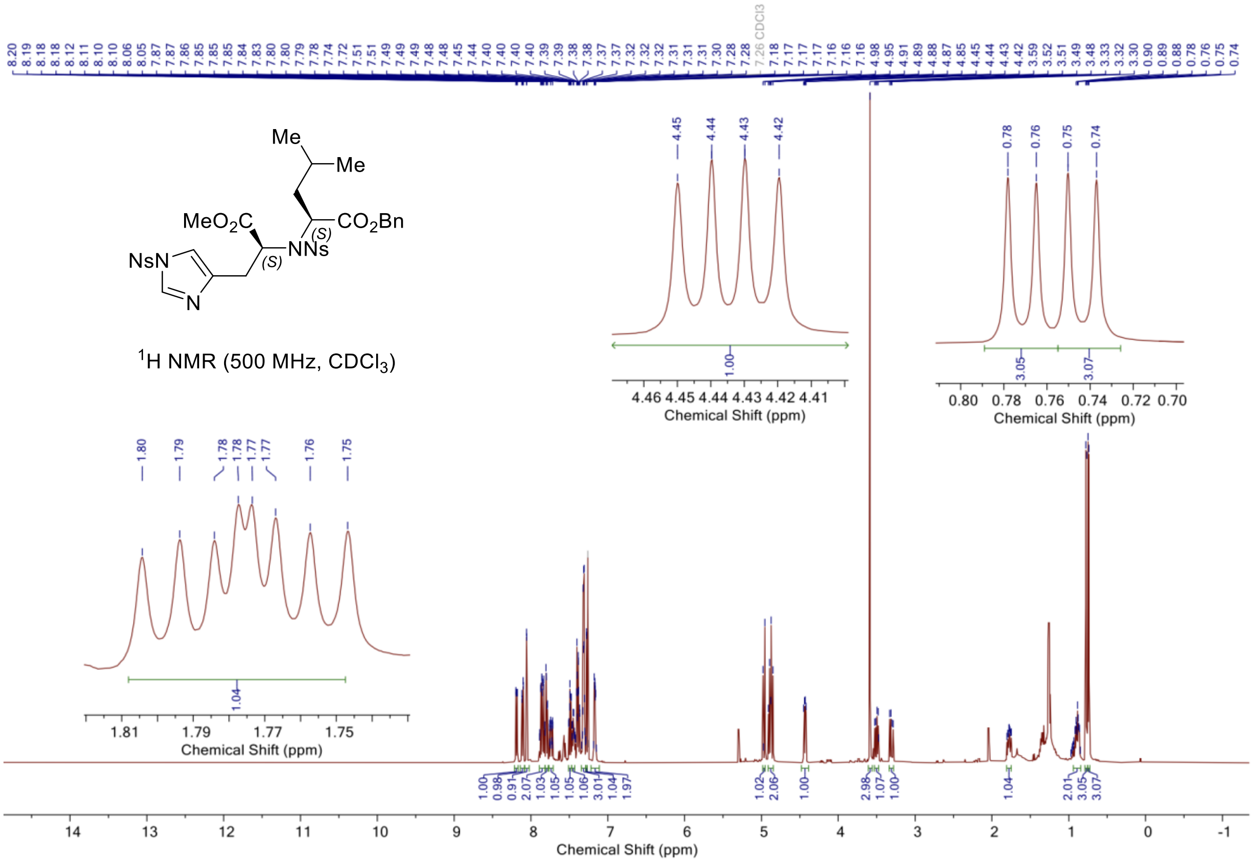

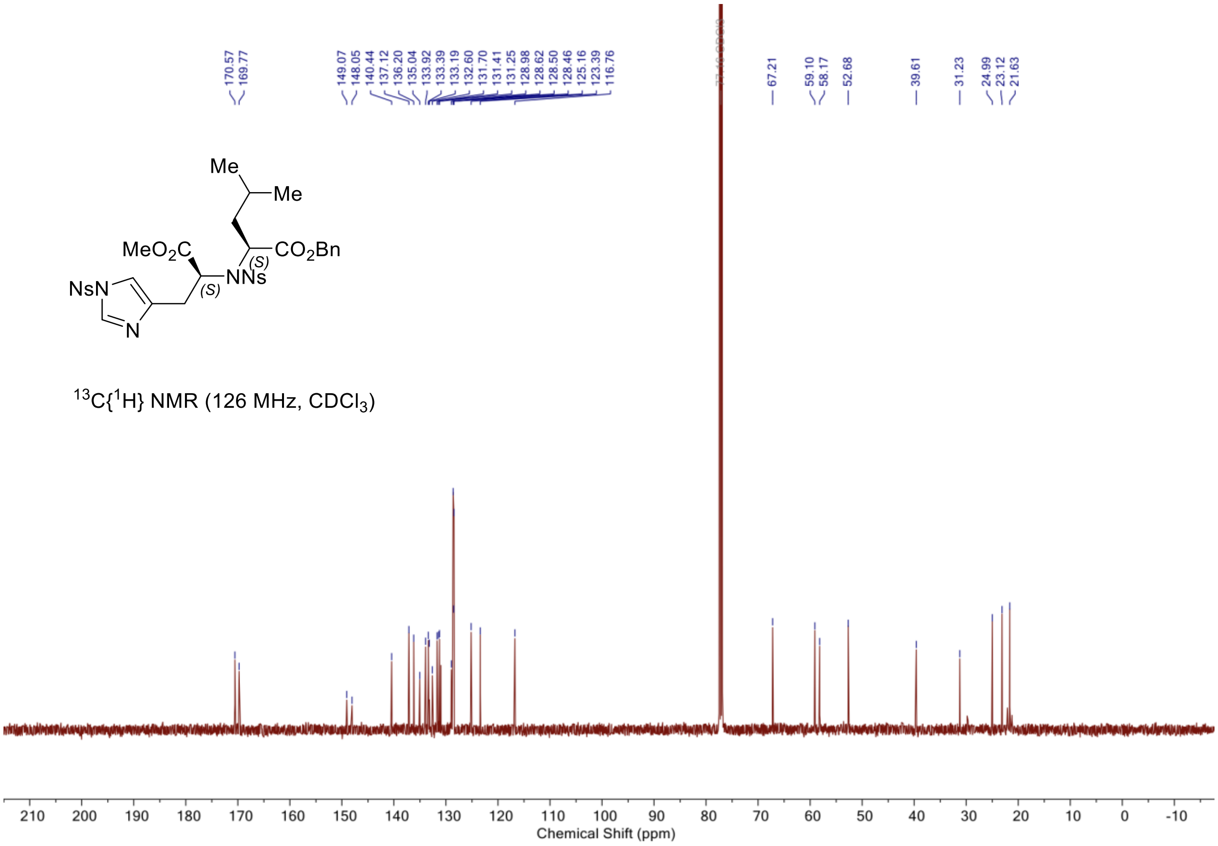

170.57
169.77
149.07
148.05
140.44
137.12
136.20
135.04
133.92
133.39
133.19
132.60
131.70
131.41
131.25
128.98
128.62
128.50
128.46
125.16
123.39
116.76
67.21
59.10
58.17
52.68
39.61
31.23
24.99
23.12
21.63
Me
Me
MeO2C
CO2Bn
(S)
(S)
NNs
NsN
N
13C{1H} NMR (126 MHz, CDCl3)
Chemical Shift (ppm)

Benzyl ((*S*)-3-(1*H*-imidazol-4-yl)-1-methoxy-1-oxopropan-2-yl)-*L*-leucinate ((*S*,*S*)-9)

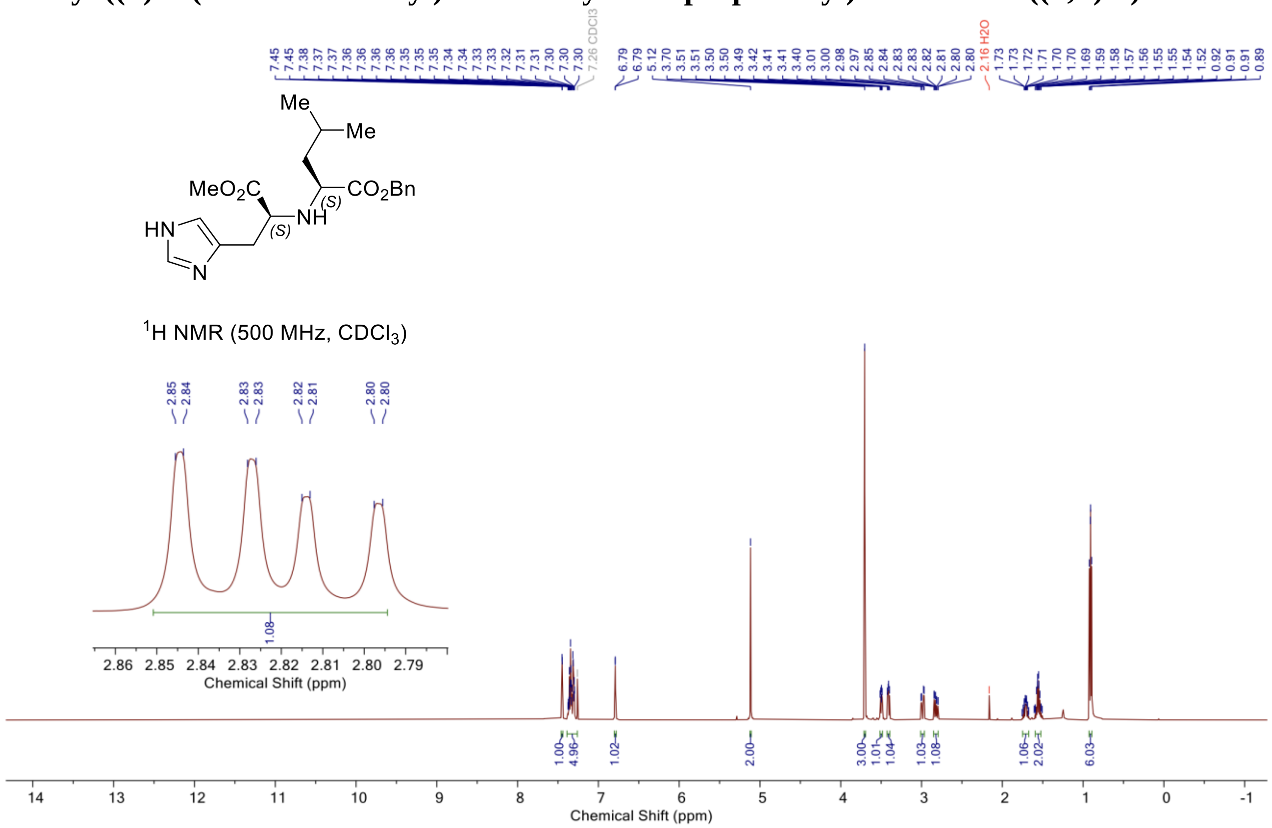

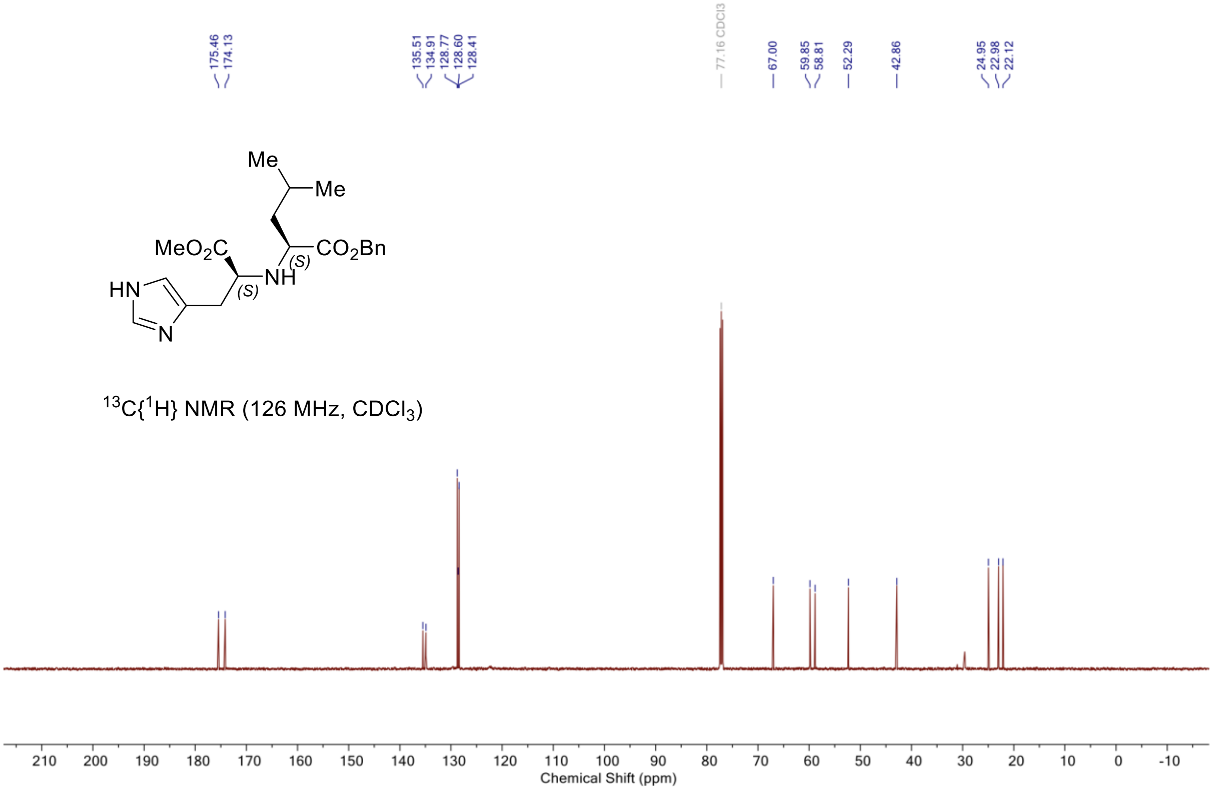

Benzyl ((*S*)-3-(1-(4-fluorobenzyl)-1*H*-imidazol-5-yl)-1-methoxy-1-oxopropan-2-yl)-*L*-leucinate ((*S*,*S*)-11)

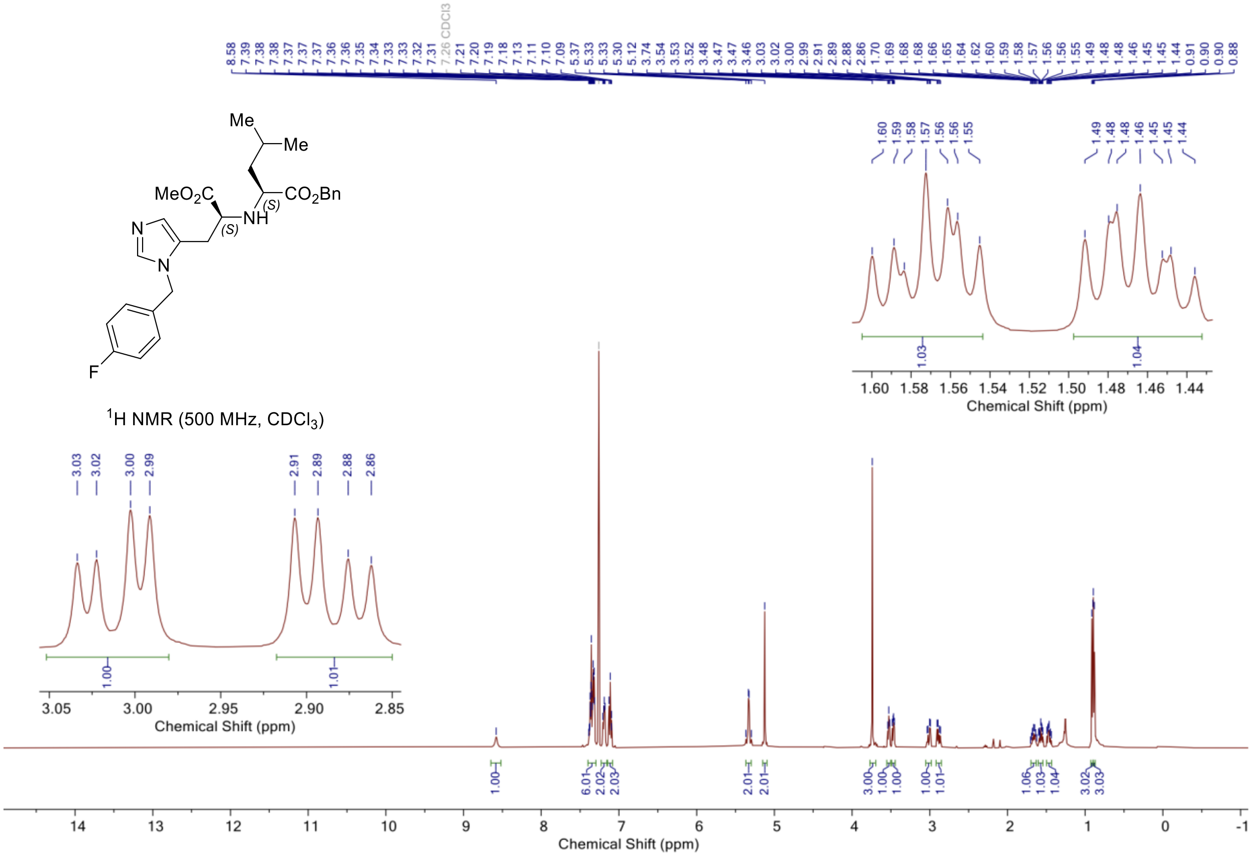

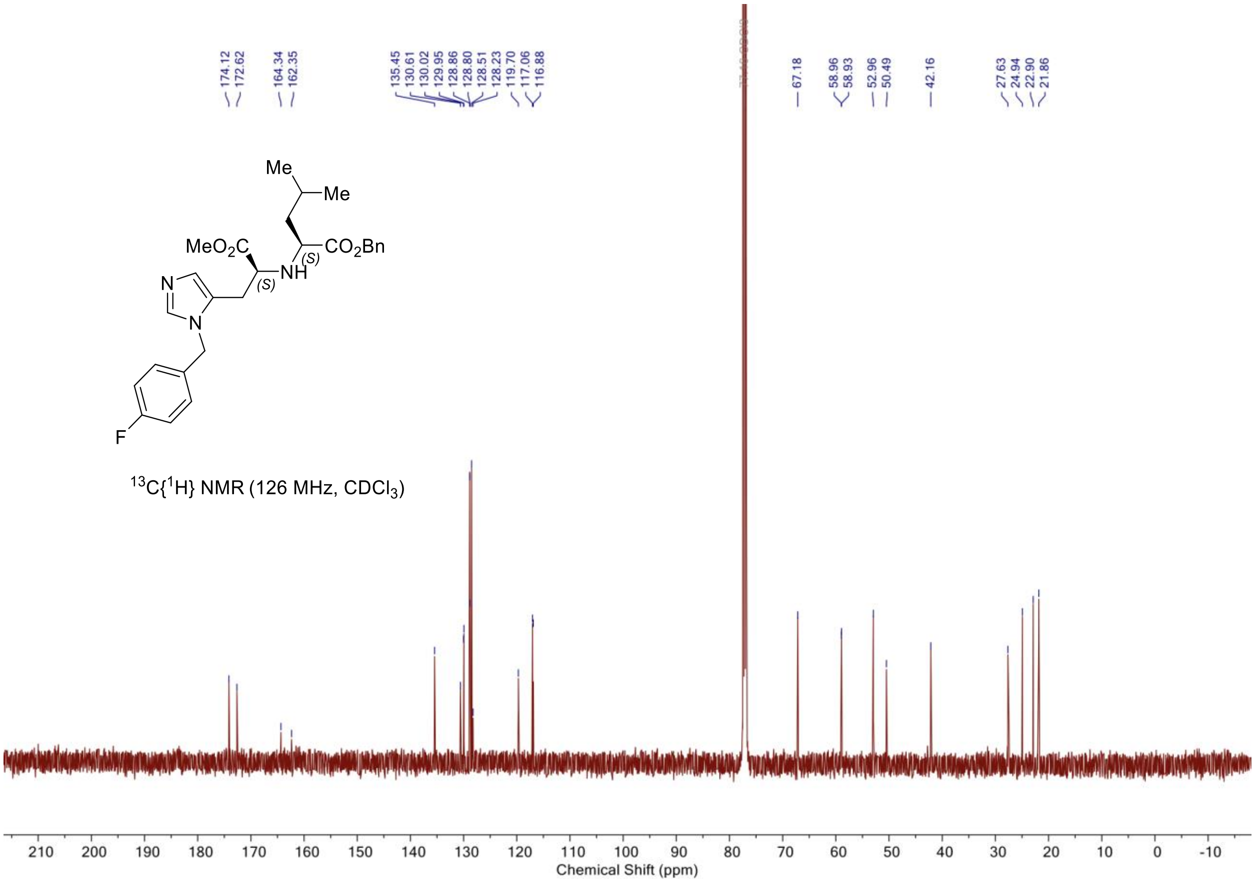
174.12
172.62
164.34
162.35
135.45
130.61
130.02
129.95
128.86
128.80
128.51
128.23
119.70
117.06
116.88
67.18
58.96
58.93
52.96
50.49
42.16
27.63
24.94
22.90
21.86
Me
Me
MeO2C
CO2Bn
(S)
(S)
NH
N
N
F
13C{1H} NMR (126 MHz, CDCl3)
210 200 190 180 170 160 150 140 130 120 110 100 90 80 70 60 50 40 30 20 10 0 -10
Chemical Shift (ppm)

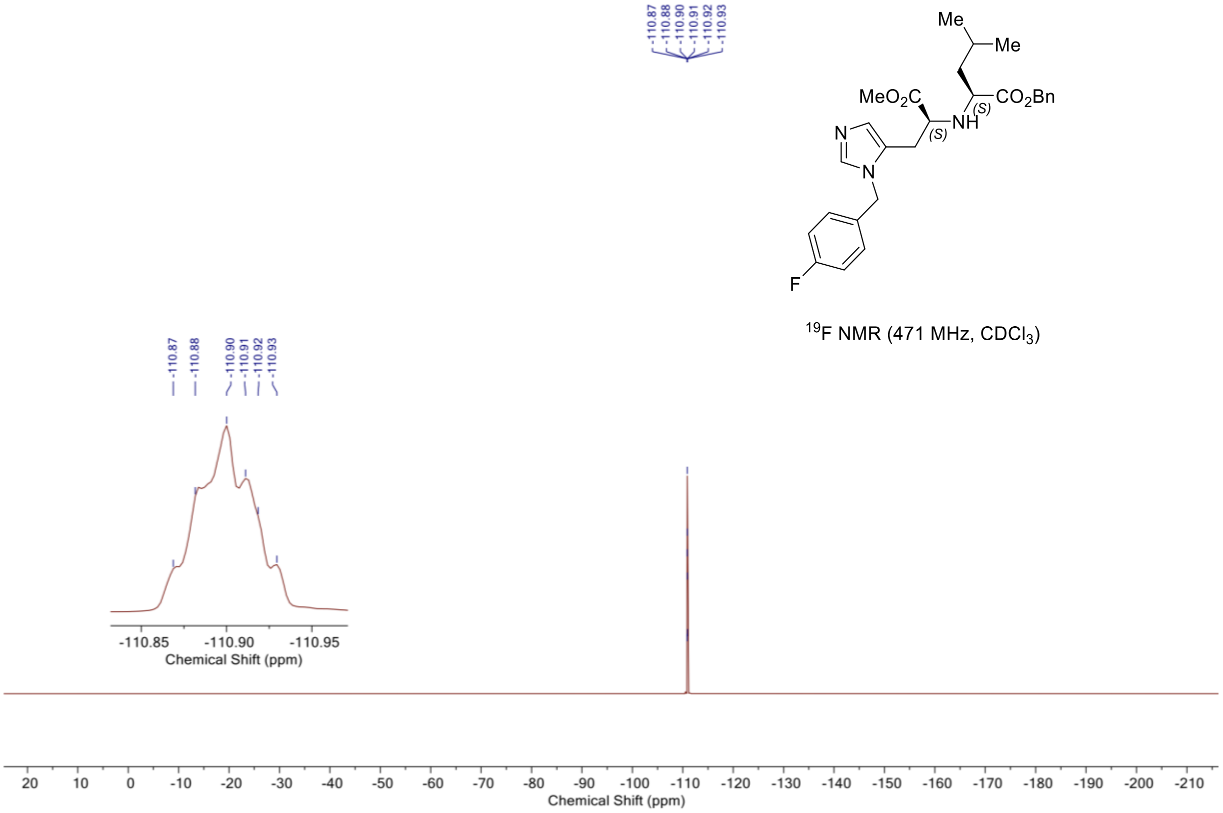
-110.87
-110.88
-110.90
-110.91
-110.92
-110.93
Me
Me
MeO2C
CO2Bn
(S)
(S)
NH
N
N
F
19F NMR (471 MHz, CDCl3)
-110.85 -110.90 -110.95
Chemical Shift (ppm)
20 10 0 -10 -20 -30 -40 -50 -60 -70 -80 -90 -100 -110 -120 -130 -140 -150 -160 -170 -180 -190 -200 -210
Chemical Shift (ppm)

Benzyl ((*S*)-3-(1-(4-fluorobenzyl)-1*H*-imidazol-5-yl)-1-methoxy-1-oxopropan-2-yl)-*D*-leucinate ((*S*,*R*)-11)

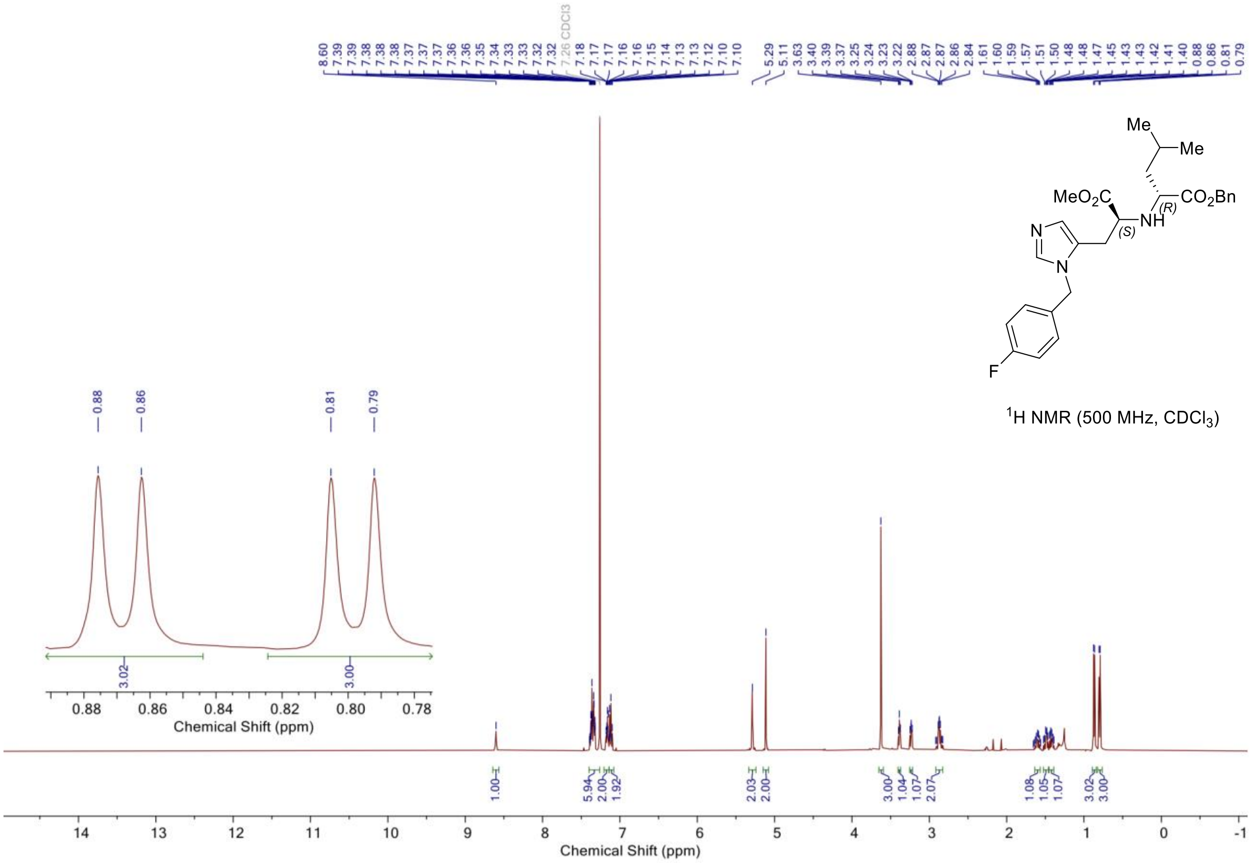

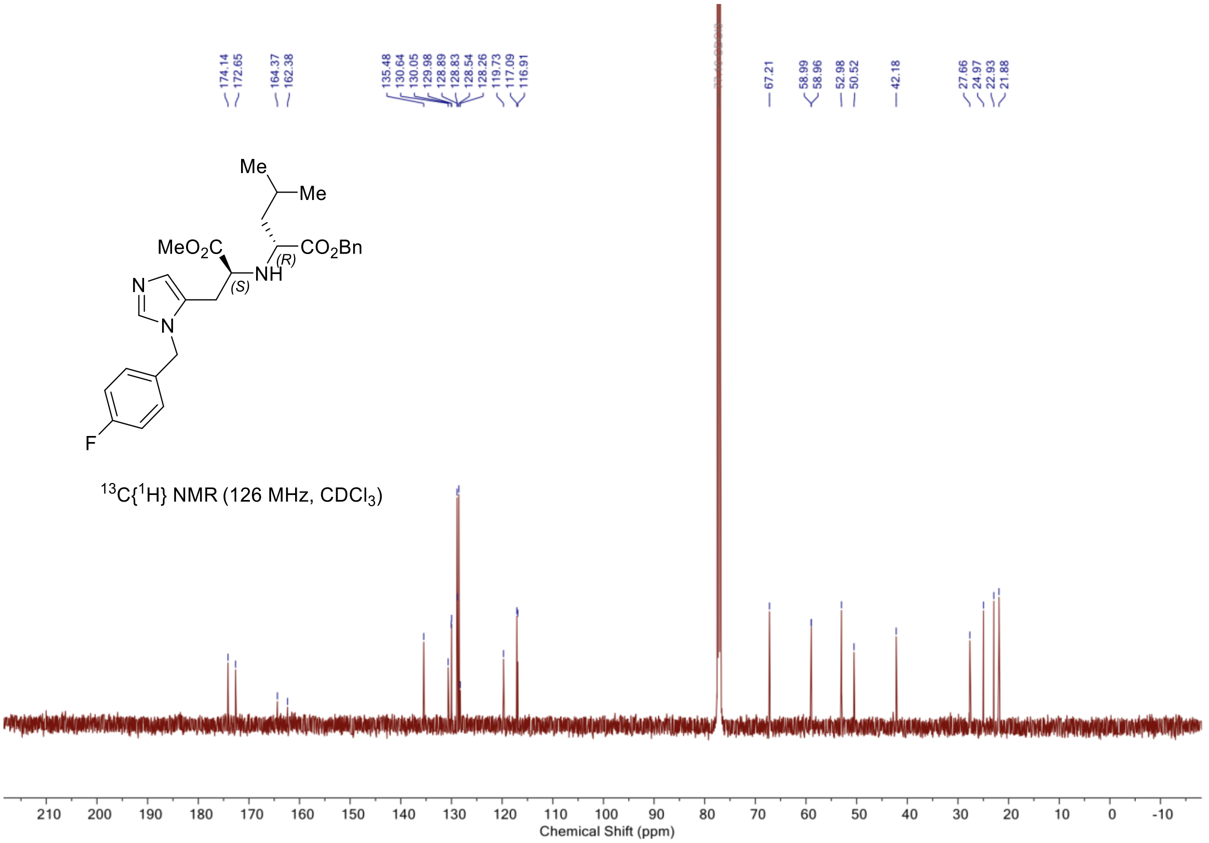
174.14
172.65
164.37
162.38
135.48
130.64
130.05
129.98
128.89
128.83
128.54
128.26
119.73
117.09
116.91
67.21
58.99
58.96
52.98
50.52
42.18
27.66
24.97
22.93
21.88
Me
Me
MeO2C
CO2Bn
(R)
NH
(S)
N
N
F
13C{1H} NMR (126 MHz, CDCl3)
Chemical Shift (ppm)

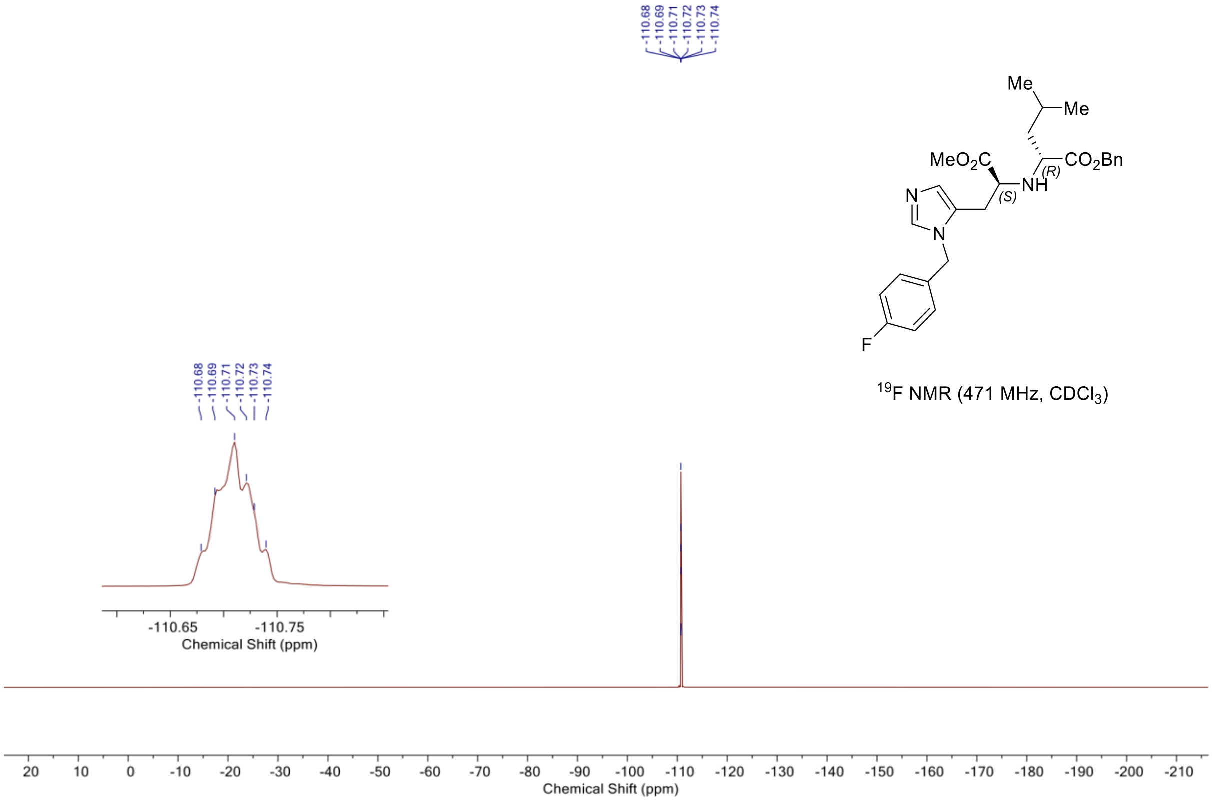
-110.68
-110.69
-110.71
-110.72
-110.73
-110.74
Me
Me
MeO2C
CO2Bn
(R)
NH
(S)
N
N
F
19F NMR (471 MHz, CDCl3)
-110.65
-110.75
Chemical Shift (ppm)
Chemical Shift (ppm)

(3-(2-Fluoroethoxy)-5-methyoxyphenyl)methanol (14)

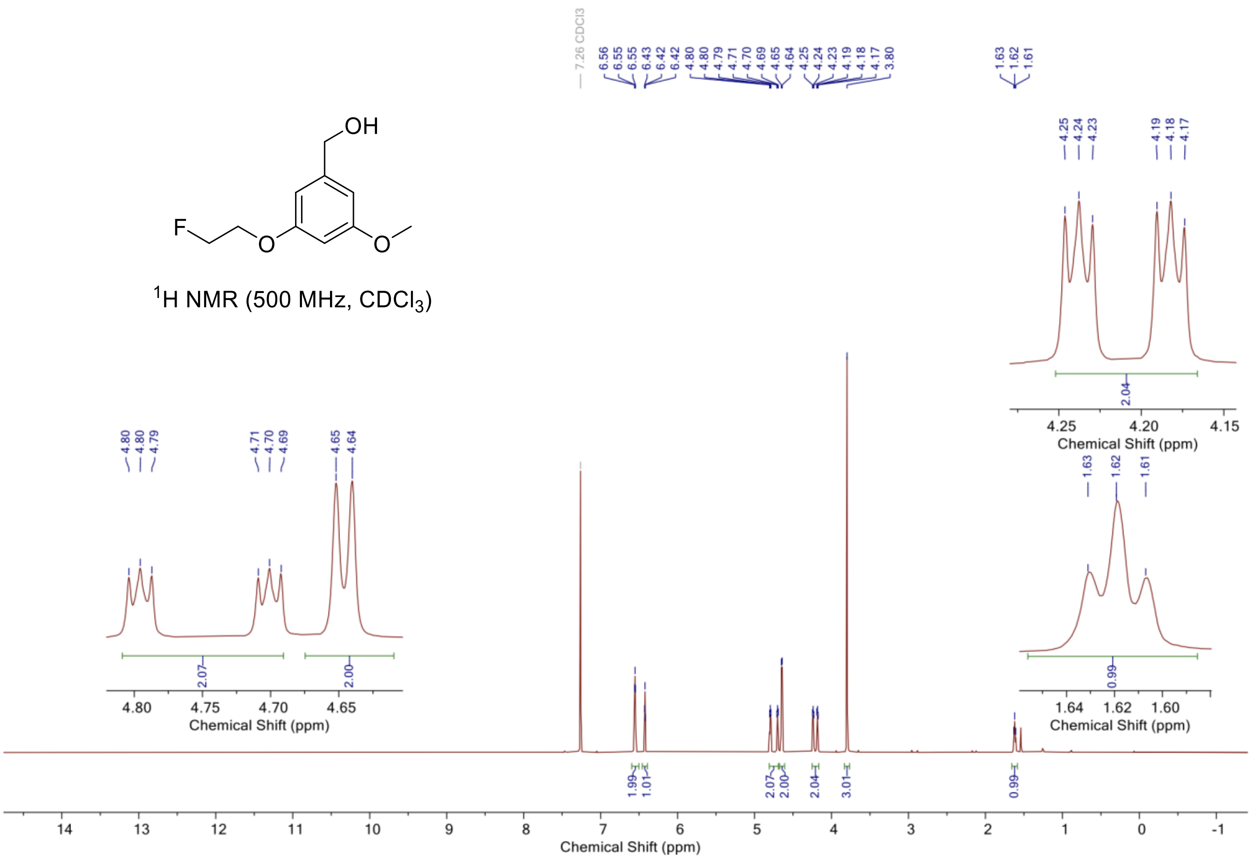

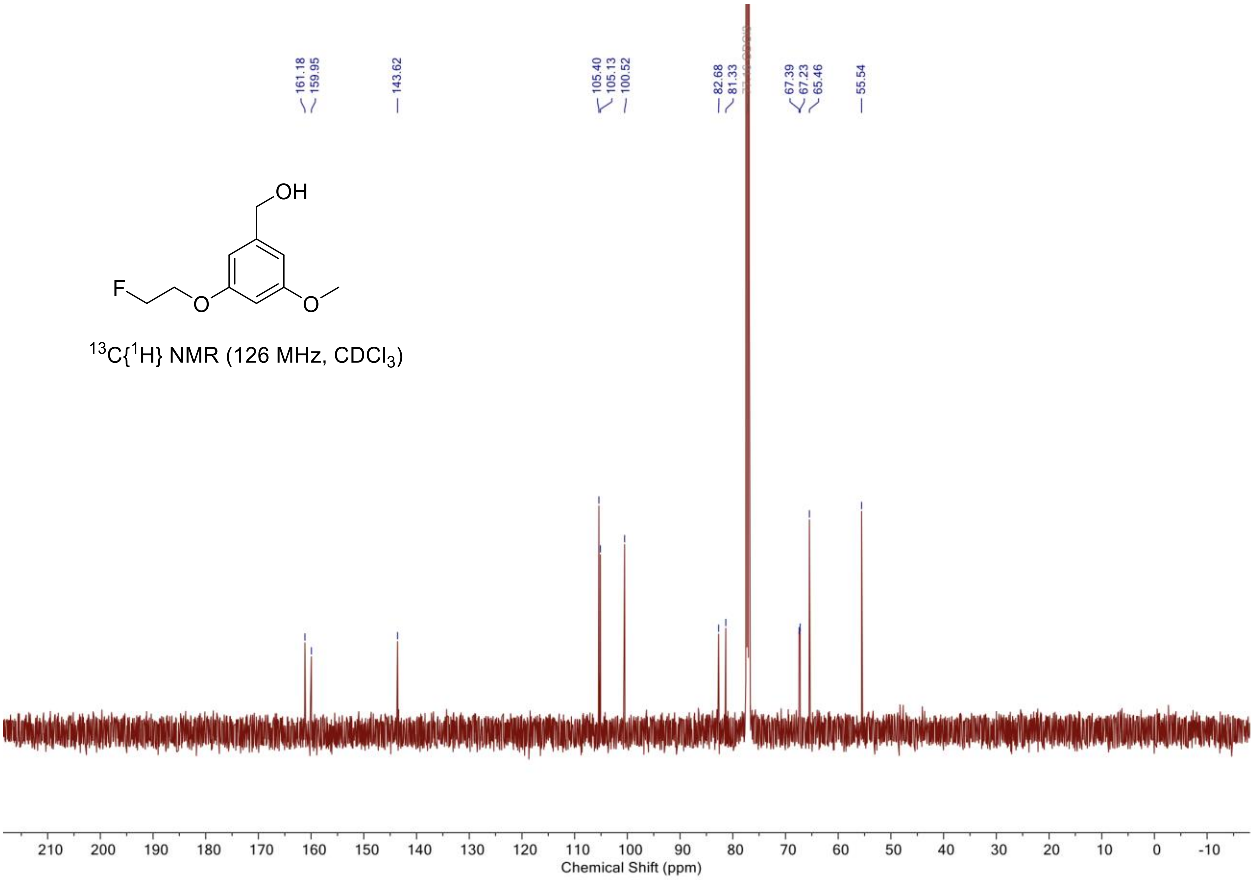
161.18
159.95
143.62
105.40
105.13
100.52
82.68
81.33
67.39
67.23
65.46
55.54
OH
F
O
O
13C{1H} NMR (126 MHz, CDCl3)
210 200 190 180 170 160 150 140 130 120 110 100 90 80 70 60 50 40 30 20 10 0 -10
Chemical Shift (ppm)

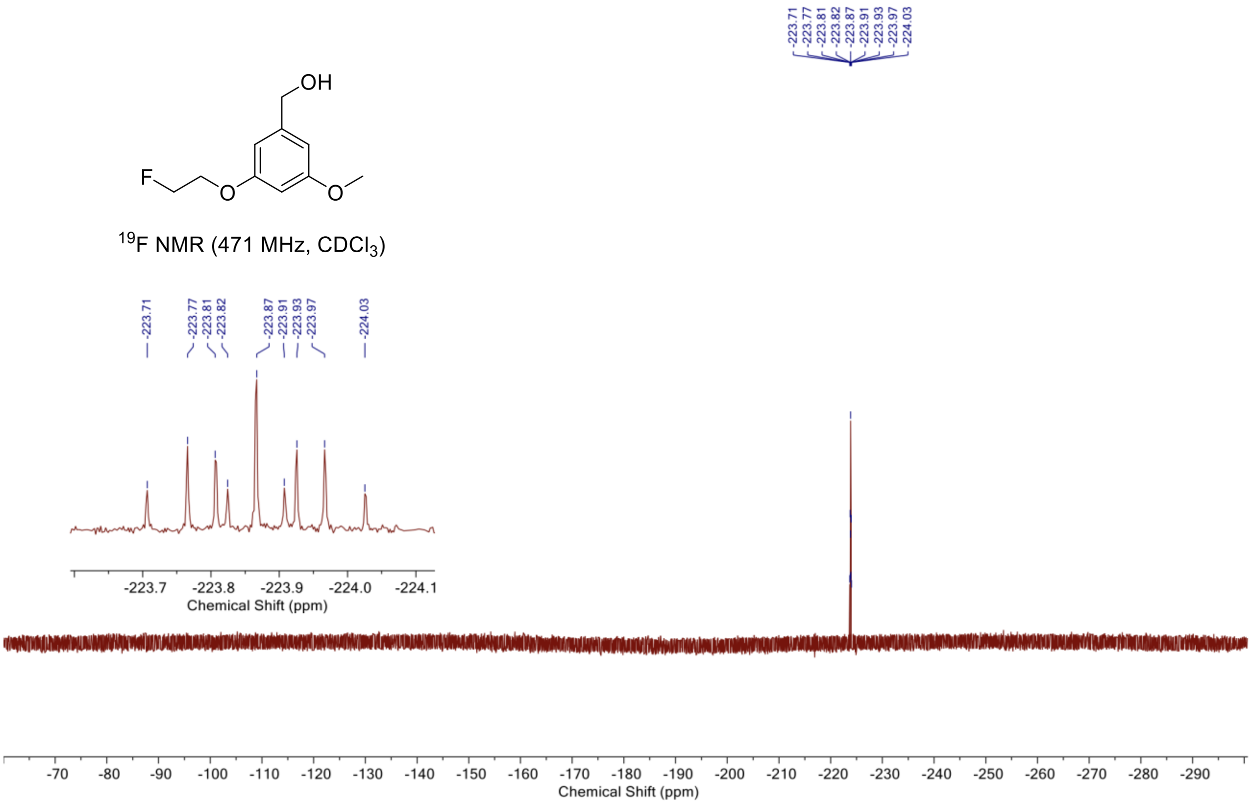
-223.71
-223.77
-223.81
-223.82
-223.87
-223.91
-223.93
-223.97
-224.03
OH
F
O
O
19F NMR (471 MHz, CDCl3)
-223.7 -223.8 -223.9 -224.0 -224.1
Chemical Shift (ppm)
-70 -80 -90 -100 -110 -120 -130 -140 -150 -160 -170 -180 -190 -200 -210 -220 -230 -240 -250 -260 -270 -280 -290
Chemical Shift (ppm)

Benzyl ((*S*)-3-(1-(3-fluoro-5-methylbenzyl)-1*H*-imidazol-5-yl)-1-methoxy-1-oxopropan-2-yl)-*L*-leucinate ((*S*,*S*)-15)

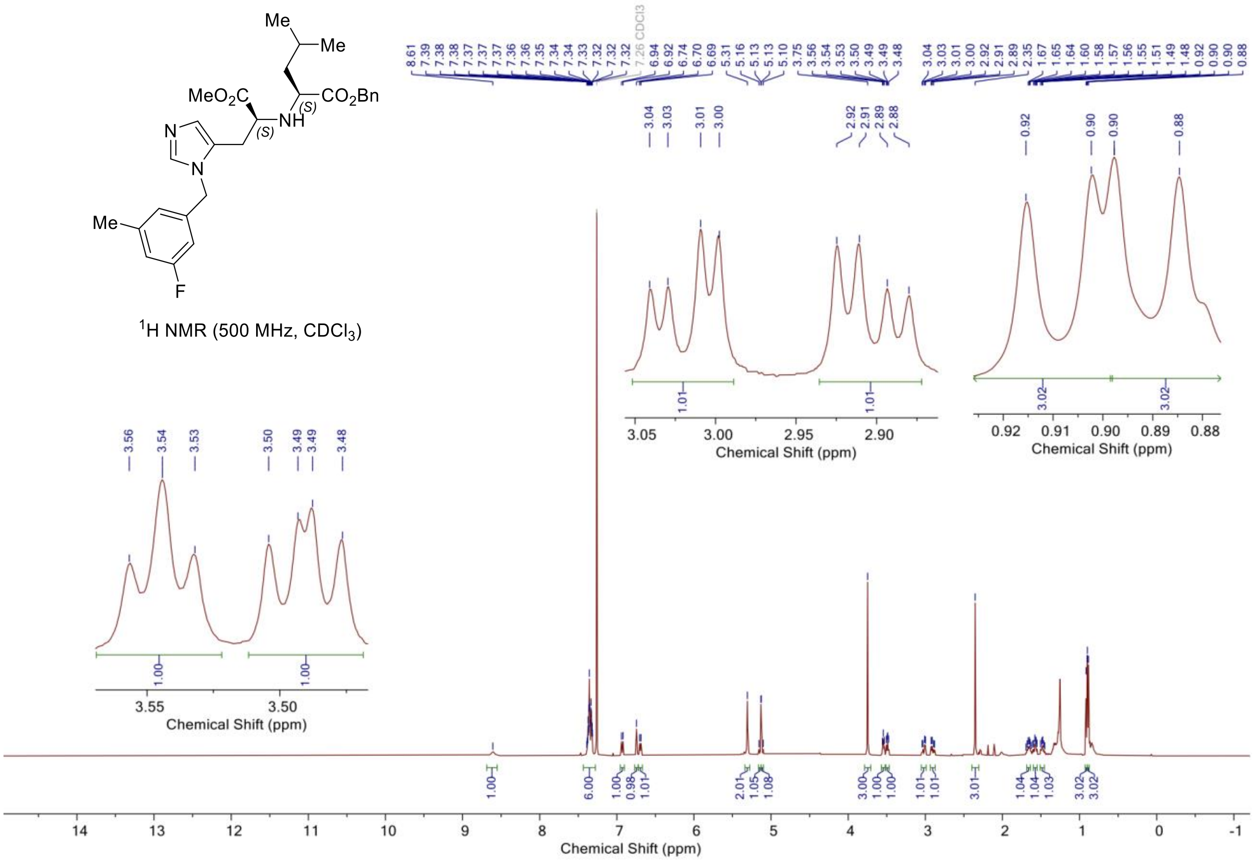

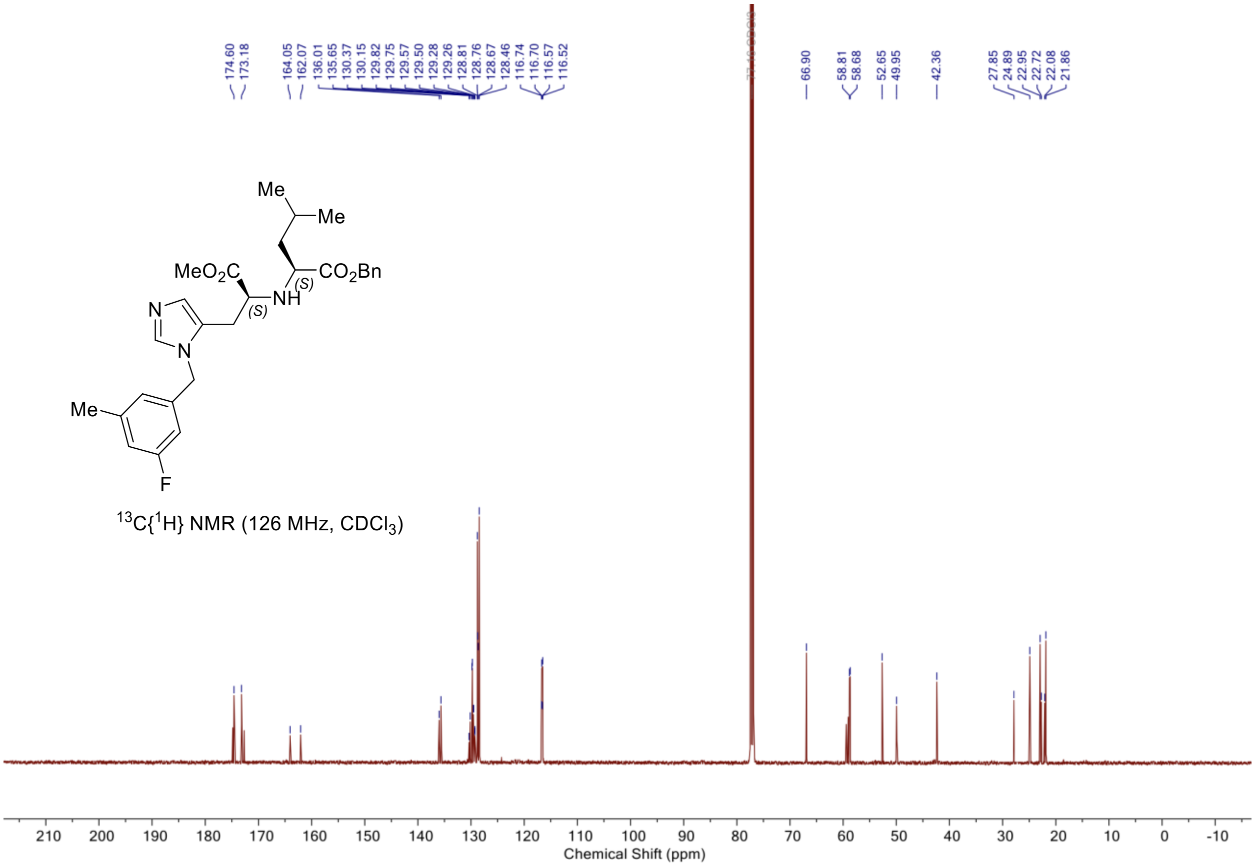
174.60
173.18
164.05
162.07
136.01
135.65
130.37
130.15
129.82
129.75
129.57
129.50
129.28
129.26
128.81
128.76
128.67
128.46
116.74
116.70
116.57
116.52
66.90
58.81
58.68
52.65
49.95
42.36
27.85
24.89
22.95
22.72
22.08
21.86
Me
Me
MeO2C
CO2Bn
(S)
NH
(S)
N
N
Me
F
13C{1H} NMR (126 MHz, CDCl3)
210 200 190 180 170 160 150 140 130 120 110 100 90 80 70 60 50 40 30 20 10 0 -10
Chemical Shift (ppm)

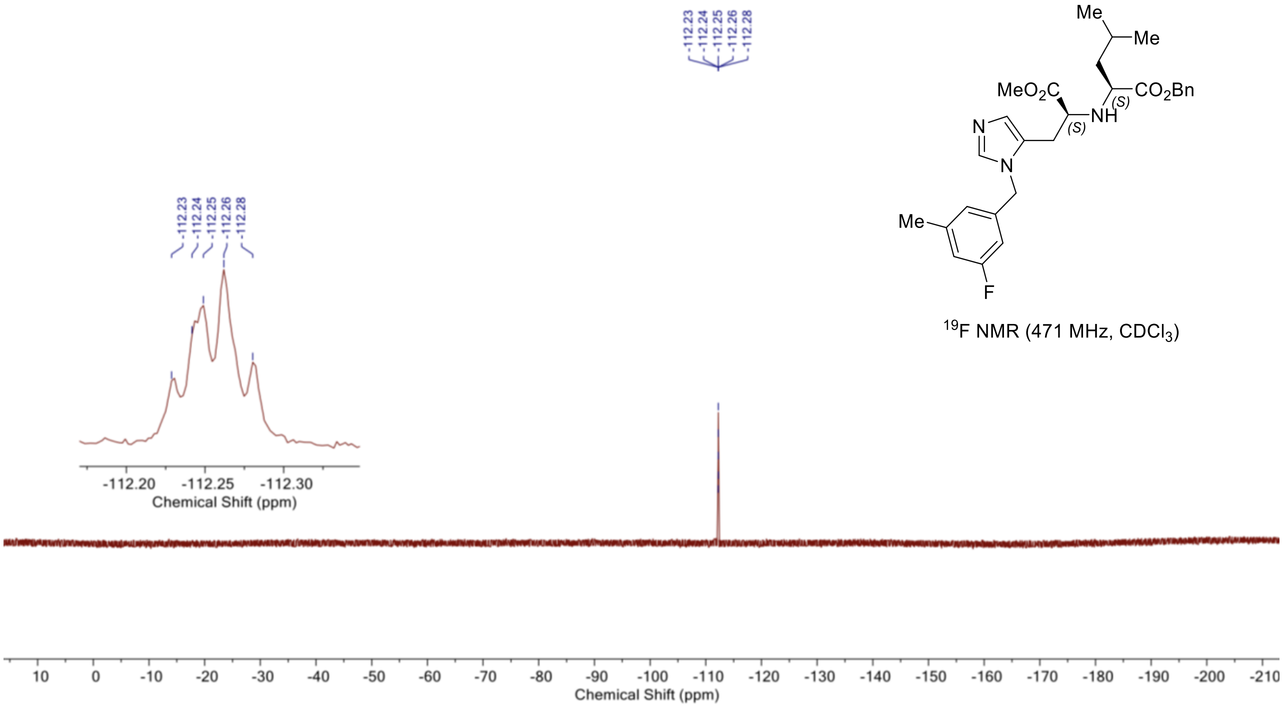
-112.23
-112.24
-112.25
-112.26
-112.28
Me
Me
MeO2C
CO2Bn
(S)
NH
(S)
N
N
Me
F
19F NMR (471 MHz, CDCl3)
-112.20 -112.25 -112.30
Chemical Shift (ppm)
10 0 -10 -20 -30 -40 -50 -60 -70 -80 -90 -100 -110 -120 -130 -140 -150 -160 -170 -180 -190 -200 -210
Chemical Shift (ppm)

Benzyl ((*S*)-3-(1-(3-chloro-5-fluorobenzyl)-1*H*-imidazol-5-yl)-1-methoxy-1-oxopropan-2-yl)-*L*-leucinate ((*S*,*S*)-16)

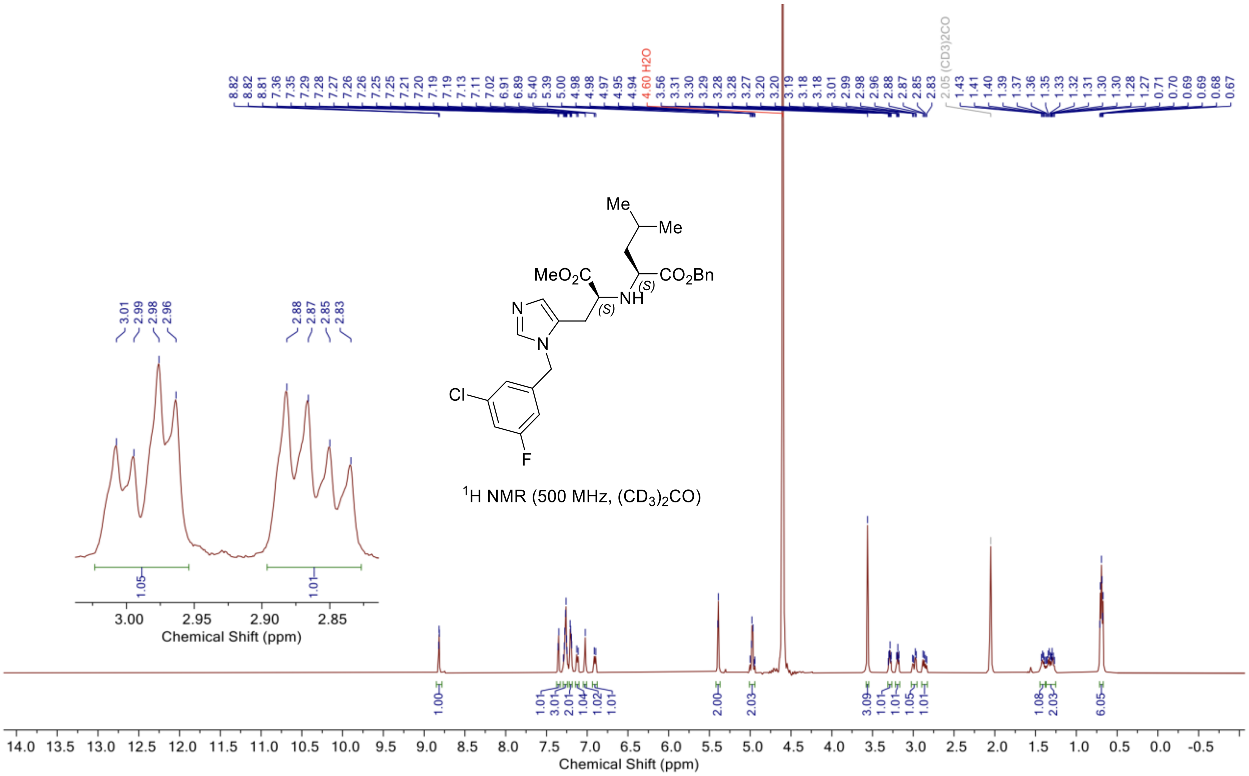

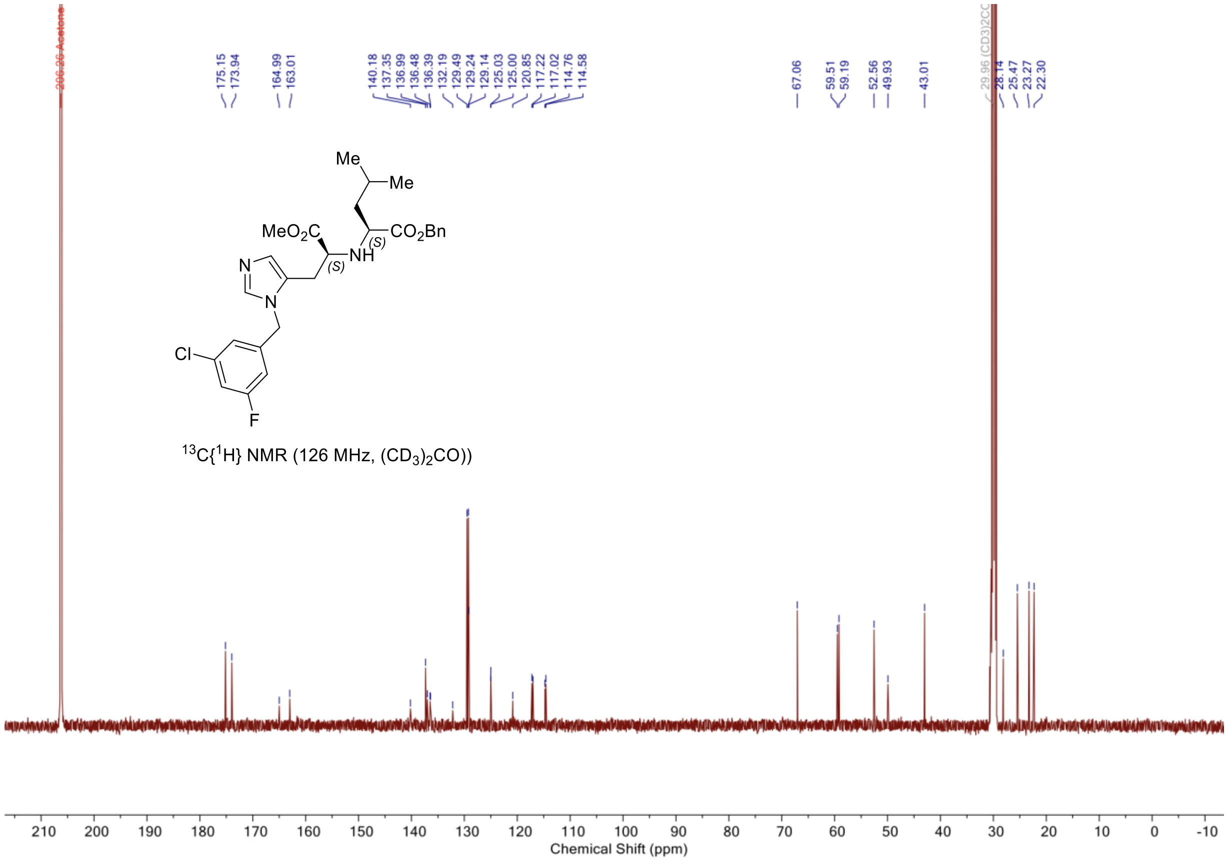

$^{13}C\{^1H\}$ NMR (126 MHz, $(CD_3)_2CO$))

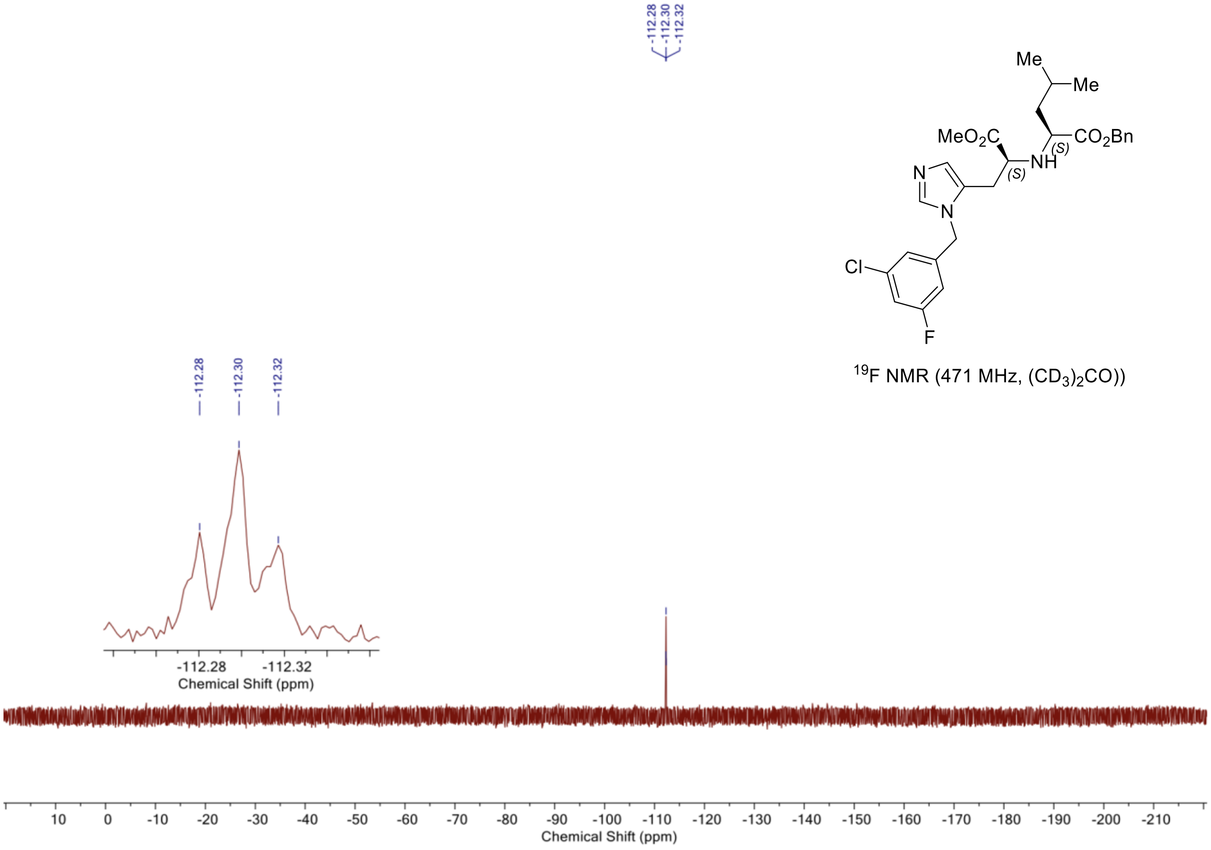

^{19}F NMR (471 MHz, $(CD_3)_2CO$))

Benzyl ((*S*)-3-(1-(3-(2-fluoroethoxy)-5-methoxybenzyl)-1*H*-imidazol-5-yl)-1-methoxy-1-oxopropan-2-yl)-*L*-leucinate ((*S*,*S*)-17)

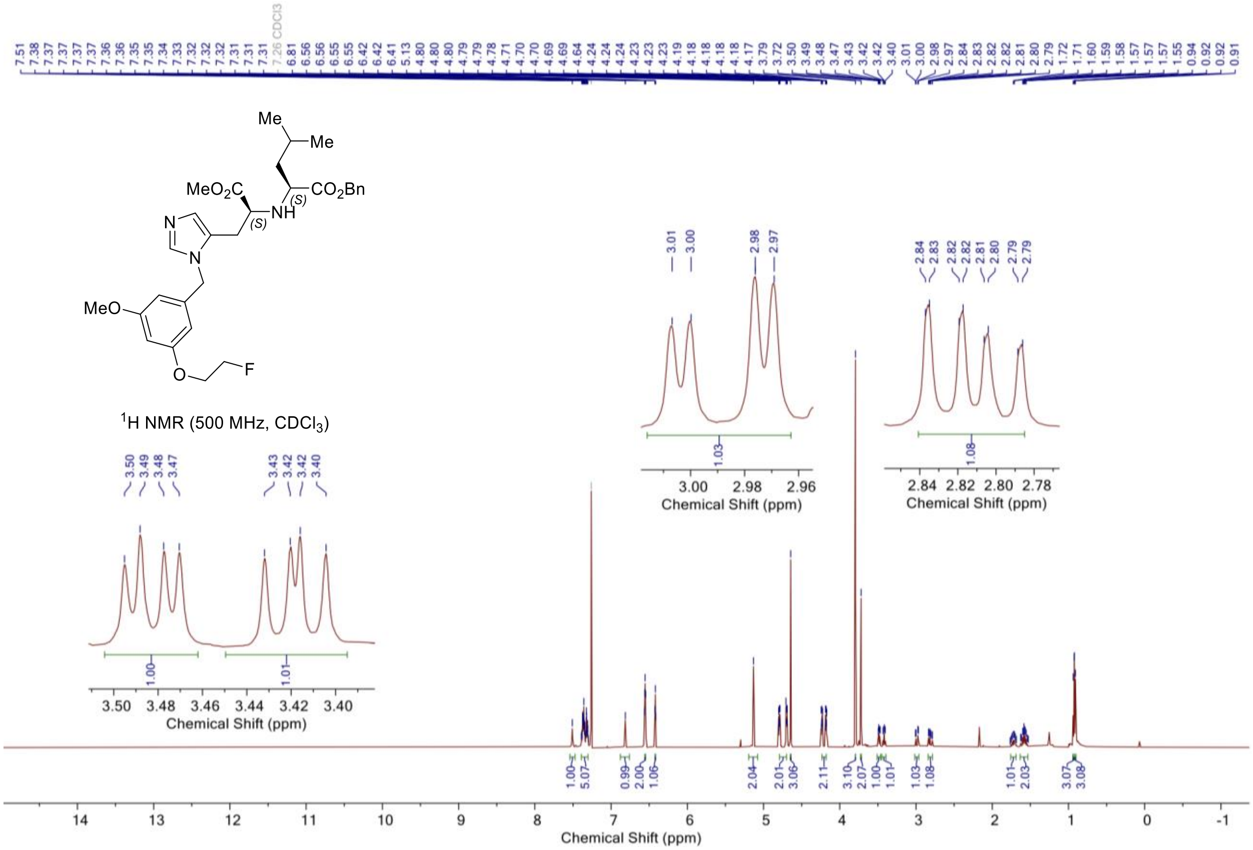

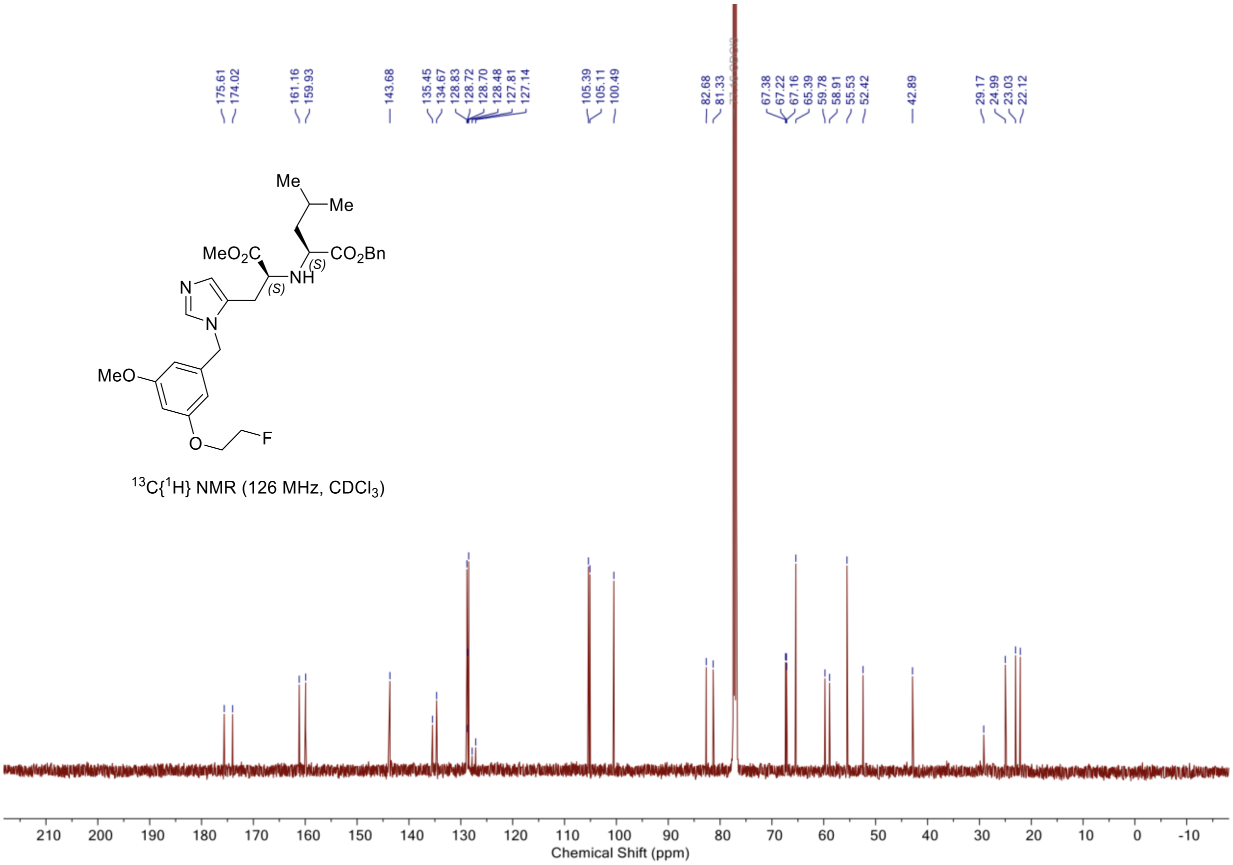

$^{13}C\{^1H\}$ NMR (126 MHz, $CDCl_3$)

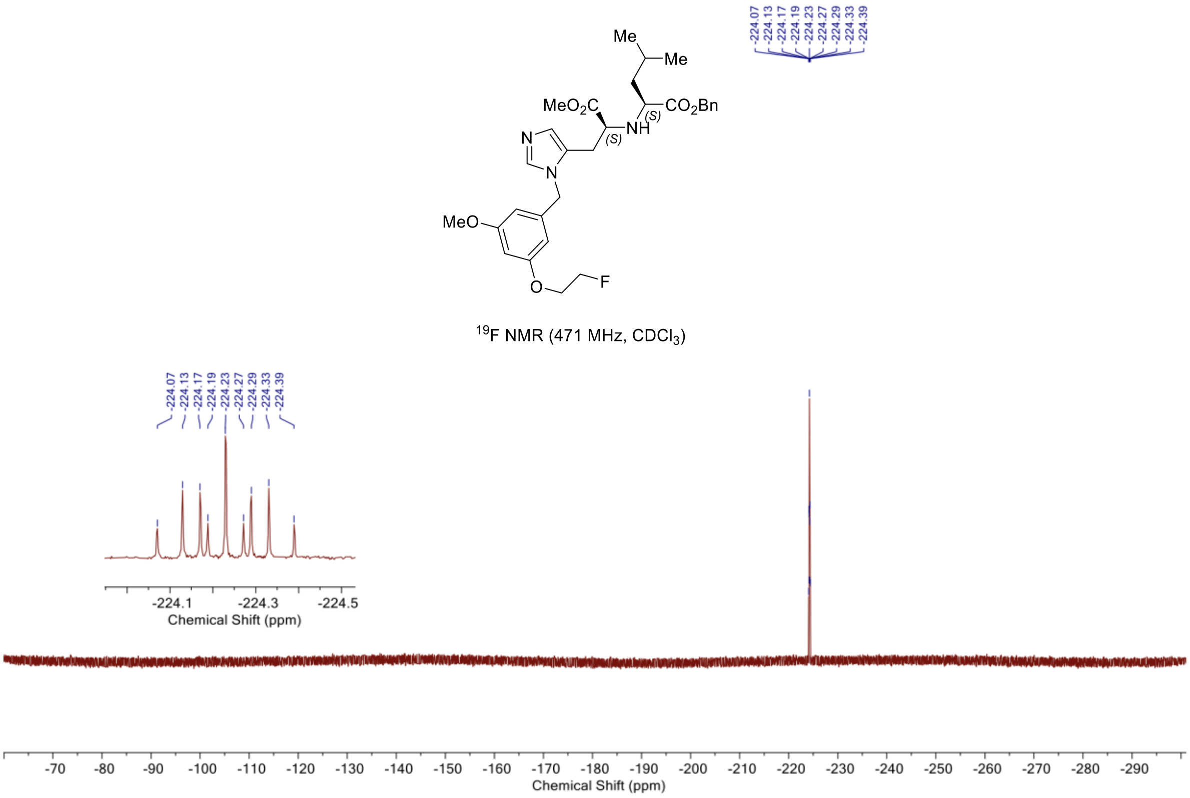

^{19}F NMR (471 MHz, $CDCl_3$)

Benzyl ((*S*)-1-methoxy-1-oxo-3-(1-(4-(4,4,5,5-tetramethyl-1,3,2-dioxaborolan-2-yl)benzyl)-1*H*-imidazol-5-yl)propan-2-yl)-L-leucinate ((*S*, *S*)-18)

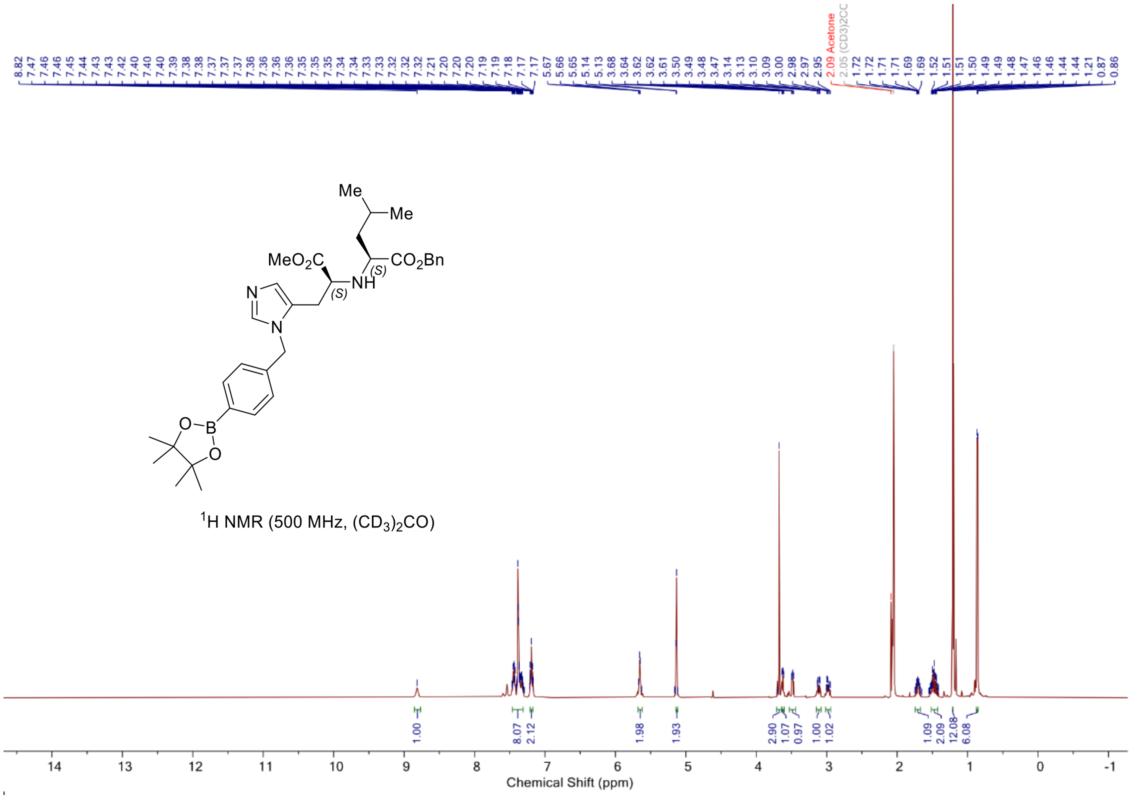

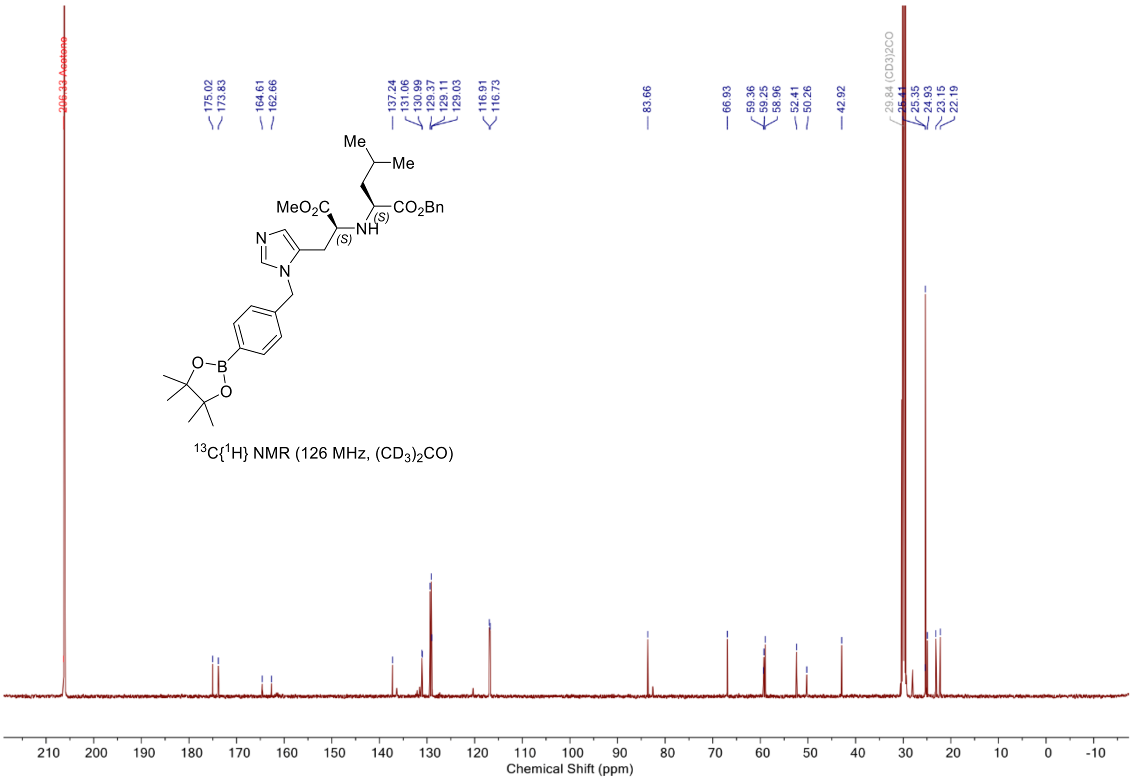

Benzyl ((*S*)-3-(1-(4-bromobenzyl)-1*H*-imidazol-5-yl)-1-methoxy-1-oxopropan-2-yl)-*L*-leucinate ((*S*,*S*)-S9)

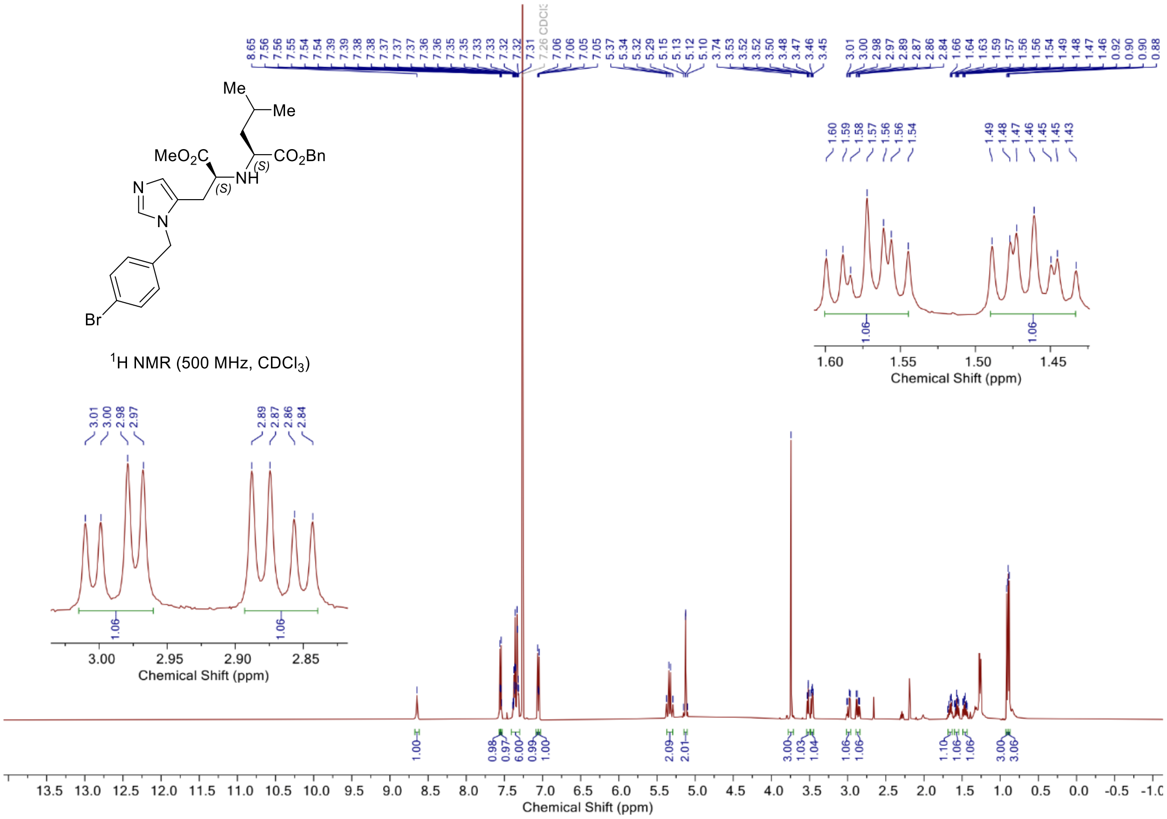

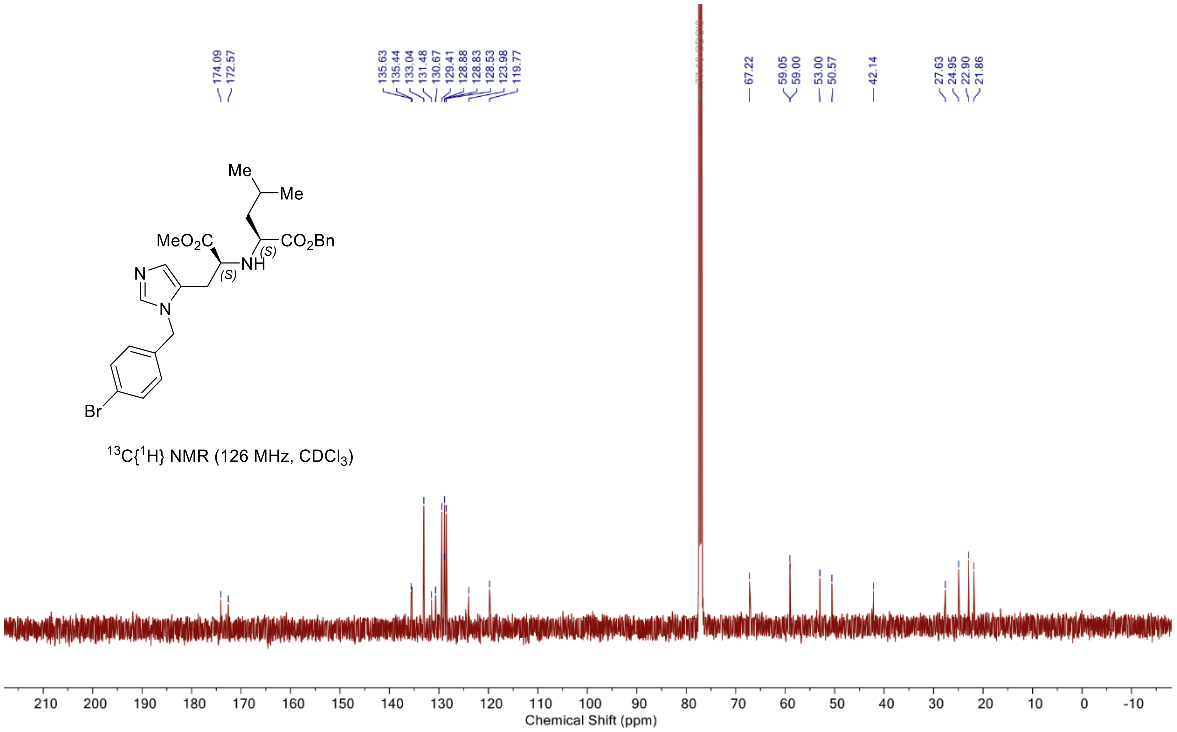

Benzyl ((*S*)-3-(1-(3-chloro-5-(4,4,5,5-tetramethyl-1,3,2-dioxaborolan-2-yl)benzyl)-1*H*-imidazol-5-yl)-1-methoxy-1-oxopropan-2-yl)-*L*-leucinate ((*S*,*S*)-19)

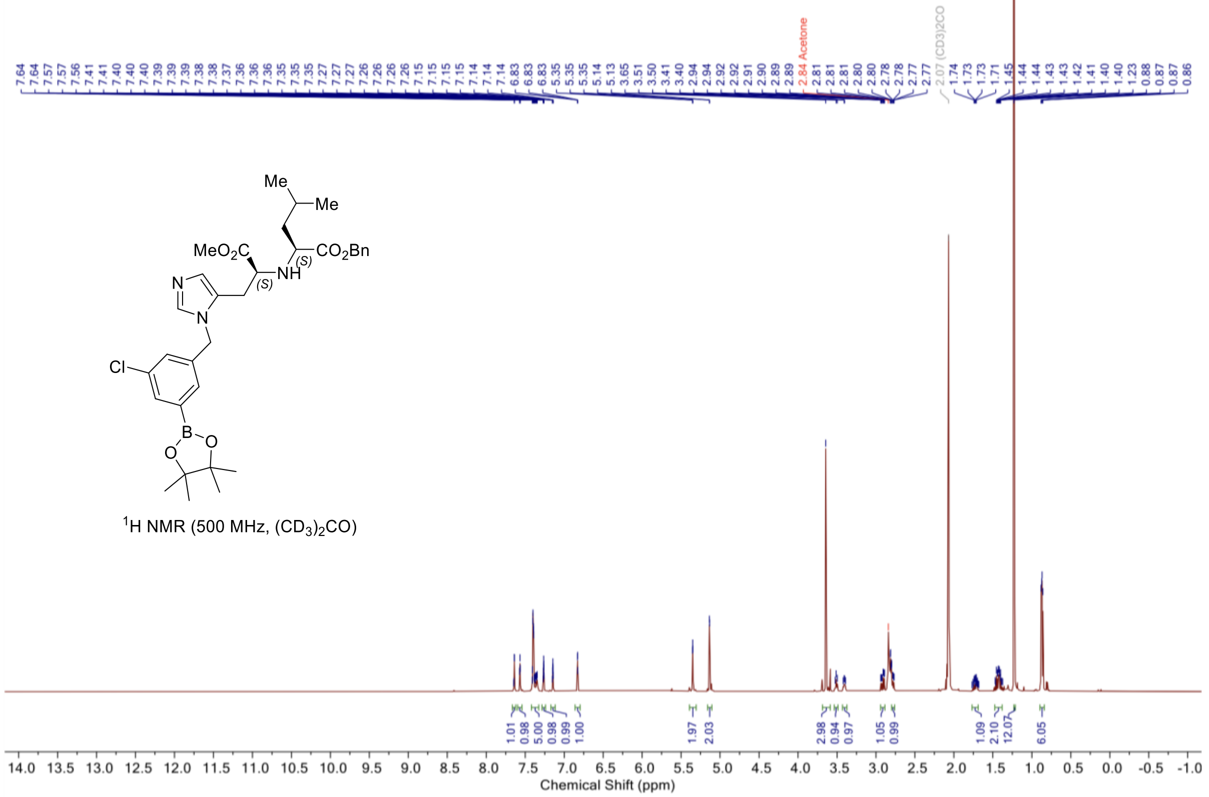

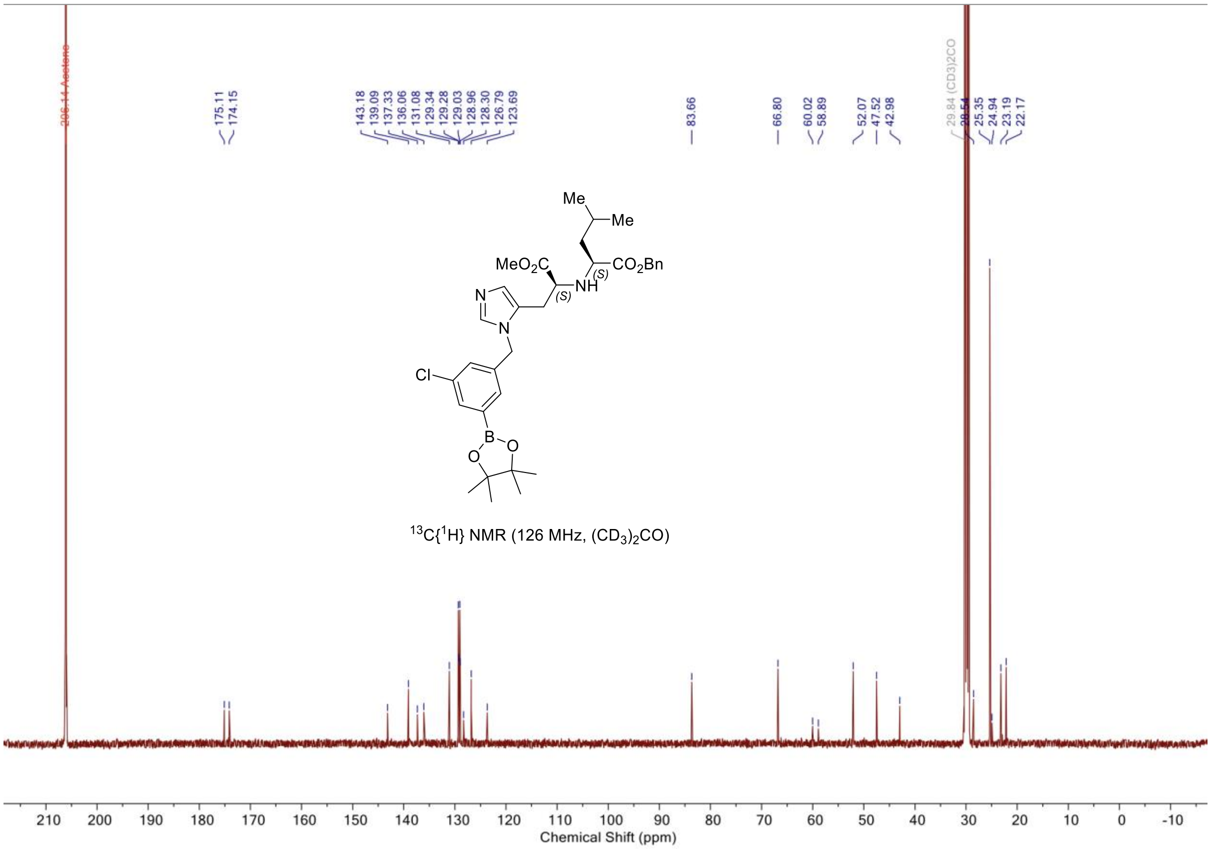

Benzyl ((*S*)- 3-(1-(3-bromo-5-chlorobenzyl)-1*H*-imidazol-5-yl)-1-methoxy-1-oxopropan-2-yl)-*L*-leucinate ((*S*,*S*)-S10)

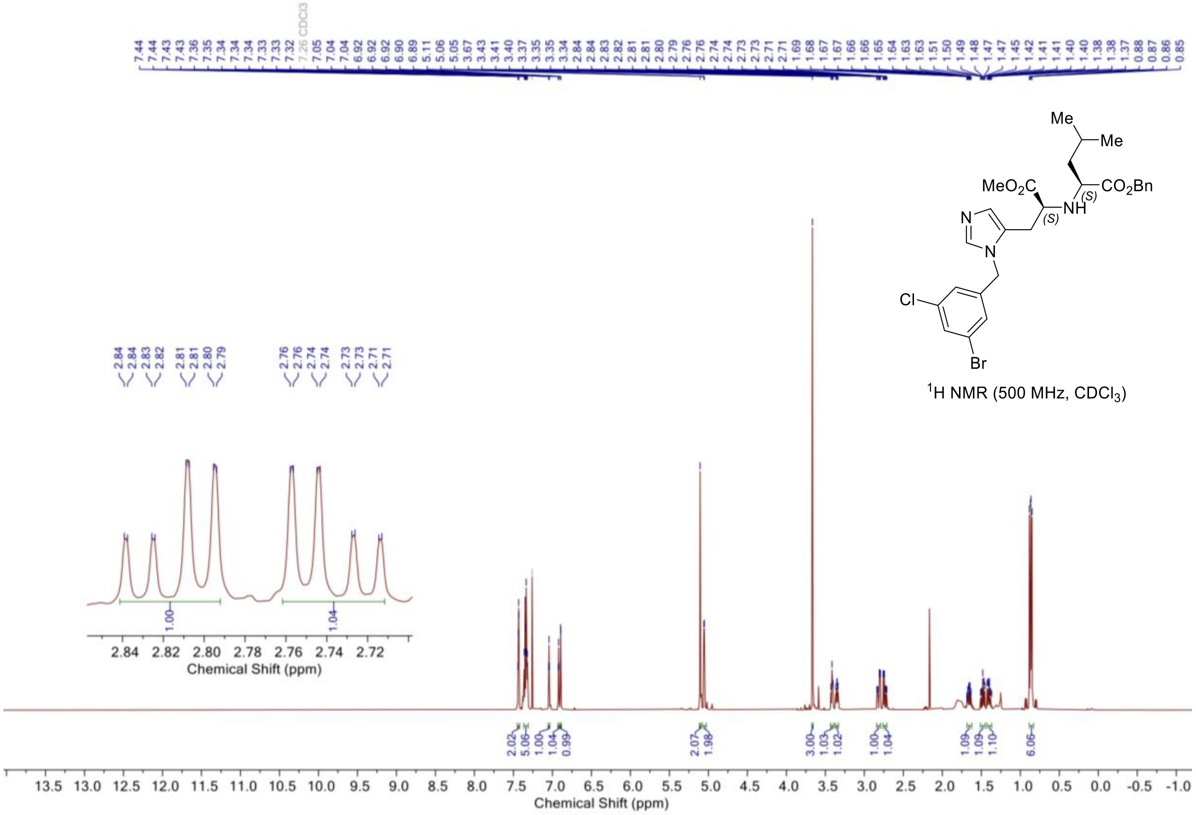

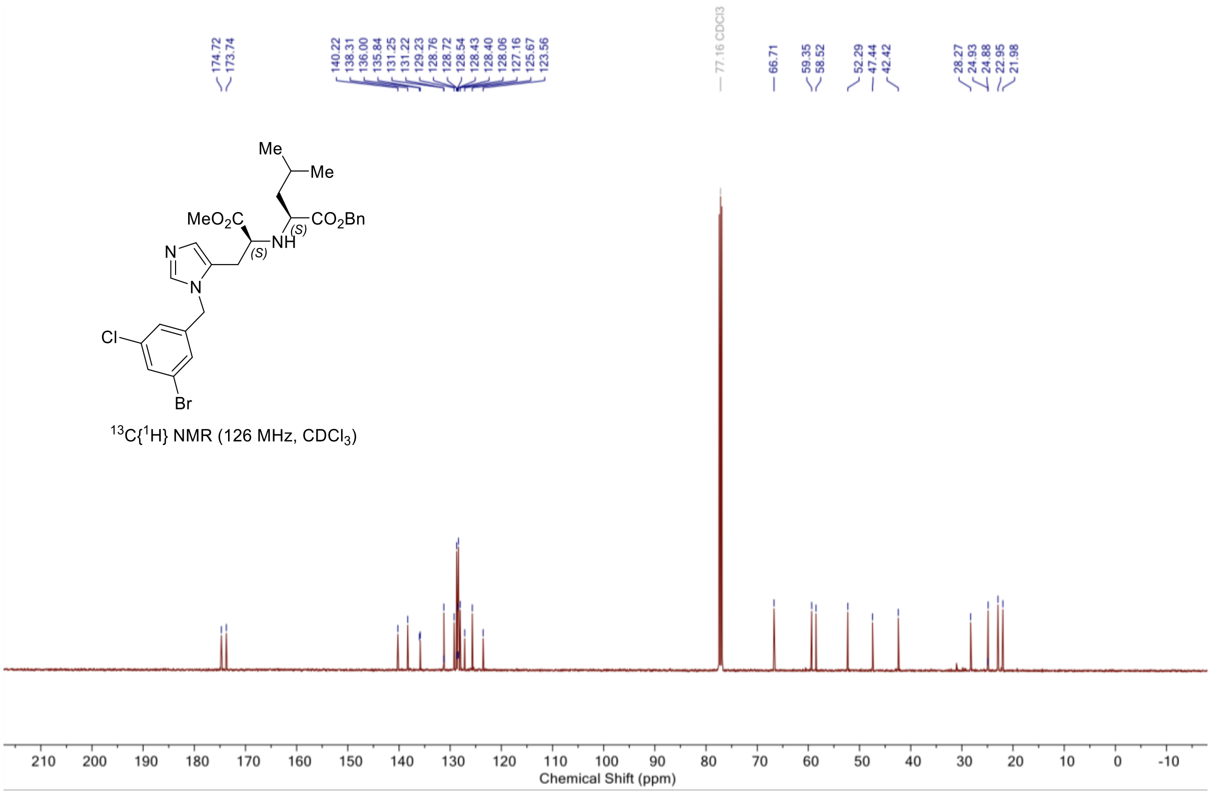

Supplement: Supplementary file 1 [file jo5c00918_si_001.pdf]
